# Edge Decoration of Anthracene Switches Global Diatropic Current That Controls the Acene Reactivity

**DOI:** 10.1021/acs.orglett.1c03605

**Published:** 2021-12-06

**Authors:** Arnab Dutta, Wojciech Stawski, Monika Kijewska, Miłosz Pawlicki

**Affiliations:** † Faculty of Chemistry, Jagiellonian University, Gronostajowa 2, 30-387 Kraków, Poland; ‡ Department of Chemistry, University of Wrocław, F. Joliot-Curie 14, 50383 Wrocław, Poland

## Abstract

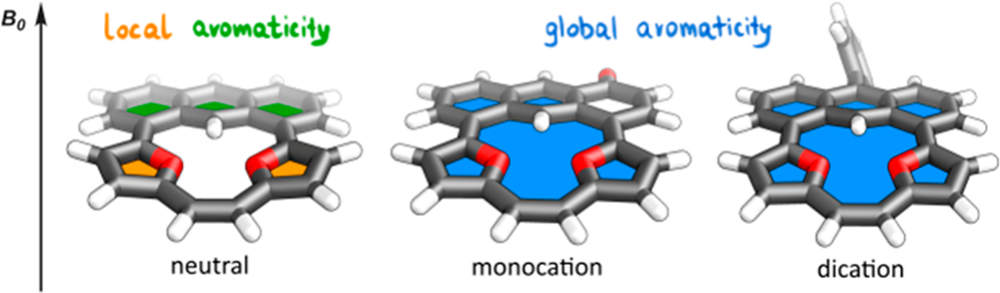

The 14π-electron system of anthracene has been merged with the unsaturated *Z*-1,2-difurylethene to form a macrocycle(s) with the retained local conjugation of all incorporated subunits that were substantially modulated with a redox activation, opening a global delocalization involving all integrated aromatics. In addition, the edge modulation of acene via the attachment of a specific isomer of the conjugated system gives steric confinements that are characteristic of small macrocycles, forcing substantially short C(H)···O electrostatic interactions that are documented spectroscopically with the support of X-ray analysis.

The modulation of π-conjugation within complex unsaturated hydrocarbons remains a pivotal aspect of modern chemistry focusing on the controlled modification of the finally observed properties and reactivities. The linking of two or more unsaturated hydrocarbons in one motif opens the possibility for a postsynthetic modulation of the available π-cloud that leads to changes in the character of the diatropic–paratropic couple,^1^ including switching on the global delocalization.^2^ The changes within π-electrons available in the system can be introduced with fundamental activators and incorporated not only in linear structures but also in macrocyclic motifs.^3^ The redox activation remains a key approach for the modulation of delocalization, also introducing a global effect on the nanoscale,^4^ which is responsible for the modification of the properties of structures incorporating polycyclic aromatic hydrocarbons (PAHs).^5,6^ Acenes, the linearly extended PAHs (e.g., anthracene), remain key players for the controlled modification of the optic response, but because of their substantially extended delocalization, they keep local aromatic character and resist modulation of their character.

The archetypal motif of anthracene, the elongated acene, was explored as a building block incorporated into macrocyclic skeletons with the linear and the edge mode (Figure 1). The linear mode, reported in two variants of mutual influence of two π-clouds, gave a specific interaction showing a competition between the local and global effects of delocalization.^1,7^ In contrast with this, the edge mode led to the full assimilation of both π-systems and a substantial extension of conjugation, influencing the optical properties,^6^ which could be further modulated via the redox activation of furan-based structures.^8^ On the contrary, the geometric constraints introduced in specifically constructed motifs gave a field for testing the reactivity that was unavailable without a macrocycle.^9^ It could also force a close proximity of units in the cavity, resulting in, for example, strong hydrogen bonding.^10^ Thus the macrocyclic confinements present the potential for opening global conjugation,^8^ which can influence all involved subunits, including acenes, that, depending on the orientation, can serve as donors for a dopant entrapment^2^ and also introduce donors/acceptors for an unprecedented interaction.

**Figure 1:**
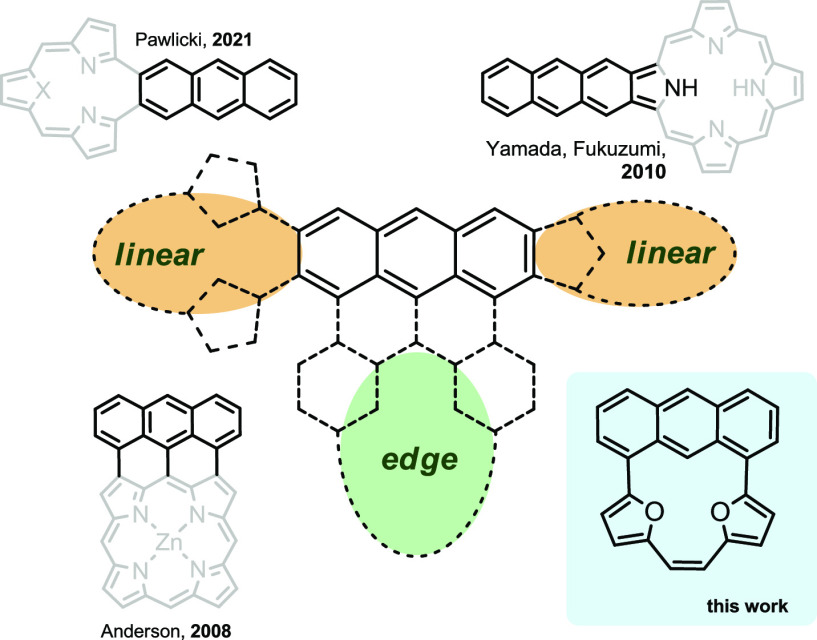
Acene–macrocycle hybrids.

Herein we report on the edge decoration of anthracene with furans, leading to locally delocalized macrocycles and limited mutual interaction between unsaturated subunits. The final molecules readily undergo a redox activation, giving a global diatropic current. The macrocyclic confinements force a proximity between C(H) and O pointing into the cavity and stabilizing a substantially short and unprecedented electrostatic C(H)···O interaction.

The anthracene derivatives were obtained with the application of catalytic processes with transition metals. The connection between acene and heterocyclic subunits was achieved with Suzuki–Miyaura coupling followed by the McMurry reaction as a convenient way to form alkenes utilized in the synthesis of different macrocycles armed with a C=C double bond.^9,11^

The starting material of 1,8-dibromoanthracene (**2a**) is commercially available and was directly applied to Suzuki–Miyaura coupling to form **3a** (Scheme 1, path a, yield 43%), which, followed by the McMurry reaction, gave expected structure **1a** (Scheme 1, path b, yield 45%) as a single macrocyclic product. **1b** was obtained using the same synthetic approach applied for **2b**, which was obtained from the known 1,8-diacetoxy-10-bromoanthracene.^6^ The triflate activation in **2b** led to **3b** (63% yield), which was subjected to the McMurry reaction, giving **1b** in 35% yield. The UV–vis absorption maxima were recorded at λ = 470 (**1a**) and 480 nm (**1b**). Both derivatives remained mute in fluorescence, which became a characteristic feature of macrocycles entrapping a strong H-bond-like interaction within the coordination cavity.^9b,12^

**Scheme 1:**
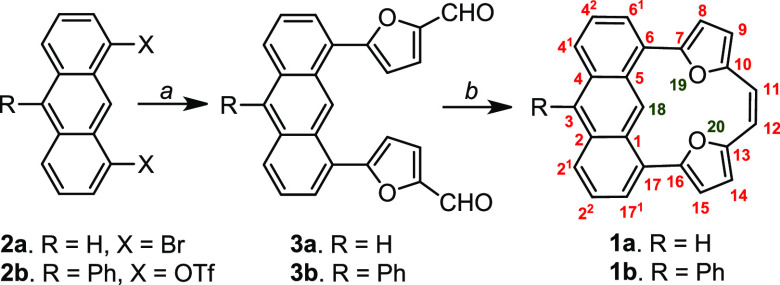
Synthesis of the Edge-Extended Anthracenes Conditions: (a) (5-formylfuran-2-yl)boronic acid (3 equiv), Pd(PPh_3_)_4_ (0.1 equiv), K_2_CO_3_ (10 equiv), THF/H_2_O; (b) TiCl_4_ (38 equiv), Zn (75 equiv), CuI (2 equiv), THF, reflux.

The crystal structure of **1a** showed the presence of three independent molecules in an asymmetric unit with different planarities forced by the packing mode (Figure 2, left) and average deviations from the mean plane of 0.05, 0.11, and 0.21 Å (Figure 2, left). The asymmetric part in the unit cell observed for **1b** showed two independent molecules with different deviations from the mean plane of the main motif of 0.12 and 0.16 Å (Figure 2, right). In addition, **1b** showed a tetrameric packing (Figure 2, right) influenced by the addition of phenyl. The confinements of the macrocyclic structure forced short distances between the two oxygen atoms (O(19) and O(20)) in **1a** (2.771(2) to 2.772(2) Å, Figure 2, left), whereas in **1b**, the same distance was recorded as 2.749(7) and 2.740(7) Å. As reported for different donor–acceptor systems, the carbon–oxygen distance is typically <3.2 Å.^13^ The crystal structure of **1a** showed the C(H)···O distances differing while passing from one molecule to another and increasing with the degree of deviation from planarity. The shortest C(18)–O(19,20) distance (2.666(2) Å) was observed for the most planar variant (Figure 2, left) and increased to 2.710(2) Å for the most ruffled variant. In **1b**, the C(H)···O distances were even shorter (shortest 2.651(7) Å and longest 2.683(7) Å)). Thus the macrocyclic confinement forced the stabilization of the short C(H)···O interaction compared with examples reported to date.^13^ The distance observed in **1a** and **1b** was noticeably shorter than those previously reported for other motifs introducing a specific defect into strongly extended π-cloud.^2^

**Figure 2:**
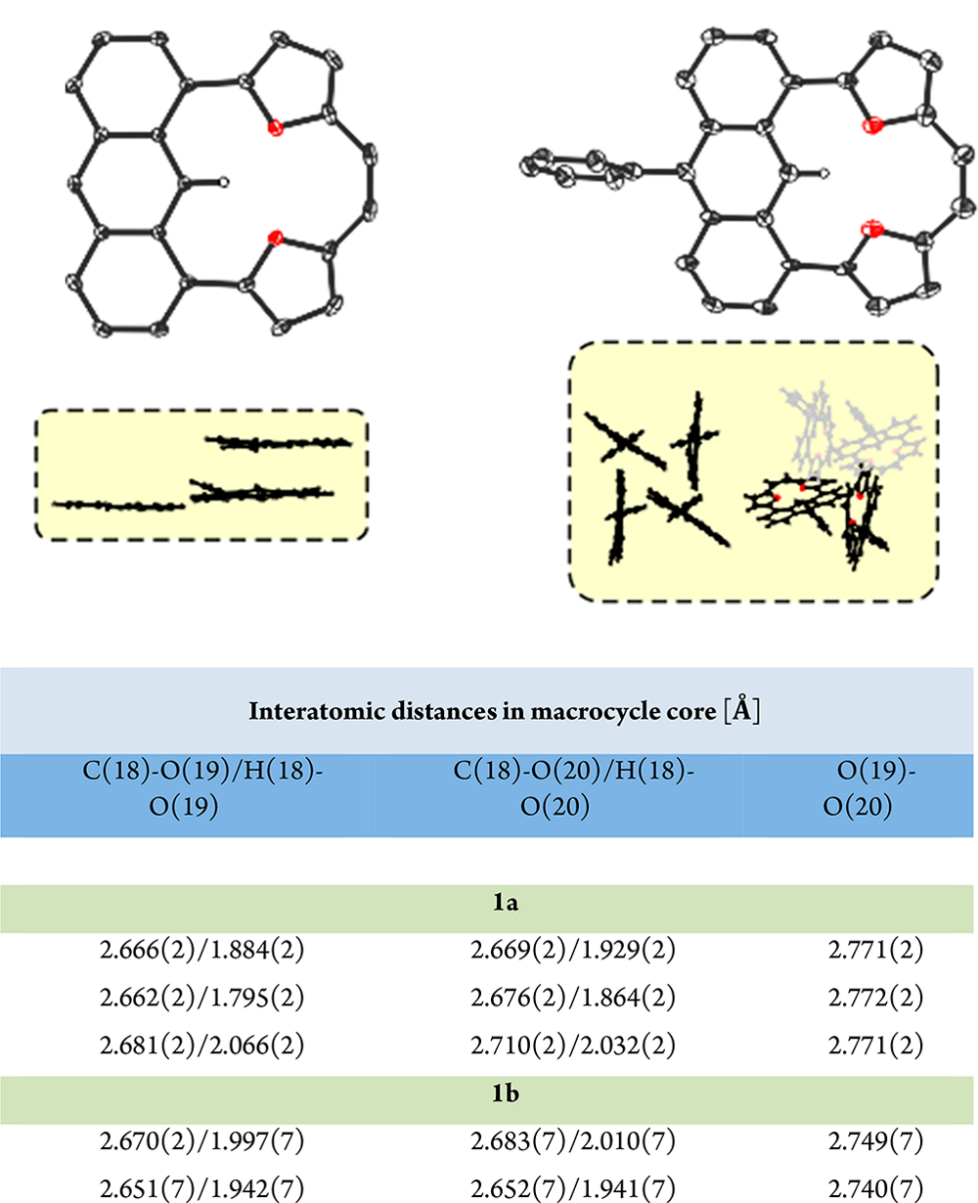
Crystal structures of **1a** (left) and **1b** (right).

The ^1^H NMR spectra create a very sensitive tool for the assessment of the presence of a strong H-bond-like interaction in the analyzed skeleton.^1,2^ The detailed analysis of the ^1^H chemical shifts recorded for H(18) for both couples **3a**/**1a** and **3b/1b** showed a downfield relocation of H(18) by Δδ 3 (from δ 9.8 (**3a**) to δ 12.8 (**1a**); from δ 9.67 (**3b**) to δ 12.71 (**1b**)) that is characteristic of the presence of a strong C(H)···O interaction documented in the crystal structure. Significant downfield-shifted internal signals of all macrocyclic skeletons suggest the presence of a short interaction responsible for a deshielding influence.^1,2^**1a/b** remains locally aromatic according to the magnetic criterion,^14^ as the chemical shifts recorded for all resonances are recorded in the range characteristic of isolated heterocycles (δ 6.5 to 7) and carbocycles (δ 7 to 8), which is consistent with a domination of local currents (Figure 3A).

**Figure 3:**
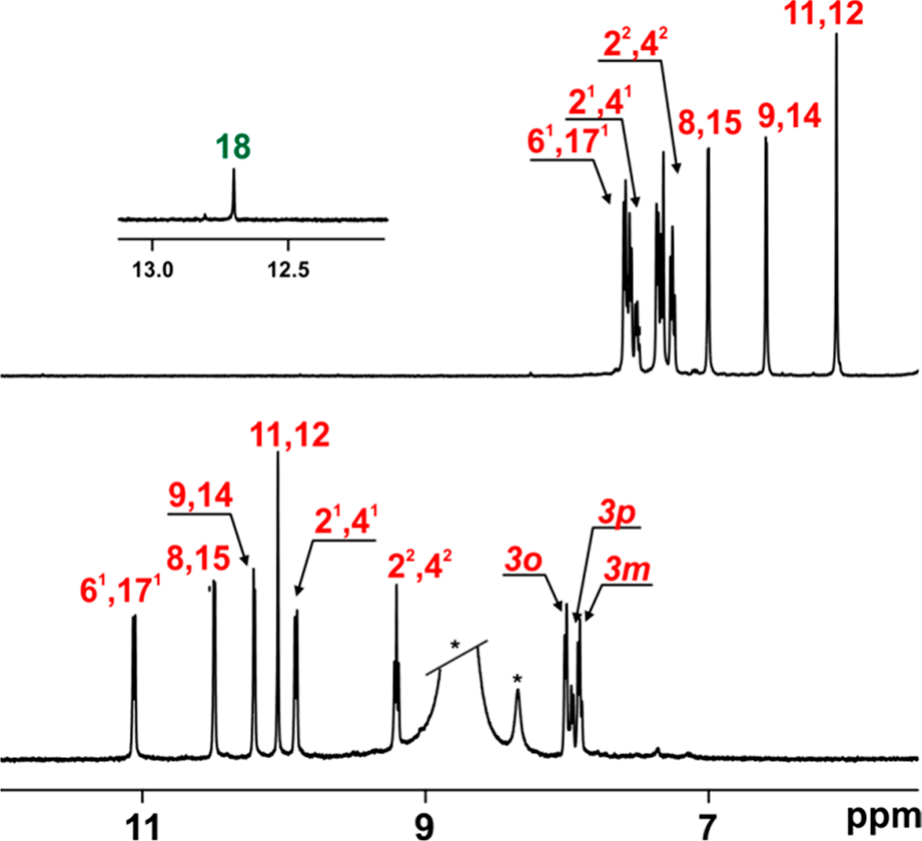
^1^H NMR spectra of **1b** (A, CD_2_Cl_2_, 250 K, 600 MHz) and **1b**^**2+**^ (B, CD_2_Cl_2_, 250 K, 600 MHz). Assignment follows the numbering pattern presented in Scheme 1.

Limiting the conjugation in the target molecule potentially creates a system that can reveal the hidden potential of incorporated subunits finally activating global effects.^1−3^ The competition between local and global delocalization was observed for hybrids where an acene unit was connected to a redox-switching macrocycle, changing the character from aromatic to antiaromatic,^1e^ and also for nanoring systems modified by a multistep redox process.^3a,3b^ The total number of π-electrons (28) suggests that in the case of a global conjugation, **1a**/**b** should have an antiaromatic delocalization (4*n* for *n* = 7). For acene-containing hybrids merged with macrocycles for 4*n* π-systems, different delocalization paths were observed, with a significant contribution of local conjugation(s),^1e^ which, after oxidation, introduced a global conjugation. To test the potential for the modulation of the anthracene π-electron system to contribute to global conjugation, we tested oxidation processes. The UV–vis-monitored titration with nitrosonium hexafluoroantimonate (NOSbF_6_) performed for **1a** showed the appearance of a bathochromically shifted transition (λ ≈ 1000 nm) that gradually disappeared, eventually forming a spectrum shape that was characteristic of a triphyrin-like core with absorbance at λ = 670 nm (Figure S52). The ^1^H NMR-monitored titration performed under the same conditions as the UV–vis experiment (rt) showed a complicated transformation where the spectrum initially disappeared after the addition of the first equivalent of oxidant, which was consistent with a single-electron process and the formation of a radical cation (Figures S20 and 21). Further titration gave a fully asymmetric spectrum that was characteristic of a strongly diatropic molecule with substantially downfield-shifted resonances of the perimeter hydrogen atoms to δ 10-8.5 and the signal of H(18) relocated to δ −1.47. The MS spectrum contained a monocationic signal at *m*/*z* = 349.0845, which was consistent with the accommodation of an additional oxygen atom eventually assigned to **4a** (Scheme 2), with the formation of a carbonyl unit confirmed with the HMBC experiment (^13^C δ 182.8). A C(H)=C(H) bridge was recorded as the AB spin system with a coupling constant of ^3^*J* = 11.8 Hz. **4a** underwent further reaction and accommodated a nitro substituent with NOSbF_6_,^15^ quantitatively forming **5**. Thus **1a** efficiently converted to the fully delocalized system **4a**, where a global diatropic current appeared, and all attempts, including low-temperature experiments, to observe **1a**^**2+**^ were met with failure. Relying on the expected steric protection of the carbocycle subunit by a phenyl in **1b**, we expected the double-charged skeleton to be shielded from further reactivity. The ^1^H NMR-monitored titration performed at rt showed, similarly to **1a**, the disappearance of all signals after the first equivalent of NOSbF_6_ that appeared after the second equivalent of the oxidant (Figure 3,B; for the full titration, see Figure S21). In contrast with **1a**, **1b** reacted with 2 equiv of oxidant to quantitatively form **1b**^**2+**^ (Scheme 2), which could be further stabilized by the application of low temperature. If kept at rt, then **1b**^**2+**^ slowly converted to quantitatively give **4b** (Scheme 2; see the SI), the less sterically crowded isomer of the reactivity documented for the **1a** → **4a** conversion. **1b**^2+^ showed two-fold symmetry in the ^1^H spectrum, with H(18) shifted from δ 12.8 to 0.9, as documented with the HSQC experiment (^13^C = 138.4 ppm). All resonances assigned to the periphery were shifted downfield (δ 10.5–8.5), which is consistent with global delocalization. The C(H)=C(H) bridge was recorded at 10 ppm to be shifted by Δδ = 5 compared with neutral **1b**. Thus the performed oxidation(s) introduced a diatropic current to both charged skeletons that postsynthetically gave derivatives with neither the furan(s) or the ethylene bridge modified but introducing global delocalization.

**Scheme 2:**
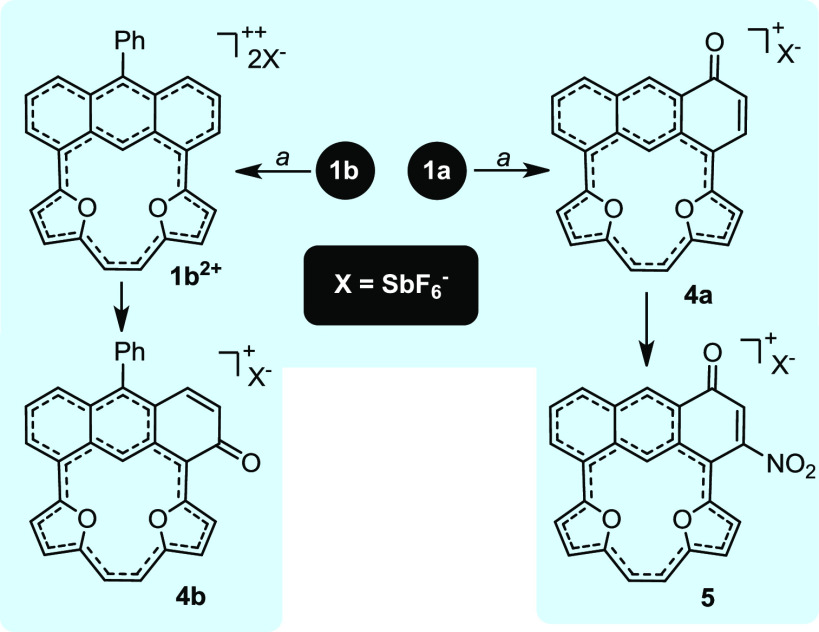
Oxidation Experiments Conditions: (a) CD_2_Cl_2_, 200 K, NOSbF_6_.

The ESP analyses made for fully optimized geometries showed a lack of selectivity based on the charge location, as an equal distribution of positive charge over all molecules of **1a**^**2+**^/**1b**^**2+**^ (Figure S69 and Figure 3, respectively) was documented. Nevertheless, **4a** is not the only regioisomer that keeps the global conjugation (Figure 4). The total energy change in the sequence **4a** < **4a′** < **4a′′** is consistent with the observed selectivity. **1b**^**2+**^ converts to **4b**, observed as a sole isomer, because of the phenyl presence. The gauge independent atomic orbital (GIAO)-predicted NMR parameters (Table S1) show a good correlation with experimental data analyses. All neutral molecules (**1a**, Figure 5) show a dominating contribution of local components with negligible mutual influence. In contrast with this, **1b**^**2+**^ consistently supports a global delocalization covering the whole molecule. **4a** and **4b** show the presence of a global delocalization path (Figures S66 and S67), keeping the newly obtained carbonyl unit isolated. The Atoms in Molecules (AIM) analysis performed for **1a** and **1b** (Figure S71) shows the presence of a bond critical point (BCP) (−3,1) located between H(18) and O(19) or O(20), which is consistent with a postulated strong electrostatic interaction.

**Figure 4:**
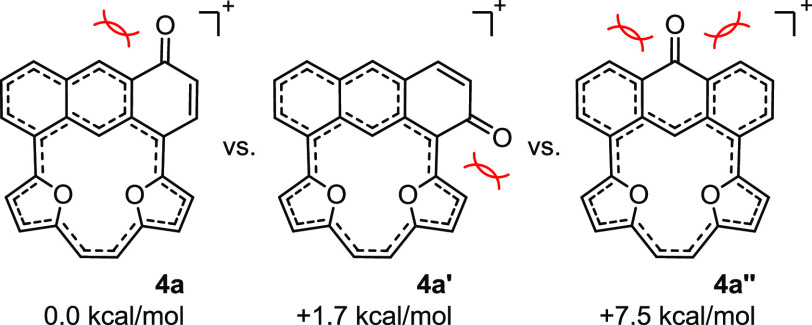
Isomers of the monocationic motif with a built-in carbonyl unit.

**Figure 5:**
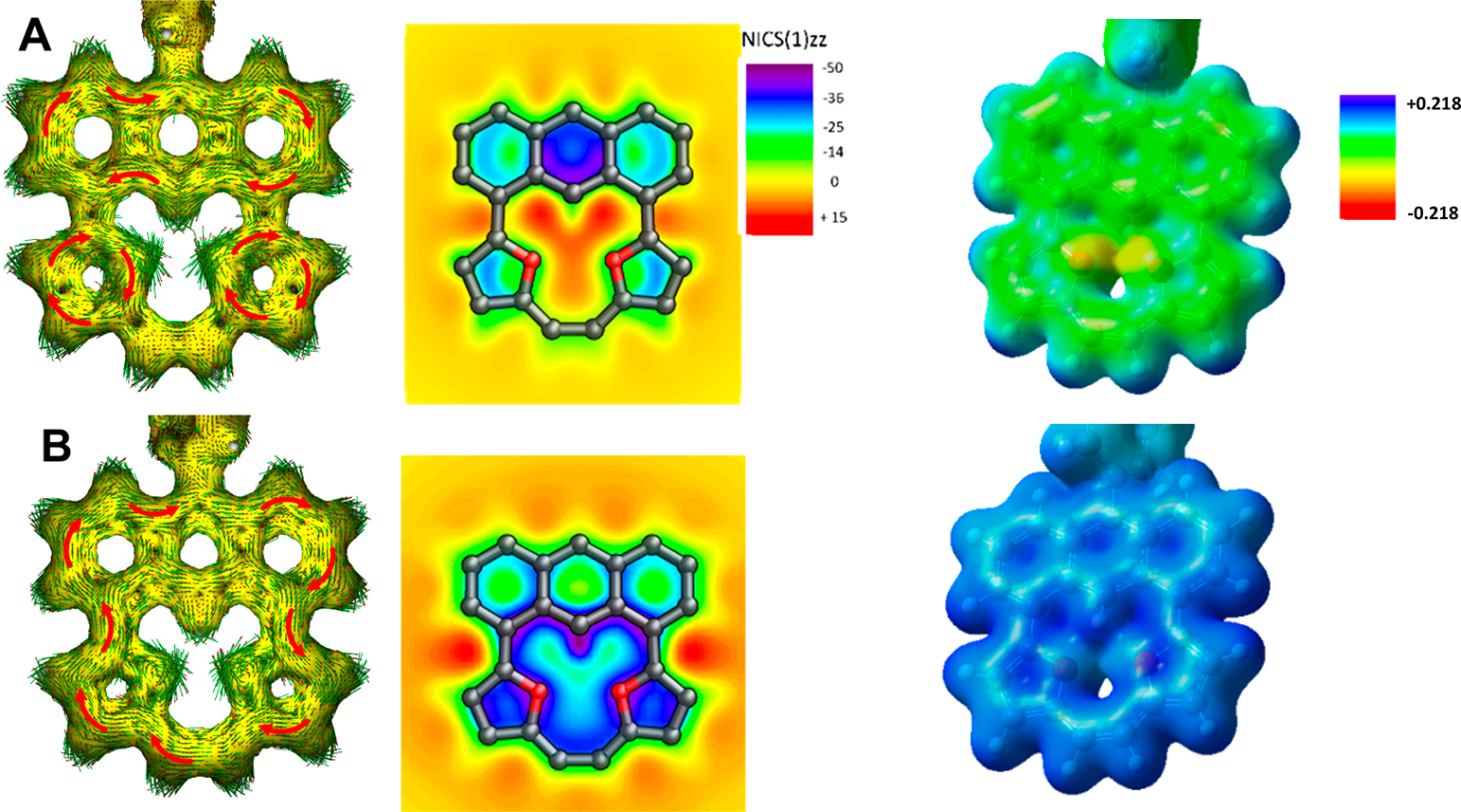
Theoretical analysis (ACID and NICS(1)(zz))^16,17^ and electrostatic potential (ESP) charge distribution of neutral (**1b**, A) and charged (**1b**^**2+**^, B) molecules.

In conclusion, we have designed locally aromatic macrocyclic skeletons predefined for entrapping very strong C(H)···O interactions with an interatomic distance substantially below the van der Waals radii of carbon and oxygen. The step-by-step oxidation leads to globally aromatic structures that spontaneously convert to a carbonyl-containing structure. These findings show the potential of the precise design of macrocycle-decorated acene for controlling the postsynthetic reactivity and switching between local and global conjugation. Currently we are running more experiments in this field.

## Supporting Information Available

General methods, synthesis, and characterization of all compounds (PDF)

## Accession Codes

CCDC 2114821–2114822 contain the supplementary crystallographic data for this paper. These data can be obtained free of charge via www.ccdc.cam.ac.uk/data_request/cif, or by emailing data_request@ccdc.cam.ac.uk, or by contacting The Cambridge Crystallographic Data Centre, 12 Union Road, Cambridge CB2 1EZ, UK; fax: +44 1223 336033.
