# Supplementary material for: Edge Decoration of Anthracene Switches Global Diatropic Current That Controls the Acene Reactivity
Source: Org Lett. 2021 Dec 6;23(24):9436–40. doi: 10.1021/acs.orglett.1c03605 (PMC8689655; doi:10.1021/acs.orglett.1c03605)
Supplement: Supplementary file 1 — ol1c03605_si_001.pdf [file ol1c03605_si_001.pdf]

Edge Decoration of Anthracene Switches Global Diatropic Current that Controls the Acene Reactivity

Arnab Dutta,[‡a] Wojciech Stawski,[‡,§b] Monika Kijewska,[b] and Miłosz Pawlicki[a*]

[a] Faculty of Chemistry, Jagiellonian University, Gronostajowa 2, 30-387 Kraków, POLAND
[b] Department of Chemistry, University of Wrocław, F. Joliot-Curie 14, 50383 Wrocław, POLAND

SUPPORTING INFORMATION

Table of contents

1. General information ... 3
2. Experimental section ... 4
   2.1 Experimental procedures ... 4
3. NMR spectra ... 9
4. UV-VIS spectra ... 36
5. ESI-MS spectra ... 38
6. Theoretical calculation ... 45
   6.1. NICS analysis ... 45
   6.2. AICD plots. ... 48
   6.3. Charge distribution ... 50
   6.4. AIM analysis ... 51
   6.5. Cartesian coordinates ... 52
7. X-Ray Analysis ... 59

1. General information

NMR Spectroscopy. ^{1}H NMR spectra were recorded on a high-field spectrometer (^{1}H 600.15 MHz and 500 MHz, ^{13}C 150 MHz and 125.75 MHz), equipped with a broadband inverse gradient probehead. Spectra were referenced to the residual solvent signal (chloroform-*d*, 7.24 ppm, dichloromethane-d_2 5.32 ppm or acetonitrile-d_3 1.94 ppm). Two-dimensional NMR spectra were recorded with 2048 data points in the t2 domain and up to 1024 points in the t1 domain, with a 1s recovery delay.

Mass Spectrometry. High resolution and Accurate Mass spectra were recorded on a Bruker apex ultra Apex-Qe 7T instrument (Bruker) spectrometer using the electrospray technique. The acetonitrile, dichloromethane or methanol were used as solvents for recording the mass spectra. The potential between the spray needle and the orifice was set to 4.5 kV. The ESI-MS analysis was performed on Shimadzu LC IT-TOF using 0.1% HCOOH in MeCN as a solvent at room temperature (flow rate: 0.1 mL/min).

UV-Vis Spectroscopy. Electronic spectra were recorded on a Varian Carry-50 Bio spectrophotometer.

X-Ray Analysis. X-Ray quality crystals of **1a** and **1b** were prepared by precipitation from DCM/hexane mixture. Diffraction data for both samples were collected on a Kuma KM4 diffractometer equipped with Sapphire CCD detector at 100 K using MoK_α (λ = 0.71073 Å). Data was processed using the CrystAlisPro software. The structures were solved by intrinsic phasing with SHELXT (2015 release) and refined by full-matrix least-squares methods based F^2 using SHELXL. For all structures, H atoms bound to C atoms were placed in the geometrically idealized positions and treated in riding mode, with C-H = 0.95Å and $U_{iso}(H) = 1.2U_{eq}(C)$ for C-H groups.

Theoretical calculations. Geometry optimization for all analysed structures was carried out with the Gaussian 09[1] software package within unconstrained C1 symmetry, with starting coordinates derived from X-ray analysis if available. Becke's three-parameter exchange functional with the gradient-corrected correlation formula of Lee, Yang and Parr (DFT-B3LYP)[2] were used with the 6- 31G(d,p) basis set. The polarizable continuum model of solvation was used (PCM, standard dichloromethane/chloroform/acetone parametrization) for all optimizations. Harmonic vibrational frequencies were calculated using analytical second derivatives as a verification of local minimum achievement with no negative frequencies observed. The AIM analysis was made for fully optimized geometries with MultiWFN package.[3] GIAO predicted chemical shifts for all structures were calculated for fully optimized geometries. The analysis of diatropic currents present in analysed systems were performed via calculation of NICS value and AICD currents calculated with Gaussian 09 software and visualized with the software provided by the Authors. The charge distribution has been performed with NBO calculations and visualized with GaussView 5.0 package.

---

[1] Gaussian 09, Revision E.01; M. J. Frisch et al., Gaussian, Inc.: Wallingford CT, 2009.
[2] a) C. T. Lee, W. T. Yang, R. G. Parr, Phys. Rev. B, 1988, 37, 785-789. b) A. D. Becke, Phys. Rev. A, 1988, 38, 3098-3100.
[3] Tian Lu, Feiwu Chen, *J. Comput. Chem.*, **2012**, *33*, 580-592

2. Experimental section

All solvents (MeOH, Ethyl Acetate, $CHCl_3$, n-hexane, toluene, acetone, water) if not indicated differently were used without purification. CH_2Cl_2 was distilled over CaH_2. All deuterated solvents were used as received except Chloroform-*d* which was prepared directly before using by passing through a basic alumina column. All reactions were performed under inert atmosphere. 1,8-dibromoanthracene (**2a**) was purchased from commercial source and used without any modification.

2.1 Experimental procedures

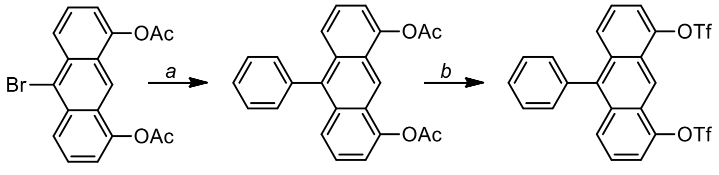

S1 **S2** **2b**

Scheme S1. Synthesis of phenyl substituted anthracene. Conditions: a) Phenylboronic acid, $Pd(PPh_3)_4$, Na_2CO_3, toluene, water, reflux, 24 h; b) i) MeOH/CH_2Cl_2, $MeNH_2$, room temperature, 24 h, ii) dry CH_2Cl_2, Et_3N, Tf_2O, room temperature, 24 h.

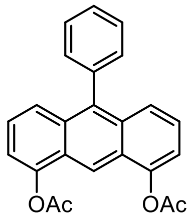

Compound S2. A mixture of compound **S1**[4] (1.33 mmol), phenylboronic acid (2.65 mmol), sodium carbonate (6.17 mmol) and water (9 mL) and toluene (9 mL) were refluxed under argon atmosphere and a solution of $Pd(PPh_3)_4$ (0.064 mmol) in toluene (9 mL) was added rapidly via syringe. After refluxing for 24 hours with heat-on system, the reaction mixture was allowed to cool to room temperature and extracted with CH_2Cl_2. The organic layer was separated and passed through sodium sulfate(VI) and evaporated to dryness. The crude product was subjected to column chromatography with silica using 10% EtOAc in hexane to afford the yellow solid. Yield: 40% (197mg); **^{1}H NMR (500 MHz, $CDCl_3$, 298 K)**, δ(ppm): 8.52 (s, 1H), 7.55 (d, ^3J = 7.2 Hz, 2H), 7.52 (t, ^3J = 7.0 Hz, 2H), 7.51 (d, ^3J = 7.3 Hz, 1H), 7.39-7.37 (d, ^3J = 8.1 Hz, 2H), 7.33-7.30 (t, ^3J = 7.2 Hz, 2H), 7.26-7.24 (t, ^3J = 7.3 Hz, 2H), 2.53 (s, 6H); **^{13}C NMR (126 MHz, $CDCl_3$, 298 K)**, δ(ppm): 169.3, 146.6, 138.3, 138.1, 131.5, 131.1, 128.4, 127.8, 125.4, 125.2, 124.9, 117.2, 113.6, 21.1 ; **HRMS (ESI)**: *m/z*: calculated for $C_{24}H_{18}NaO_4$ $[M+Na]^+$: 393. 1097; found: 393.1074.

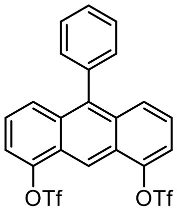

Compound 2b. Compound **S2** (0.27 mmol) was dissolved in MeOH (2 mL) and CH_2Cl_2 (4 mL) and the mixture was degassed for 30 mins by bubbling with argon. Methylamine (2M solution in THF, 1.34 mmol) was added dropwise at 0°C under argon in dark. The mixture was stirred at room temperature for 1 d. The mixture was kept in dark, evaporated, and dried in vacuo. The crude was suspended in degassed dry CH_2Cl_2 (3 mL), followed by addition of Et_3N (2.11 mmol). The mixture was cooled to -13°C, followed by slow addition of Tf_2O (0.87 mmol) diluted with dry CH_2Cl_2 (0.7 mL) with stirring under argon in dark. The mixture was warmed to room temperature and kept for 1 day. After solvent removal by evaporation, the residue was subjected to column chromatography with silica using 10% EtOAc in hexane to obtain yellow

[4] N. K. S. Davis, M. Pawlicki, H. L. Anderson *Org. Lett.* **2008**, *10*, 3945.

solid. Yield: 40% (59 g); **^{1}H NMR** (500 MHz, $CDCl_3$,298 K), δ(ppm): 8.97 (s, 1H), 7.69-7.67 (d, ^3J = 8.8 Hz, 2H), 7.59-7.57 (t, ^3J = 8.0 Hz, 2H), 7.55-7.54 (d, ^3J = 7.5 Hz, 2H), 7.40-7.37 (m, 5H); **^{13}C NMR** (126 MHz, $CDCl_3$, 298 K), δ(ppm): 145.6, 139.1, 137.1, 131.8, 130.9, 128.7, 128.4, 127.8, 126. 9, 125.2, 124.9, 117.9, 113.7; **HRMS (ESI)**: *m/z*: calculated for $C_{22}H_{12}F_6NaO_6S_2$ $[M+Na]^+$: 572.9872; found: 572.9865.

Compound 3a. A mixture of THF (30 mL) and water (10 mL) was degassed via flushing with Ar

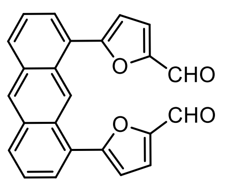

for 30 min. 1,8-dibromoanthracene (174 mg, 0.518 mmol, 1 equiv.), (5-formylfuran-2-yl)boronic acid (218 mg, 1.56 mmol, 3 equiv.), $Pd(PPh_3)_4$ (60.4 mg, 0.052mmol, 0.1 equiv.) and K_2CO_3 (716 mg, 5.18 mmol, 10 equiv.) were added to the mixed solvents. The mixture was refluxed overnight with oil bath. THF was evaporated on a rotary evaporator and the remaining organics were extracted from the remaining water with dichloromethane. After drying over anhydrous Na_2SO_4 and evaporation of the solvent, column chromatography on silica gel was performed. First fractions were eluted with pure dichloromethane and the product was eluted with a mixture of ethyl acetate and dichloromethane (1:1). Recrystallisation from DCM/methanol provided pure 82 mg as a yellow crystalline solid. Yield: 43% **^{1}H NMR** (500 MHz, CD_2Cl_2, 298K) δ 9.73 (s, 1H), 9.72 (s, 1H), 8.65 (s, 1H), 8.18 (dd, ^3J = 8.4, ^4J = 1.0 Hz, 1H), 7.98 (dd, ^3J = 7.0, ^4J = 1.0 Hz, 1H), 7.63 (dd, ^3J = 8.4, 7.0 Hz, 1H), 7.51 (d, ^3J = 3.7 Hz, 1H), 7.27 (d, ^3J = 3.7 Hz, 1H); **^{13}C NMR** (126 MHz, CD_2Cl_2, 298K) δ 177.0, 158.9, 152.5, 131.9, 130.5, 128.6, 128.2, 127.9, 127.2, 125.3, 124.2, 122.0, 112.6; **HRMS (ESI)** *m/z* calculated for $C_{24}H_{14}O_4Na$ $[M+Na]^+$: 389.0784; found : 389.0774.

Compound 3b. A mixture of THF (15 mL) and water (6 mL) was stirred and degassed by argon

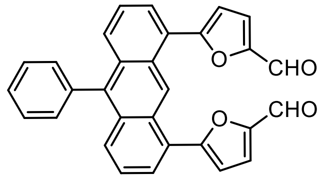

for 1 h. Compound **2b** (0.182 mmol), 5-Formyl-2-furanylboronic acid (0.545 mmol), $Pd(PPh_3)_4$ (0.018 mmol) and K_2CO_3 (1.82 mmol) were added to the mixed solvents. The mixture was stirred and refluxed for 24 hours with heat-on system. The organic solvents were removed under vacuum. The water layer was separated and extracted with CH_2Cl_2. The combined organic layers were washed with brine and dried over Na_2SO_4 and the solvents were evaporated under reduced pressure. Purification of the crude mixture by column chromatography with ethyl acetate/hexane (3:7) solvent mixture afforded the product as yellow solid. Yield: 63% (40 mg); **^{1}H NMR** (500 MHz, $CDCl_3$, 298 K), δ(ppm): 9.72 (s, 2H), 9.67 (s, 1H), 7.90 (d, ^3J = 7.2 Hz, 2H), 7.74 (d, ^3J = 8.9 Hz, 2H), 7.59 (d, ^3J = 7.2 Hz, 2H), 7.48-7.47 (d, ^3J = 3.80 Hz, 2H), 7.44-7.40 (m, 5H), 7.22 (d, ^3J = 3.80 Hz, 2H); **^{13}C NMR** (126 MHz, $CDCl_3$, 298 K), δ(ppm): 176.0, 158.3, 151.4, 137.8, 137.4, 130.1, 129.5, 129.0, 128.4, 127.5, 127.3, 126.9, 126.8, 126.2, 124.0, 121.0, 111.8; **HRMS (ESI)**: *m/z*: calculated for $C_{30}H_{18}NaO_4$ $[M+Na]^+$: 465.1097; found: 465.1091.

Compound 1a. To a 250 mL double-necked round-bottom flask containing 228 mg (3.48 mmol,

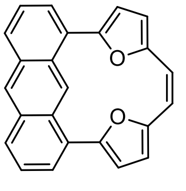

75 equiv.) of Zn dust and 21 mg (0.11 mmol, 2 equiv.) of CuI in 150 mL of dry, degassed THF under inert gas atmosphere, 190 µl of $TiCl_4$ (1.74 mmol, 38 equiv.) was added quickly. The mixture was refluxed for 1 h with oil bath under nitrogen atmosphere and gradual changes of the colour from green to blue or grey were observed. To thus generated low-valent titanium species, a solution of 17 mg of dialdehyde **3a** (0.046 mmol) in 4 mL of degassed, dry THF was added dropwise. The mixture was refluxed for 2 hours and quenched with saturated solution of NH_4Cl,

extracted with dichloromethane, the organic layer was washed with water 2 times, dried with Na_2SO_4 and evaporated. The crude product **1a** was subjected to chromatography using DCM/hex 1:1 (first, orange fraction) and then precipitated from hexane on a rotary evaporator. Yield: 45% (7 mg). **^{1}H NMR** (600 MHz, $CDCl_3$, 298K) δ 12.61 (s, 1H), 8.13 (s, 1H), 7.75 (d, ^3J = 8.5 Hz, 2H), 7.56 (d, ^3J = 7.1 Hz, 2H), 7.34 (dd, ^3J = 8.3, J = 7.1 Hz, 2H), 6.90 (d, ^3J = 3.5 Hz, 2H), 6.49 (d, ^3J = 3.5 Hz, 2H), 6.04 (s, 2H); **^{13}C NMR** (126 MHz, $CDCl_3$, 298K) δ 156.7, 152.5, 132.7, 129.1, 128.9, 128.7, 127.1, 125.8, 123.7, 121.4, 115.6, 112.4, 110.2; **HRMS (ESI):** observed $[M]^{+\cdot}$ m/z = 334.0977, calcd. for $C_{24}H_{14}O_2$ 334.0988; **UV/Vis** (MeCN, 298K, λ, log ε): 252 (4.73), 284 (4.72), 470 (3.89).

Compound 1b. To a 250 mL double necked round bottom flask containing Zn dust (3.42 mmol) and CuI (0.108 mmol) in 80 mL dry, degassed THF under inert gas atmosphere, $TiCl_4$ (1.72 mmol) was added. The mixture was refluxed for 2h under argon atmosphere and gradual changes of colour from green to grey were observed. To this generated low valent titanium (0) species, solution of the compound **3b** (0.045 mmol) in 3 mL of degassed THF was added dropwise. The mixture was refluxed for 1 hour with heat-on system and quenched with 10 % aq. Solution of K_2CO_3 (10 mL), extracted with ethyl acetate. The organic layer was washed with water two times, dried with Na_2SO_4 and evaporated. The crude product was subjected to column chromatography using hexane and reduced under pressure to obtain the desired compound as red powder. Yield: 35% (6.5 mg); **^{1}H NMR** (500 MHz, CD_2Cl_2, 298 K), δ(ppm): 12.71 (s, 1H), 7.65 (d, ^3J = 7.1 Hz, 2H), 7.61-7.59 (t, ^3J = 7.0 Hz, 2H), 7.55-7.54 (d, ^3J = 7.5 Hz, 1H), 7.43-7.42 (d, ^3J = 7.8 Hz, 2H), 7.42-7.41 (d, ^3J = 8.2 Hz, 2H), 7.30-7.27 (dd, ^3J = 8.8 Hz, 2H), 7.02-7.01 (d, ^3J = 3.5 Hz, 2H), 6.60-6.59 (d, ^3J = 3.5 Hz, 2H), 6.14 (s, 2H); **^{13}C NMR** (126 MHz, $CDCl_3$, 298K), δ(ppm): 156.8, 152.6, 138.9, 134.9, 131.3, 131.2, 129.0, 128.6, 128.5, 128.2, 127.4, 125.9, 125.6, 121.5, 115.5, 112.4, 110.4; **HRMS (ESI)**: m/z: calculated for $C_{30}H_{18}O_2$ $[M]^+$: 410.1307; found: 410.1301. **UV/Vis** (CH_2Cl_2, 298K, λ, log ε): 256 (4.29), 287 (4.27), 480 (4.05).

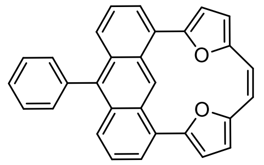

General procedure of oxidation: 0.8 mg of compound (**1a** or **1b**) was dissolved in 400 µL of CD_3CN in a glove-box conditions. To this solution $NOSbF_6$ (20 mg in 500 µl of CD_3CN prepared in glove-box conditions) was added gradually every half equivalent and the reaction was controlled by NMR spectroscopy, ESI-MS analysis and UV/Vis.

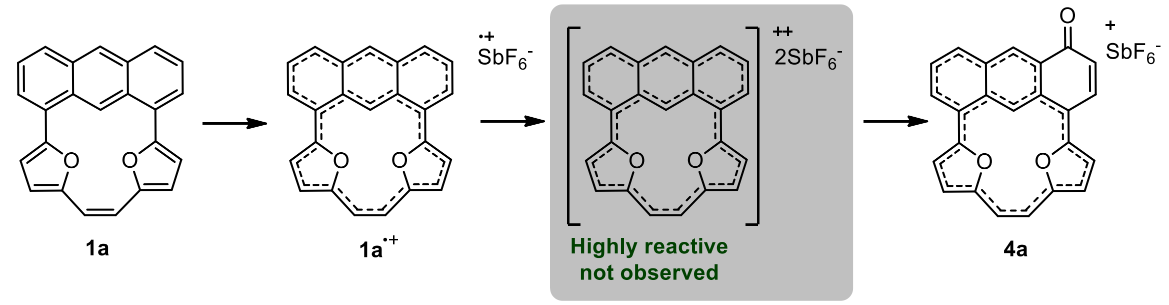

Scheme S2. Oxidation experiments for **1a**.

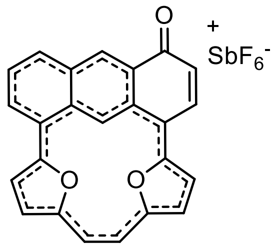

Compound 4a. **^{1}H NMR** (600 MHz, CD_3CN, 298K) δ 9.89 (d, ^3J = 7.6 Hz, 1H), 9.56 (d, ^3J = 4.6 Hz, 1H), 9.48 (d, ^3J = 4.7 Hz, 1H), 9.46 (d, ^3J = 4.7 Hz, 1H) , 9.35 (d, ^3J = 11.8, 1H), 9.18 (d, ^3J = 4.6 Hz, 1H), 9.08 (d, ^3J = 7.6 Hz, 1H), 8.89 (d, ^3J = 11.8, 1H), 8.83 (s, 1H), 8.78 (t, ^3J = 7.5 Hz, 1H), 8.69 (d, ^3J = 9.9 Hz, 1H), 7.02 (d, ^3J = 9.9 Hz 1H), -1.47 (s, 1H); **^{13}C NMR** (126 MHz, CD_3CN, 298K) partial data obtained by correlation experiments HMBC: δ 182.8, 162.8, 161.7, 154.9, 152.9, 138.2, 136.8, 136.5, 134.6, 134.5, 134, 133.9, 131.9, 130.5, 130.2, 123.2, 122.1, 120.3, 107.8; **HRMS (ESI):** observed $[M]^+$ *m/z*: 349.0845, calcd. for $C_{24}H_{13}O_3$ 349.0859; **UV/Vis** (MeCN, 298K, λ):254 (4.57), 659 (3.54).

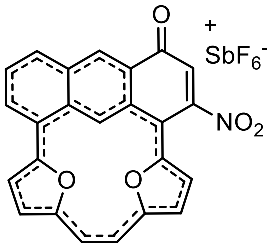

Compound 5. **^{1}H NMR** (600 MHz, CD_3CN, 298K) δ 10.6 (d, ^3J = 7.8 Hz, 1H), 10.01 (d, ^3J = 4.6 Hz, 1H), 9.99 (d, ^3J = 4.9 Hz, 1H) 9.91 (s, 1H), 9.93 (s, 1H), 9.86 (d, ^3J = 4.6 Hz, 1H), 9.81 (d, ^3J = 7.6 Hz, 1H), 9.77 (d, ^3J = 11.7 Hz, 1H), 9.56 (d, ^{3}J = 5 Hz, 1H), 9.3 (d, ^3J = 11.7 Hz, 1H), (9.21 (t, ^3J = 7.8 Hz, 1H), 0.17 (s, 1H); **^{13}C NMR** (126 MHz, CD_3CN, 298K) partial data obtained by correlation experiments HSQC: 139.5, 139.4, 137.8, 136.7, 135.5, 134.4, 133.9, 133.1, 131.1, 125.2, 123.1, 109.2; The increased reactivity for **5** derivative made impossible recording ^{13}C carbon experiment; **HRMS (ESI):** observed $[M]^+$ *m/z*: 394.0713, calcd. for $C_{24}H_{12}NO_5$ 394.0710.

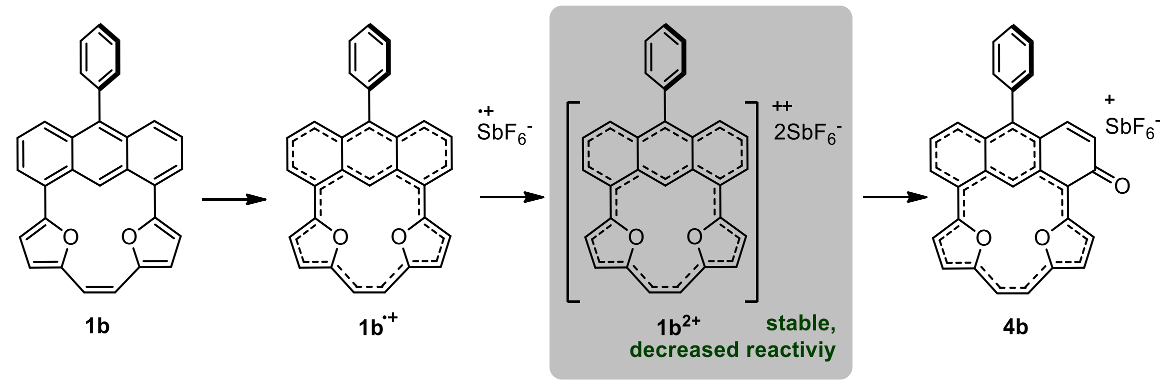

Scheme S3. Oxidation experiments for **1b**.

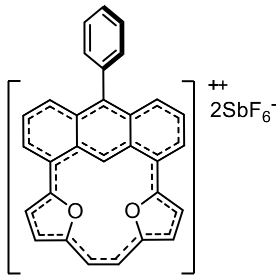

Compound 1b^{2+}. ^{1}**H NMR** (600 MHz, CD_2Cl_2, 193 K) δ 11.04 (d, ^{3}J = 7.9 Hz, 2H), 10.49 (d, ^{3}J = 4.7 Hz, 2H), 10.21 (d, ^{3}J = 4.7 Hz, 2H), 10.02 (s, 2H), 9.92 (d, ^{3}J = 7.9 Hz, 2H), 9.2 (t, ^{3}J = 7.8 Hz, 2H), 8.01-7.85 (m, 5H), 0.9 (s, 1H). ^{13}**C NMR** (126 MHz, CD_2Cl_2, 193 K) partial data based on correlation (HSQC) experiment: 153.7, 153.3, 143.4, 135.8, 134.3, 131.3, 128.8, 120.1. Increased reactivity recorded for **1b**$^{2+}$ which is solely observable at low temperatures eliminated a possibility of recording either mass experiment or elemental analysis for this compound.

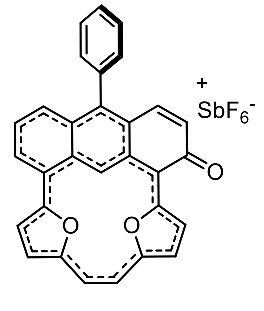

Compound 4b. ^{1}**H NMR** (600 MHz, CD_2Cl_2, 298 K) δ 10.73 (d, ^{3}J = 7.8 Hz, 1H), 10.53 (d, ^{3}J = 4.7 Hz, 1H), 10.26 (d, ^{3}J = 4.3Hz, 1H), 9.97 (d, ^{3}J = 4.3 Hz, 1H), 9.92 (d, ^{3}J = 11.8 Hz, 1H), 9.67 (d, ^{3}J = 4.9 Hz, 1H), 9.48 (d, ^{3}J = 11.8 Hz, 1H), 9.38 (d, ^{3}J = 7.7 Hz, 1H), 9.20 (t, ^{3}J = 7.8 Hz, 1H), 8.28 (d, ^{3}J = 10.2 Hz, 1H, 7.86-7.83 (m, 5H), 7.17 (d, ^{3}J = 10.2 Hz, 1H), -0.52 (s, 1H); **HRMS (ESI):** observed $[M]^+$ *m/z*: 425.1174, calcd. for $C_{30}H_{17}O_3$ 425.1172. The increased reactivity for **4b** derivative made impossible recording of any carbon experiment.

3. NMR spectra

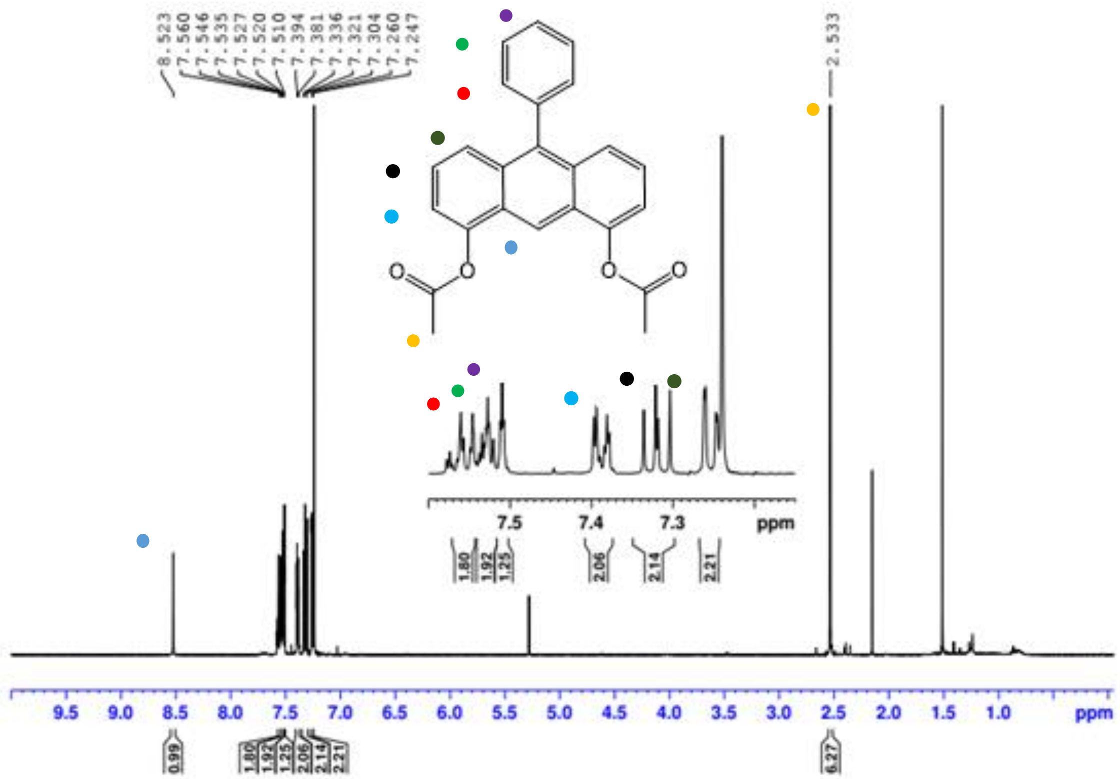

Fig. S1 ^{1}H NMR ($CDCl_3$, 500 MHz, 298 K) spectrum of **S2**.

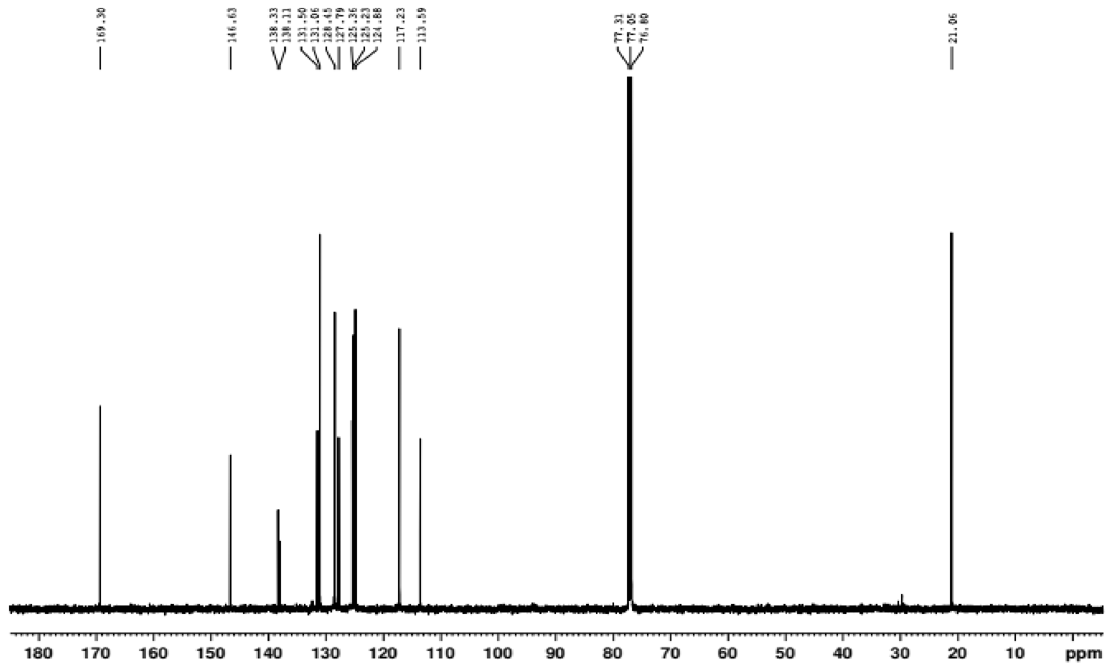

Fig. S2 ^{13}C NMR ($CDCl_3$, 126 MHz, 298 K) spectrum of **S2**.

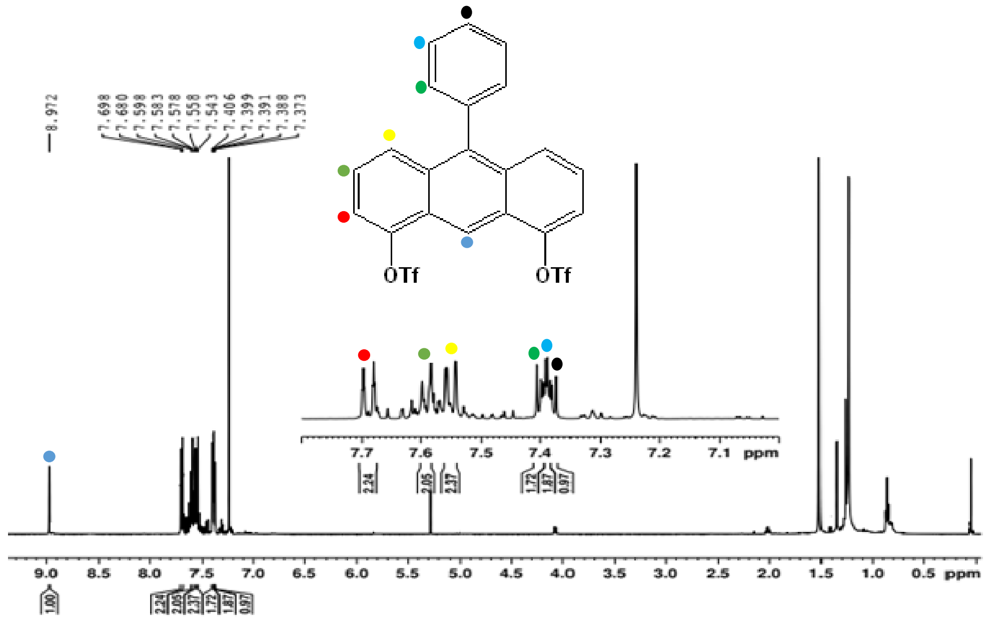

Fig. S3 ^{1}H NMR ($CDCl_3$, 500 MHz, 298 K) spectrum of **2b**.

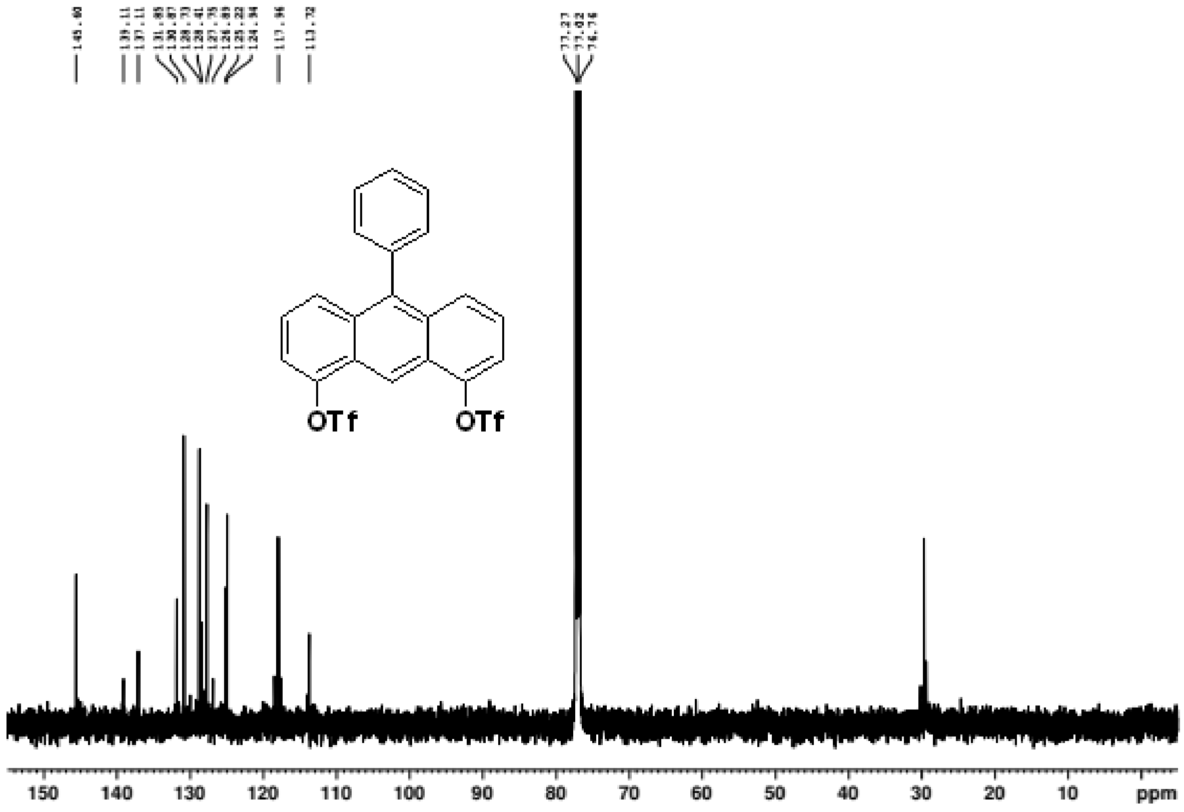

Fig. S4 ^{13}C NMR ($CDCl_3$, 126 MHz, 298 K) spectrum of **2b**.

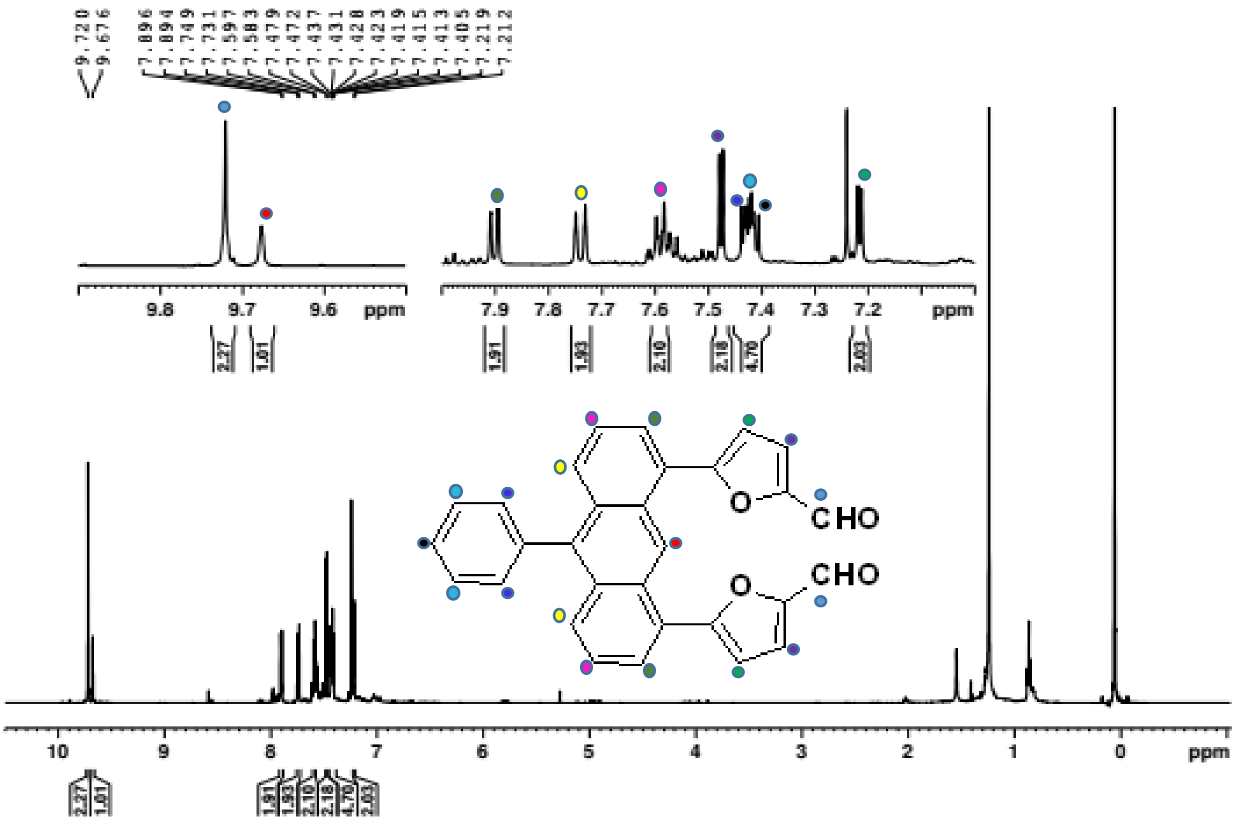

Fig. S5 ^{1}H NMR ($CDCl_3$, 500 MHz, 298 K) spectrum of **3b**.

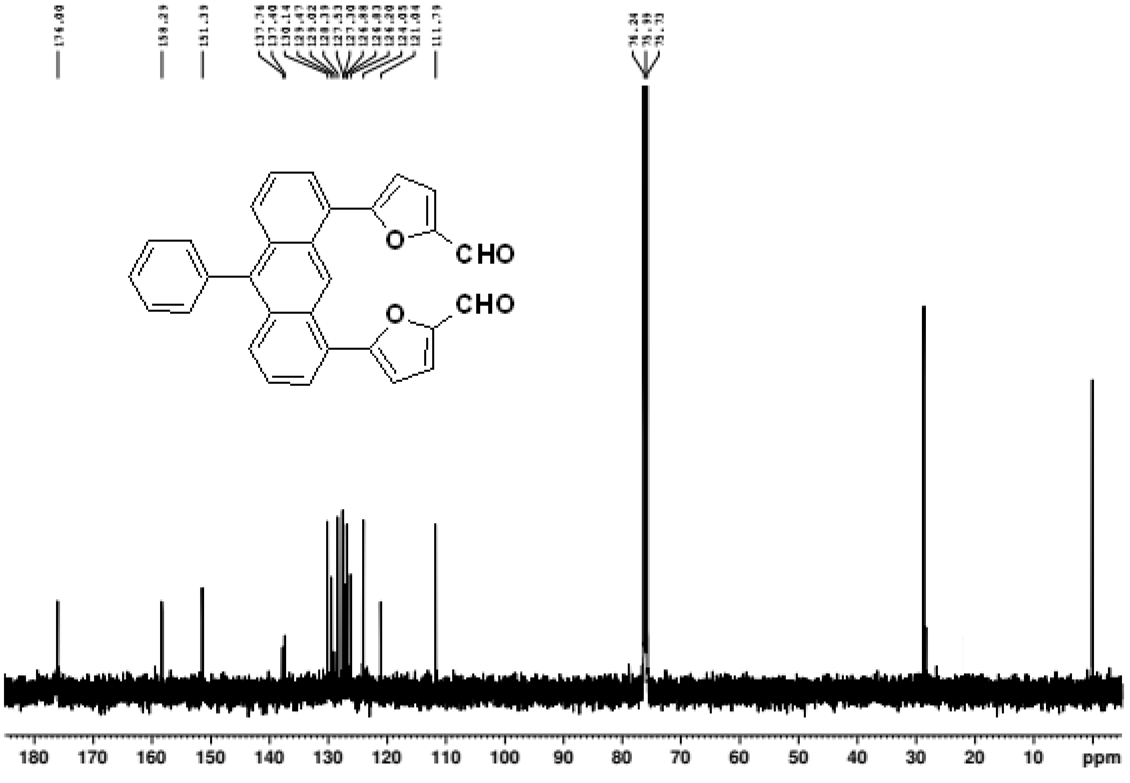

Fig. S6 ^{13}C NMR ($CDCl_3$, 126 MHz, 298 K) spectrum of **3b**.

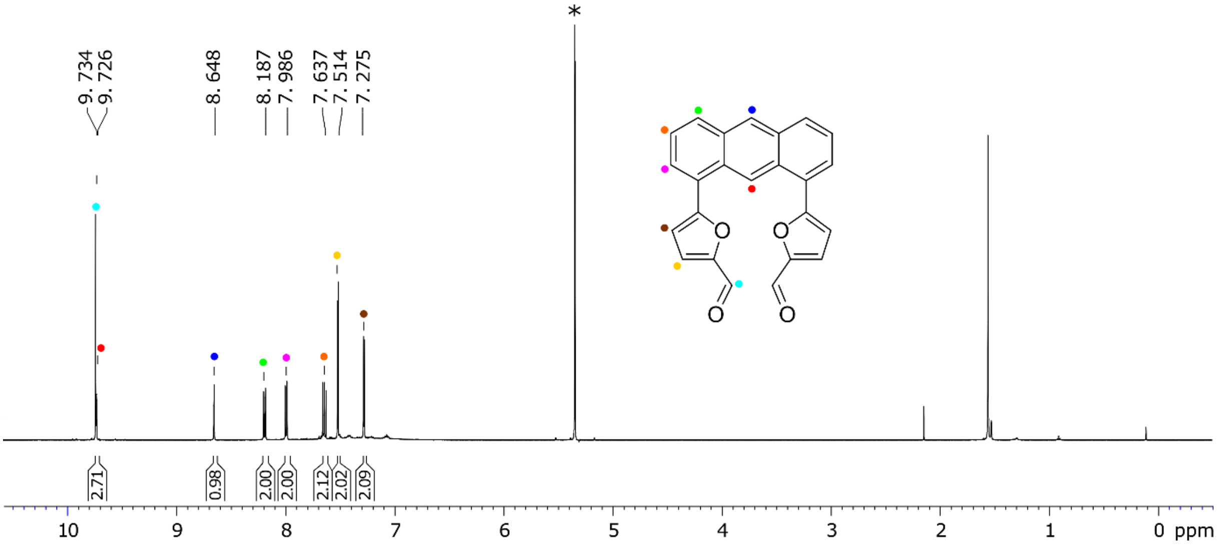

Fig. S7 ^{1}H NMR (CD_2Cl_2, 500 MHz, 298 K) spectrum of **3a.**

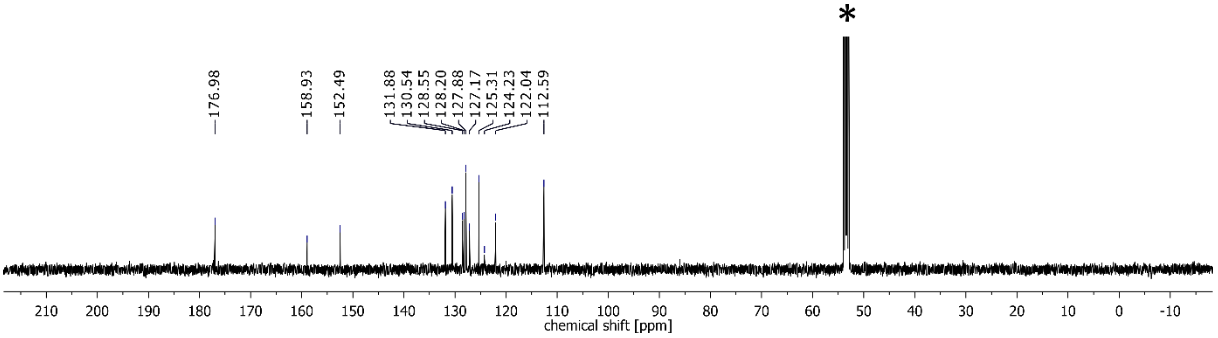

Fig. S8 ^{13}C NMR (CD_2Cl_2, 126 MHz, 298 K) spectrum of **3a**.

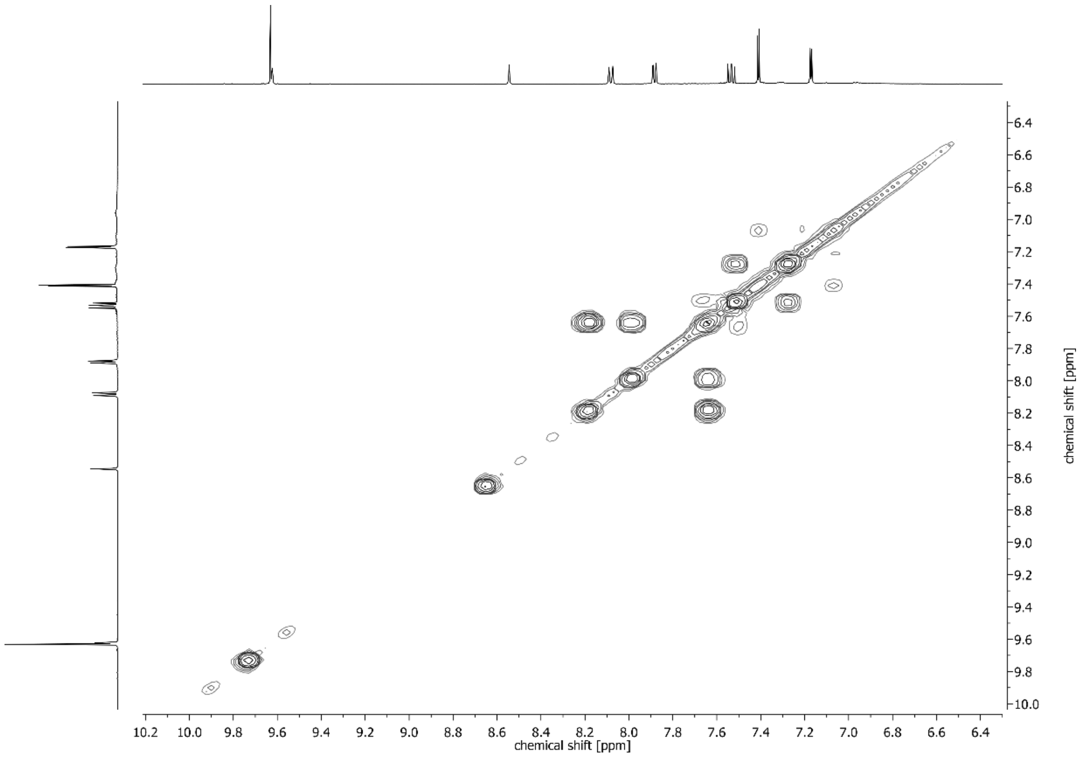

Fig. S9 ^1H-^1H COSY NMR (CD_2Cl_2, 500 MHz, 298 K) spectrum of **3a**.

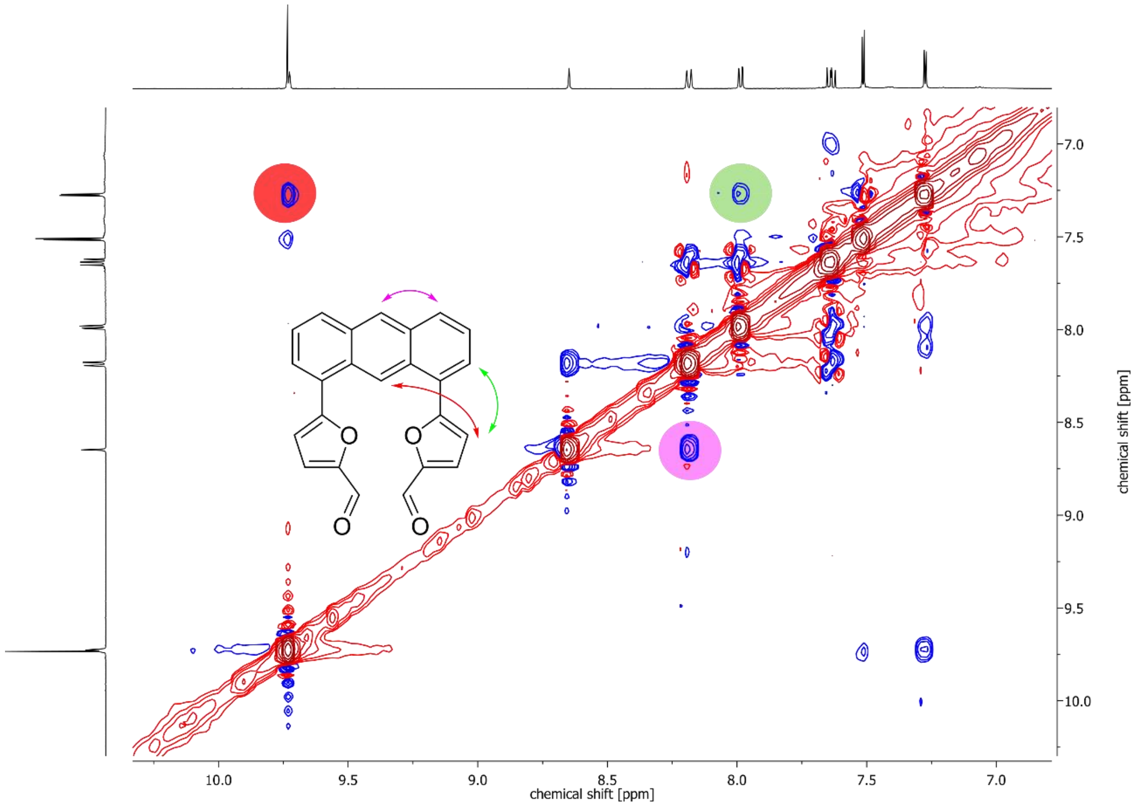

Fig. S10 ^1H-^1H NOESY NMR (CD_2Cl_2, 500 MHz, 298 K) spectrum of **3a**.

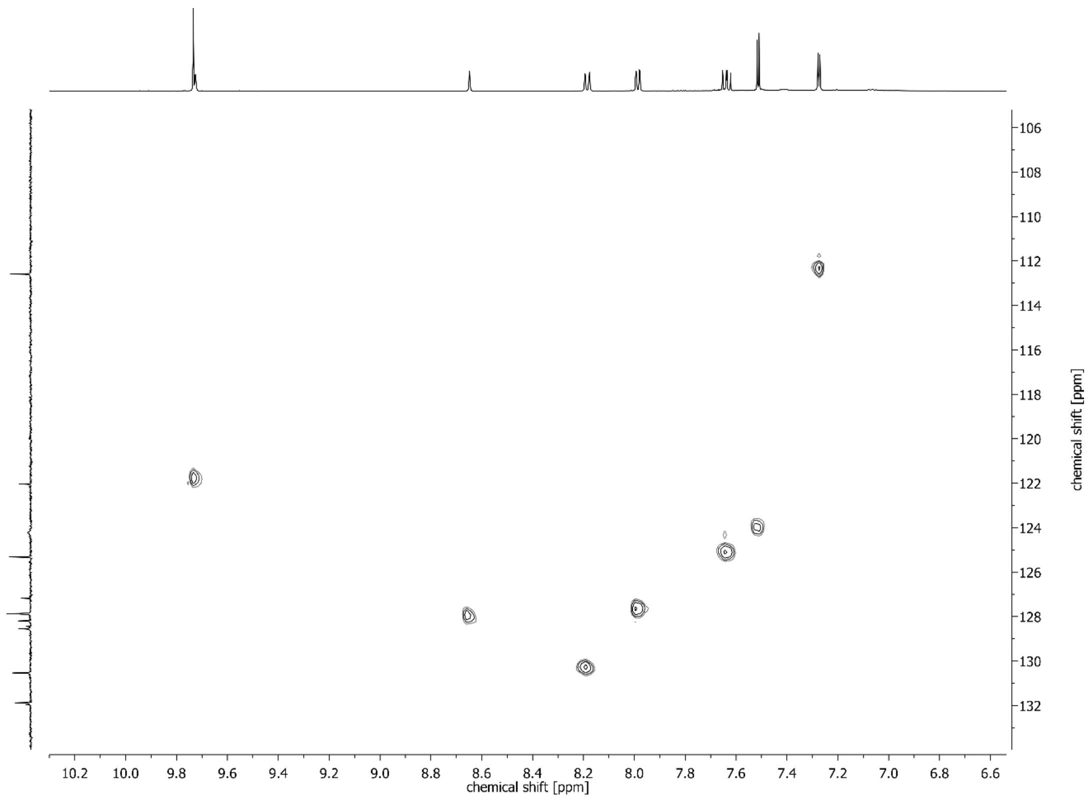

Fig. S11 ^{1}H-^{13}C HSQC NMR (CD_2Cl_2, 500 MHz, 298 K) spectrum of **3a**.

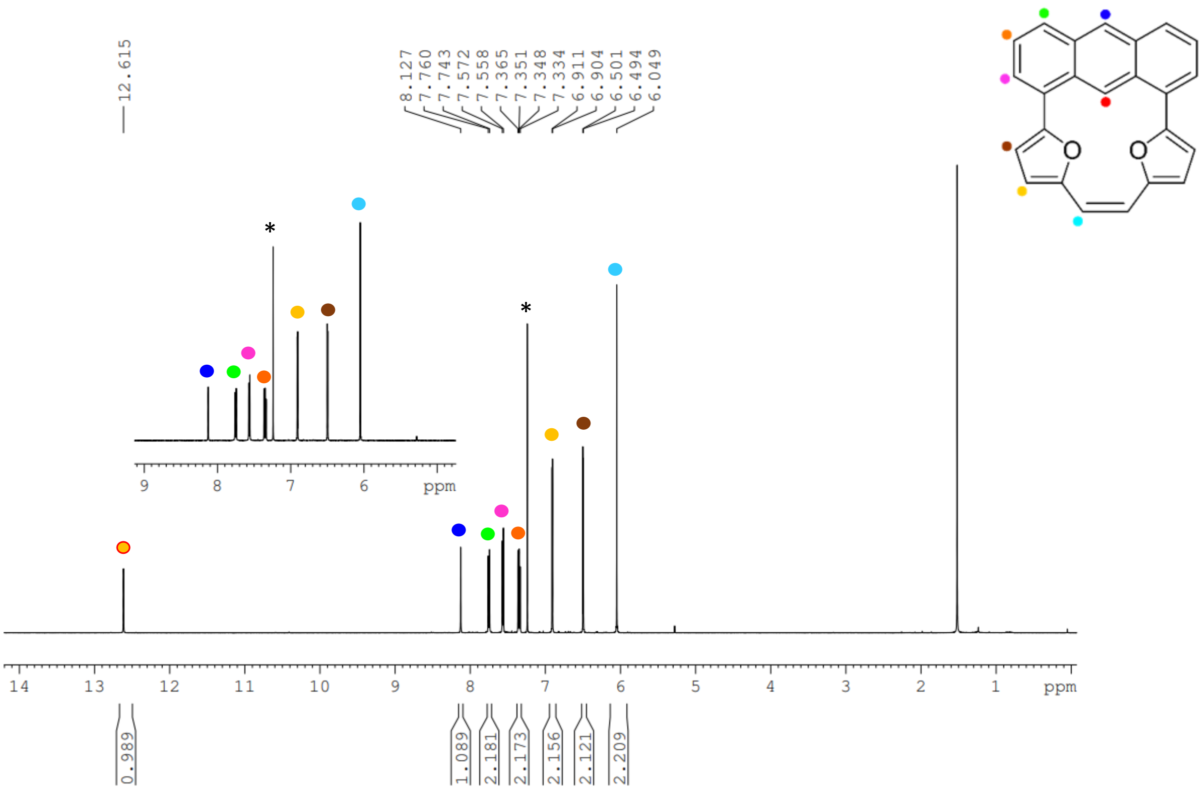

Fig. S12 ^{1}H NMR ($CDCl_3$, 600 MHz, 298 K) spectrum of **1a**.

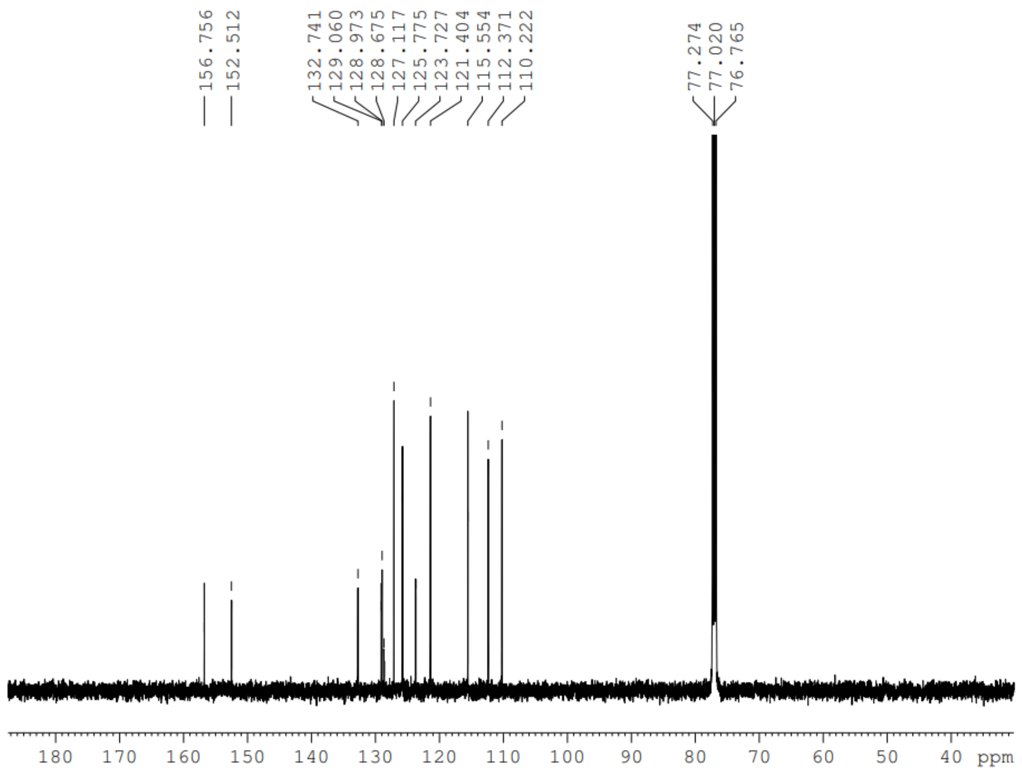

Fig. S13 ^{13}C NMR ($CDCl_3$, 126 MHz, 298 K) spectrum of **1a**.

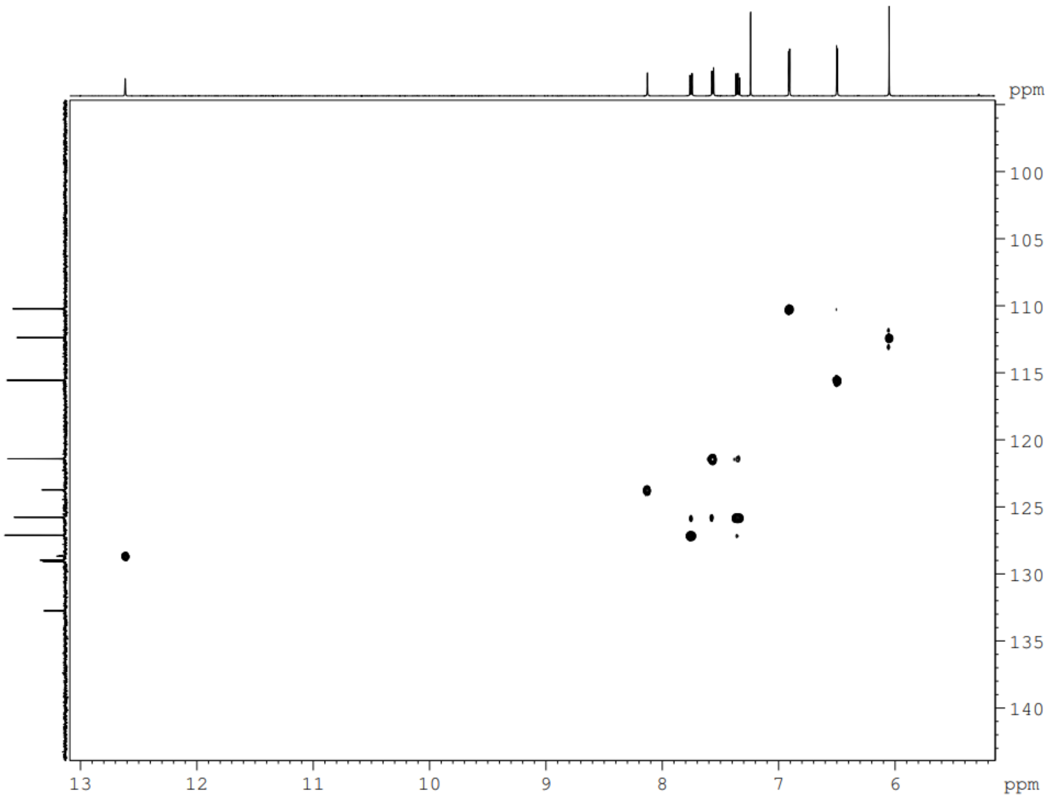

Fig. S14 ^{1}H-^{13}C HSQC NMR ($CDCl_3$, 600 MHz, 298 K) spectrum of **1a**.

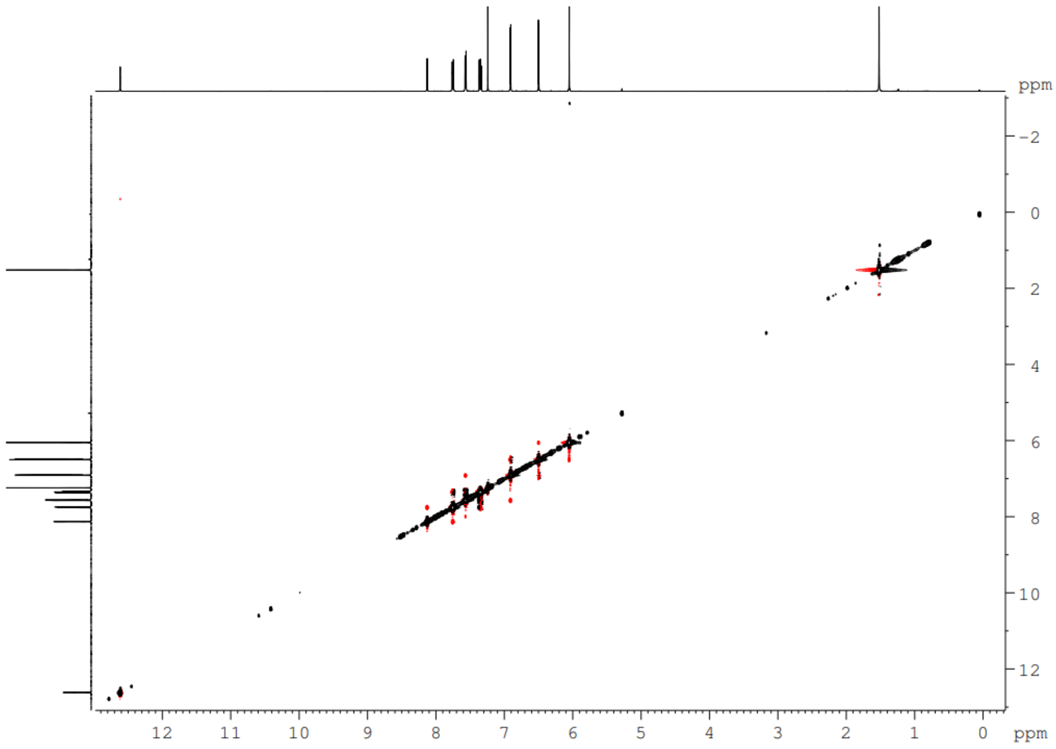

Fig. S15 ^{1}H-^{1}H NOESY NMR ($CDCl_3$, 500 MHz, 298 K) spectrum of **1a.**

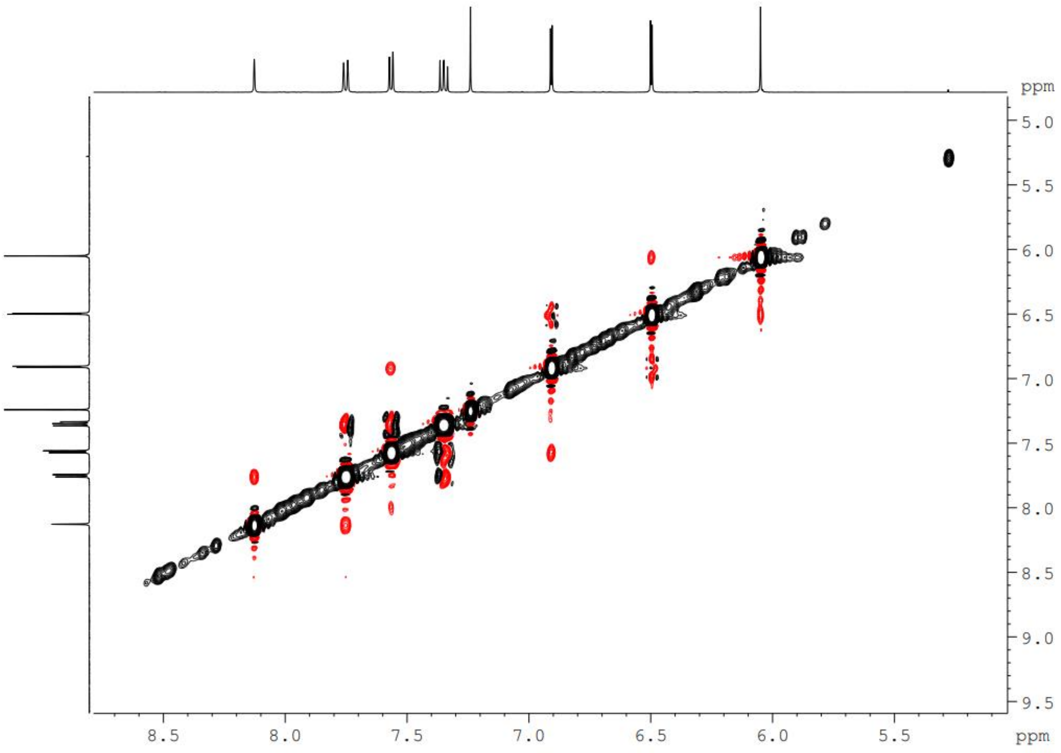

Fig. S16 ^{1}H-^{1}H NOESY NMR (zoom aromatic region) ($CDCl_3$, 500 MHz, 298 K) spectrum of **1a.**

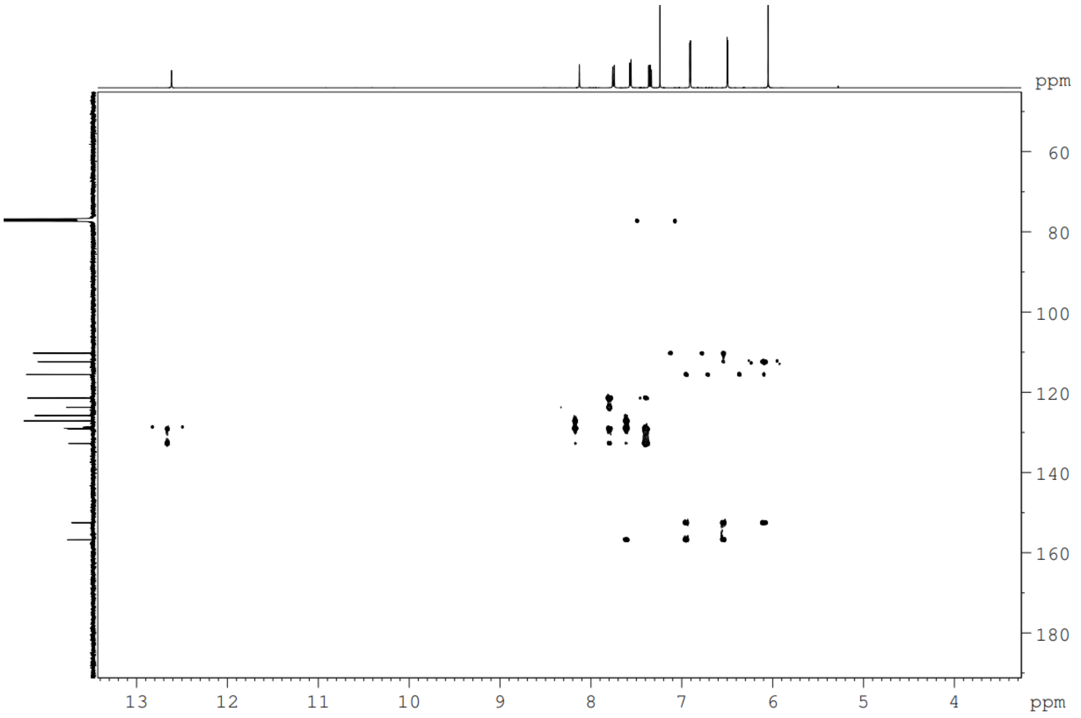

Fig. S17 ^{1}H-^{13}C HMBC NMR ($CDCl_3$, 500 MHz, 298 K) spectrum of **1a.**

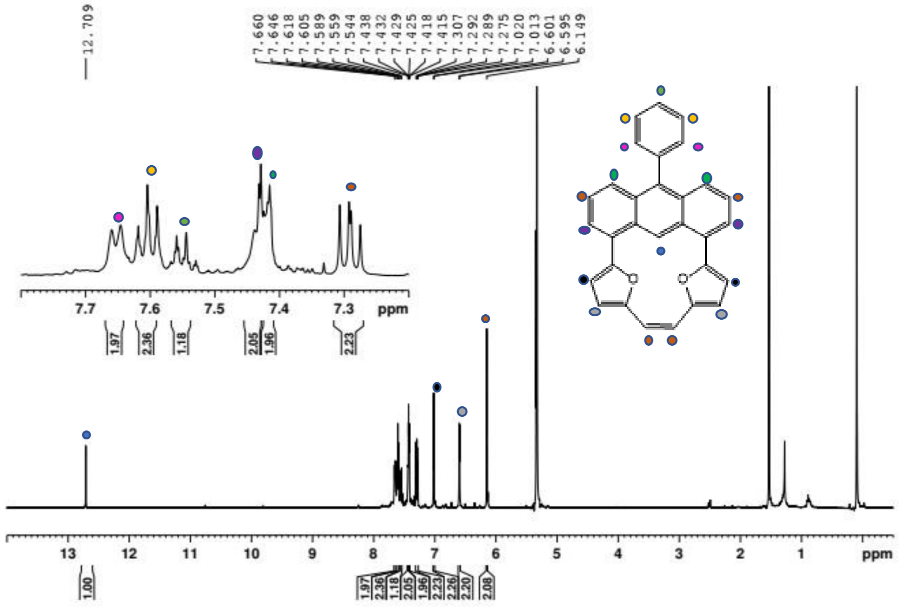

Fig. S18 ^{1}H NMR (CD_2Cl_2, 500 MHz, 298 K) spectrum of **1b**.

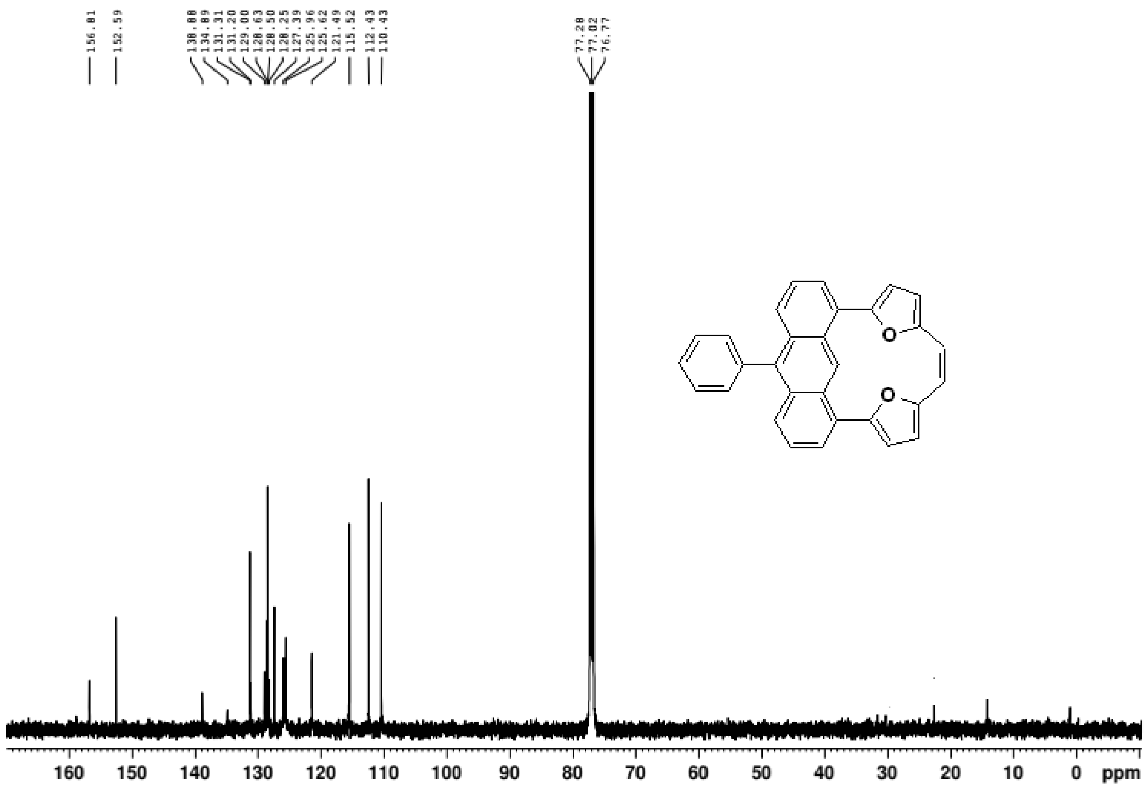

Fig. S19 ^{13}C NMR ($CDCl_3$, 126 MHz, 298 K) spectrum of **1b**.

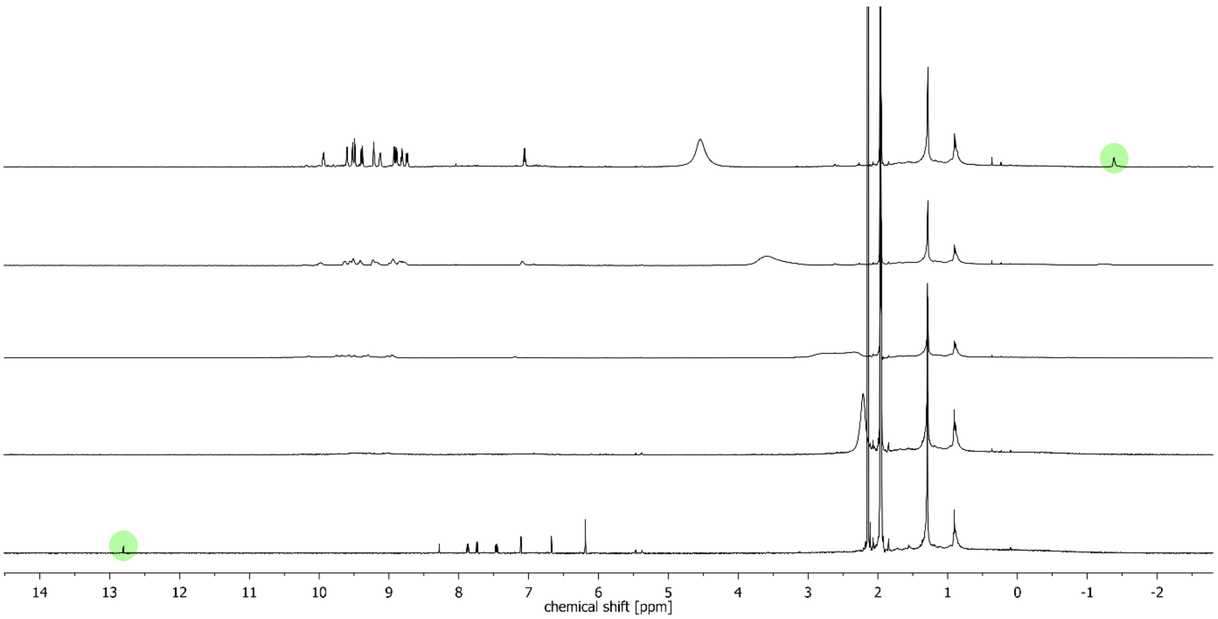

Fig. S20 ^{1}H NMR-monitored titration of **1a** with $NOSbF_6$. Acetonitrile-d_3, 600 MHz, 298 K. Position of the inner proton before (bottom) and after oxidation (top) is marked in green.

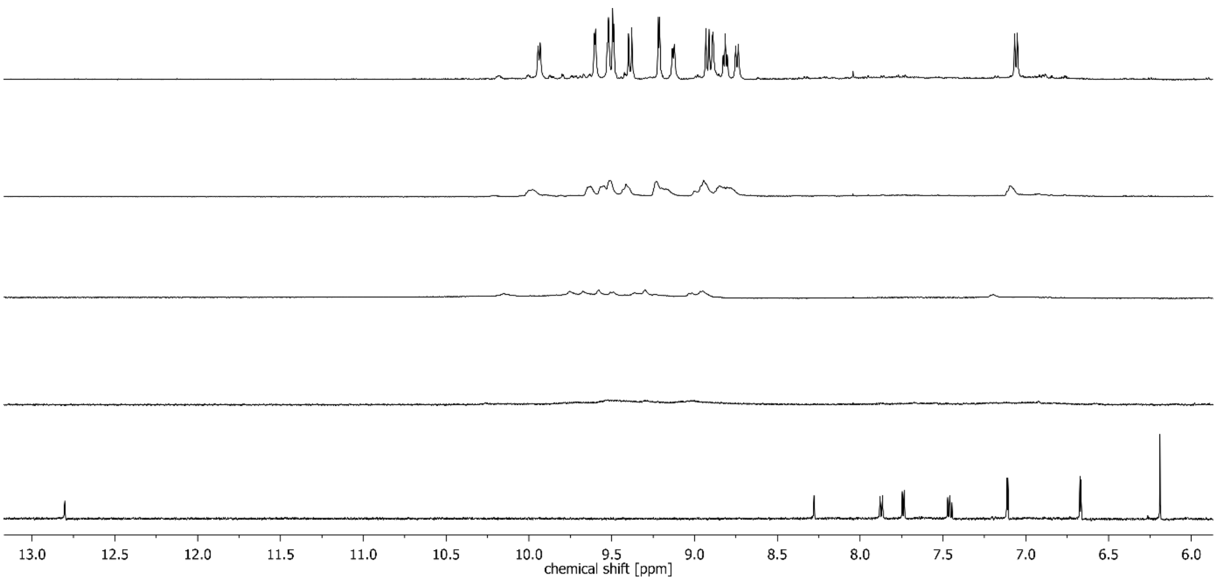

Fig. S21 Zoom on an aromatic region of the spectra during a ^{1}H NMR-monitored titration of **1a** with $NOSbF_6$. Acetonitrile-d_3, 600 MHz, 298 K.

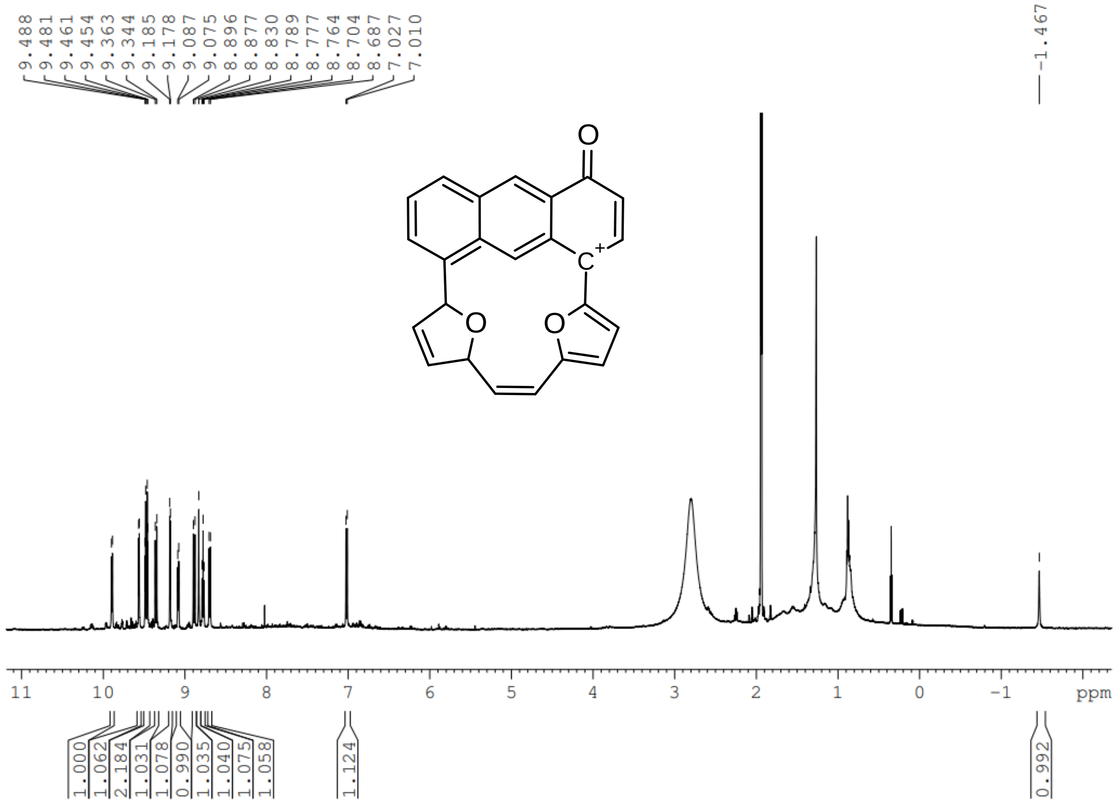

Fig. S22 ^{1}H NMR (CD_3CN, 600 MHz, 298 K) spectrum of **4a**.

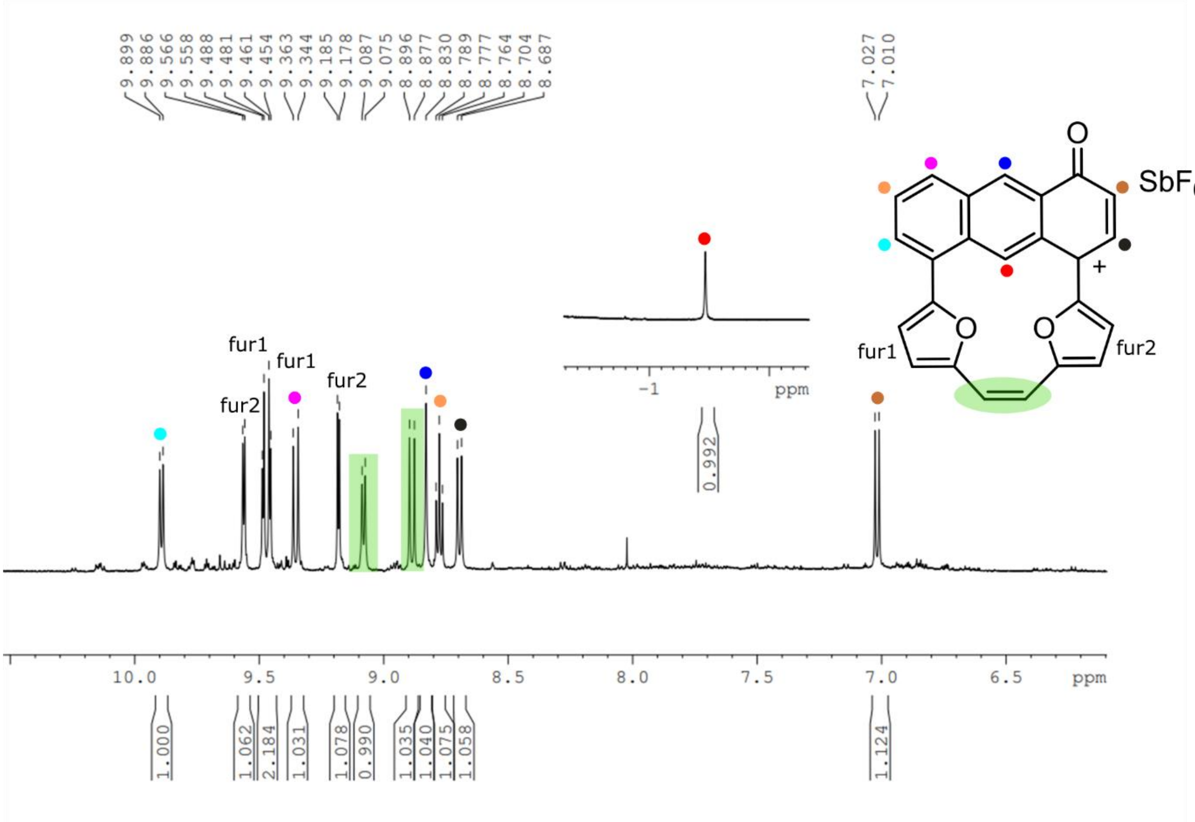

Fig. S23 ^{1}H NMR (zoom aromatic region) (CD_3CN, 600 MHz, 298 K) spectrum of **4a** together with partial assignment of peaks.

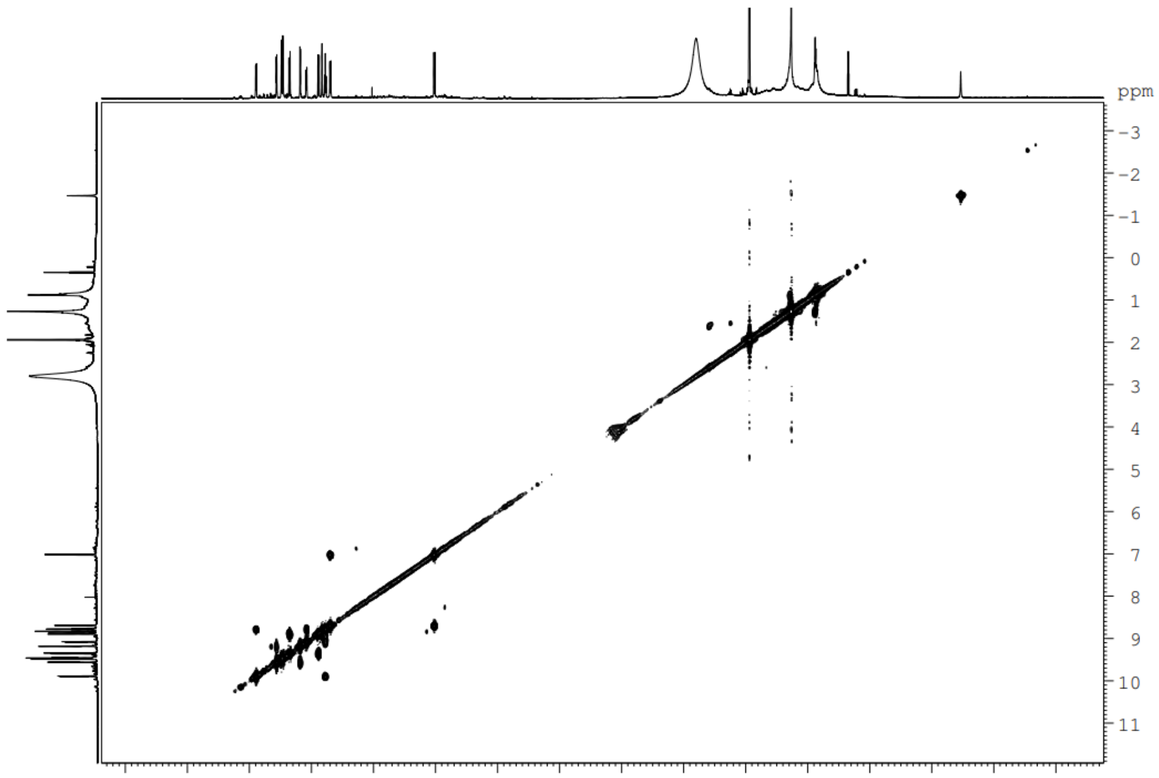

Fig. S24 ^{1}H-^{1}H COSY (CD_3CN, 600 MHz, 298 K) spectrum of **4a**.

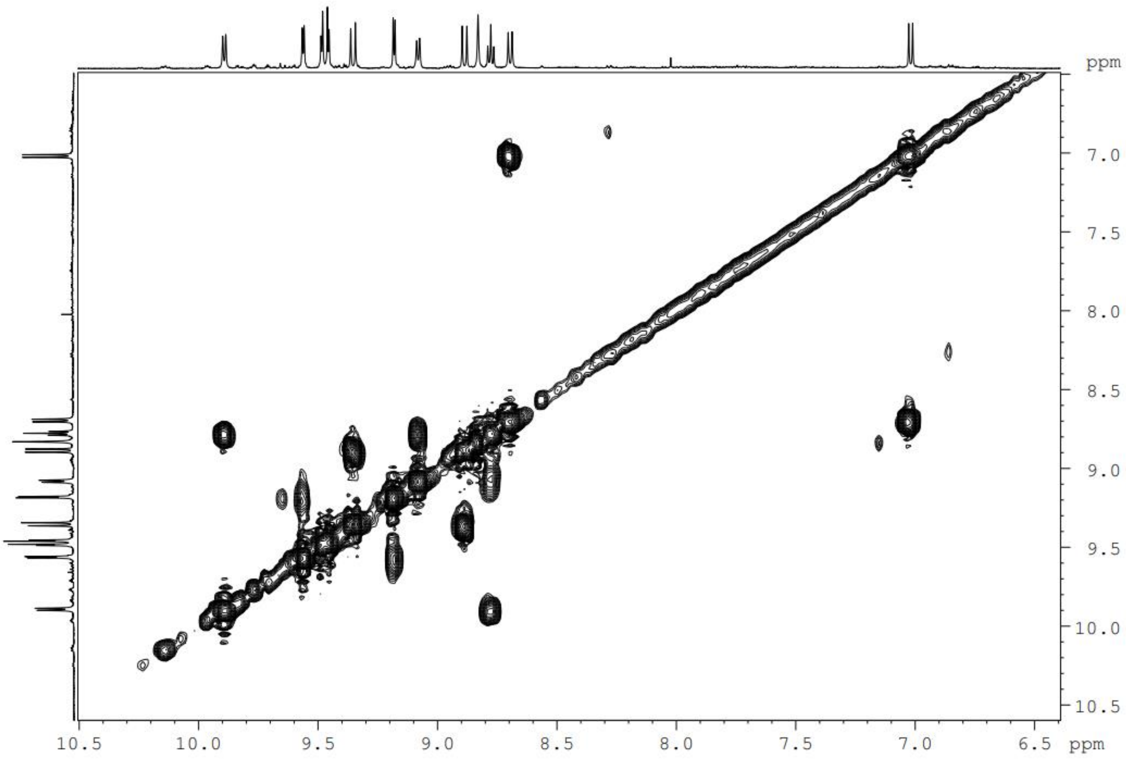

Fig. S25 ^{1}H-^{1}H COSY (zoom aromatic region) (CD_3CN, 600 MHz, 298 K) spectrum of **4a**.

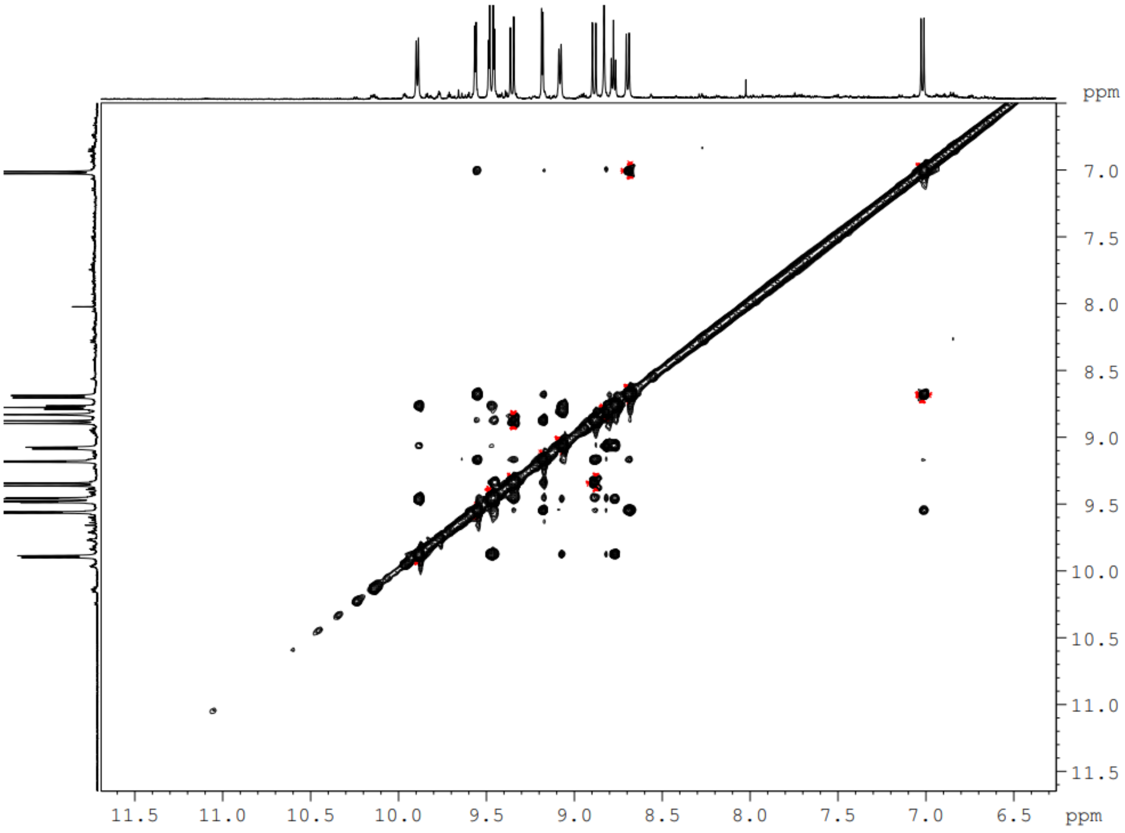

Fig. S26 ^{1}H-^{1}H NOESY (zoom aromatic region) (CD_3CN, 600 MHz, 298 K) spectrum of **4a**.

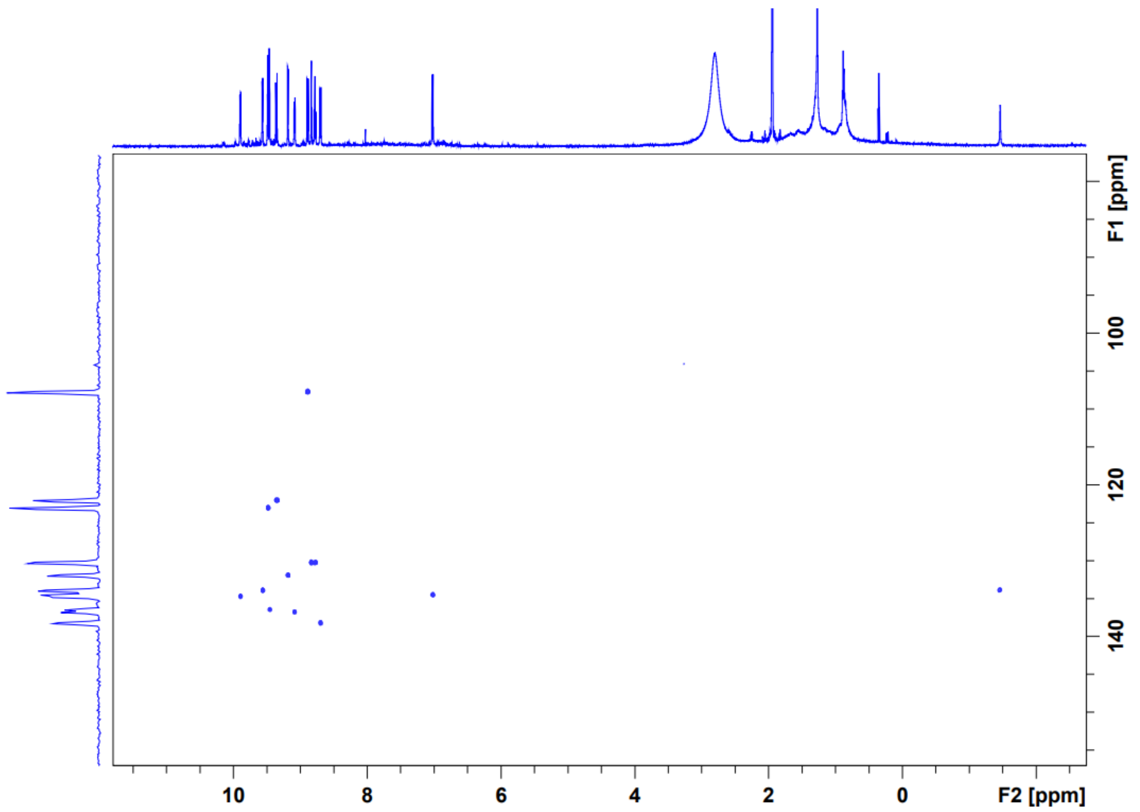

Fig. S27 ^{1}H-^{13}C HSQC NMR (CD_3CN, 600 MHz, 298 K) spectrum of **4a**.

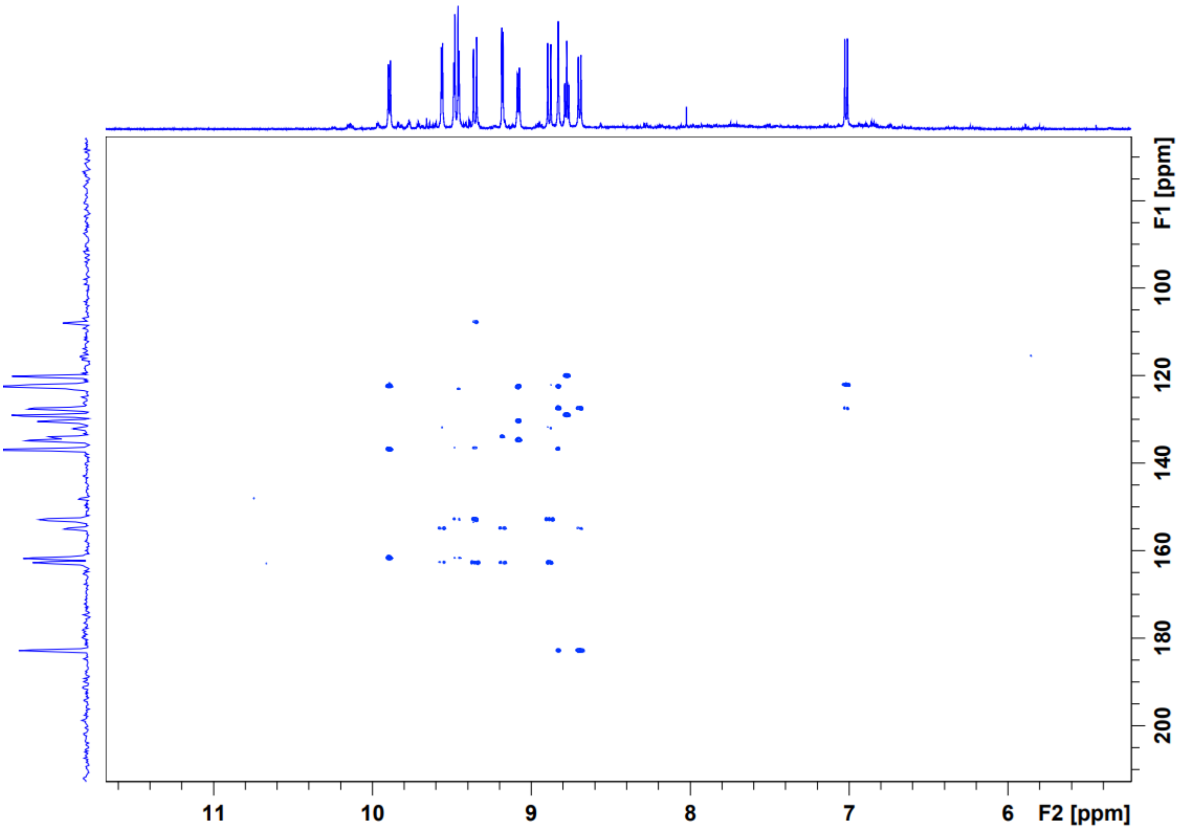

Fig. S28 ^{1}H-^{13}C HMBC NMR (CD_3CN, 600 MHz, 298 K) spectrum of **4a**.

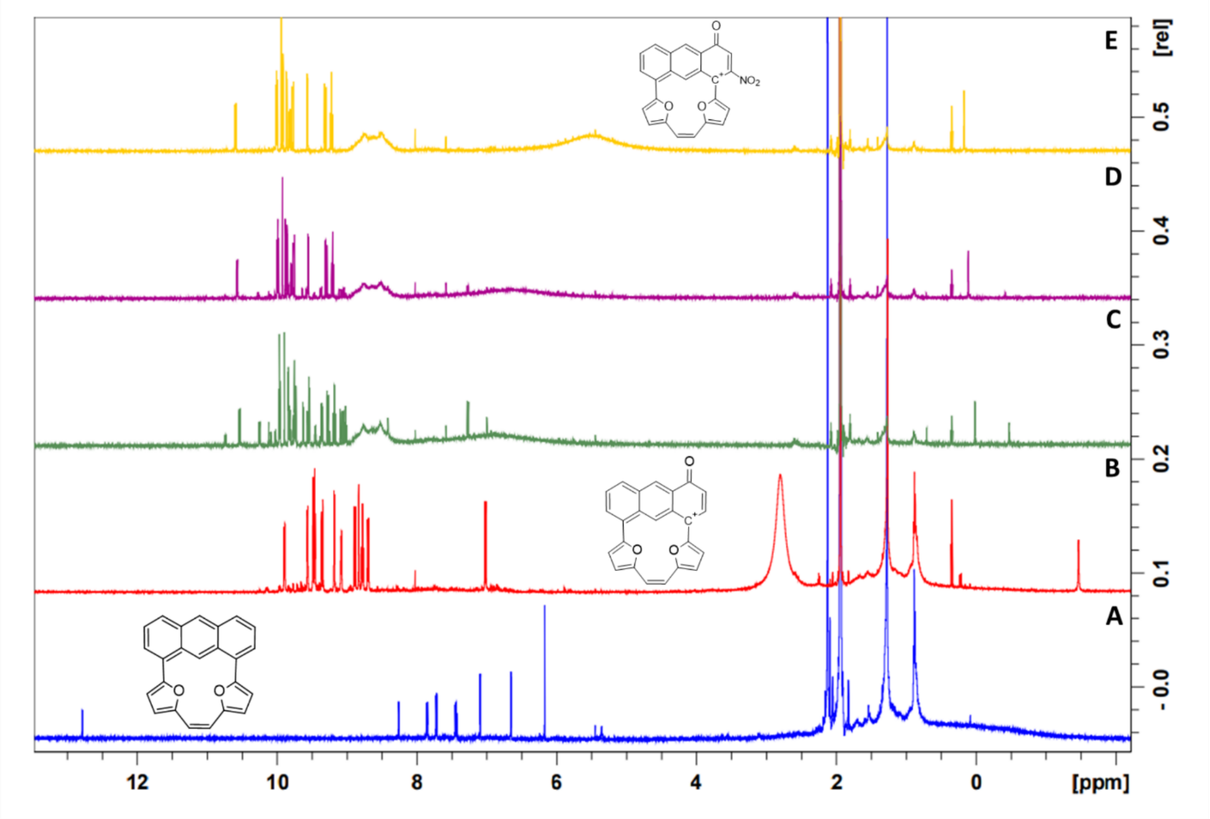

Fig. S29 ^{1}H NMR-monitored titration of **1a** with $NOSbF_6$ (Acetonitrile-d_3, 600 MHz, 298 K); A – without; B – 3.5 eq; C - 3.5 eq after 36 h; ; D - 3.5 eq after 48 h; E - 3.5 eq after 96 h.

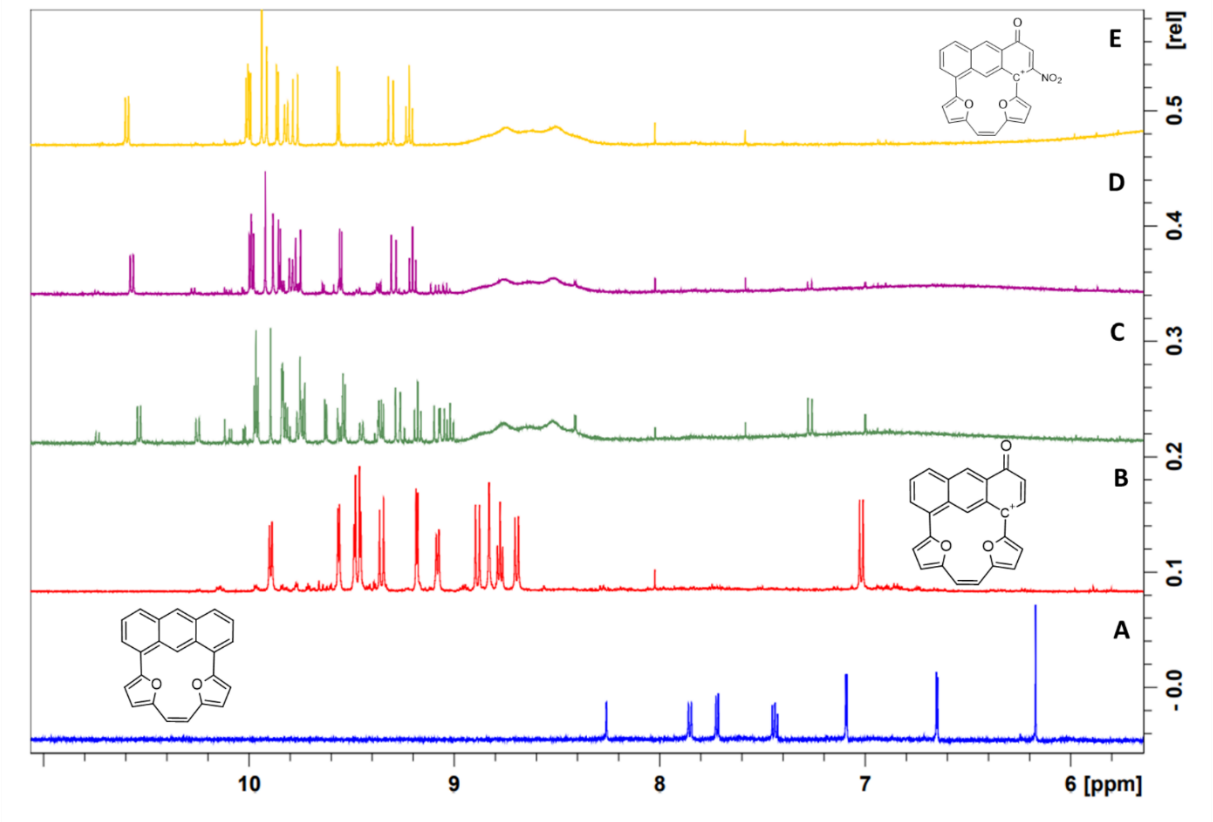

Fig. S30 Zoom on aromatic region of the spectra during ^{1}H NMR-monitored titration of **1a** with $NOSbF_6$ (Acetonitrile-d_3, 600 MHz, 298 K); A – without; B – 3.5 eq; C - 3.5 eq after 36 h; ; D - 3.5 eq after 48 h; E - 3.5 eq after 96 h.

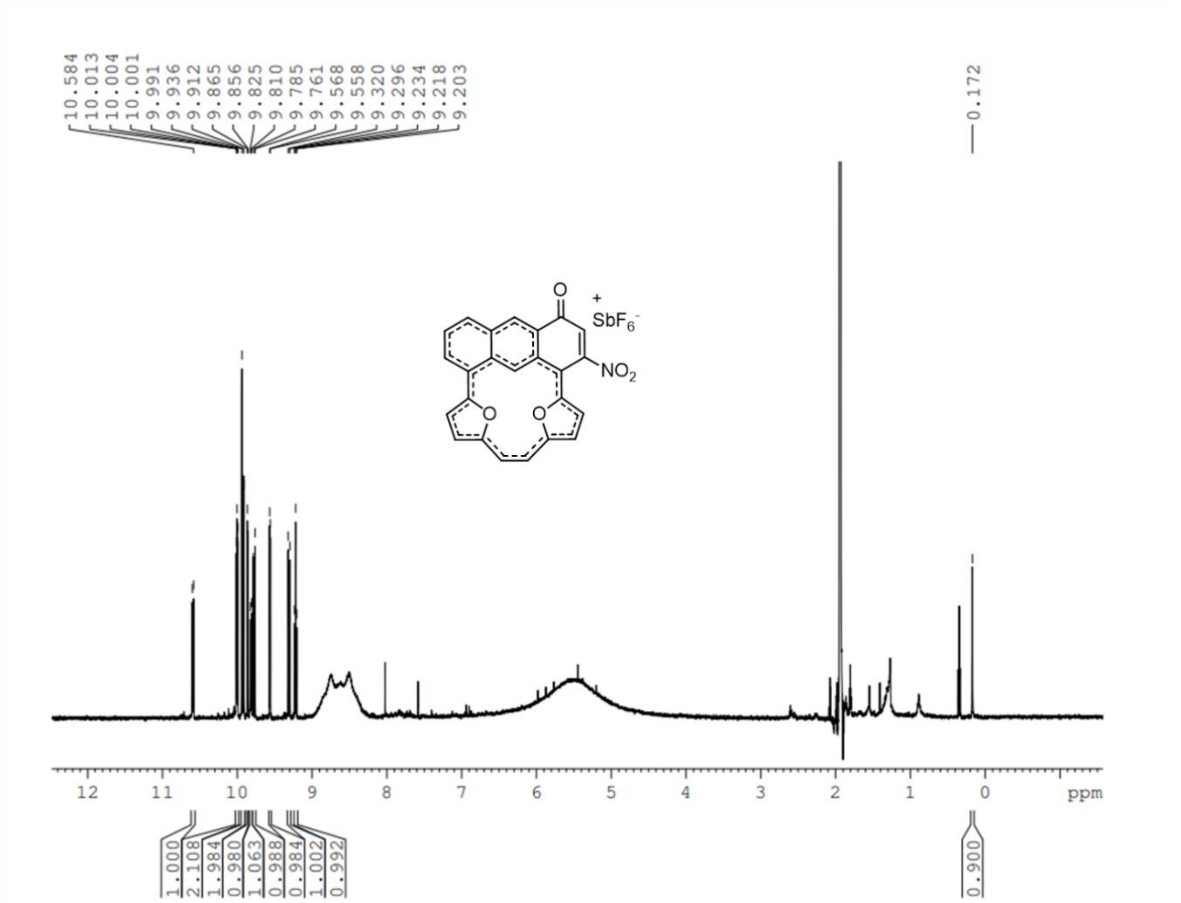

Fig. S31 ^{1}H NMR (CD_3CN, 600 MHz, 298 K) spectrum of **5**.

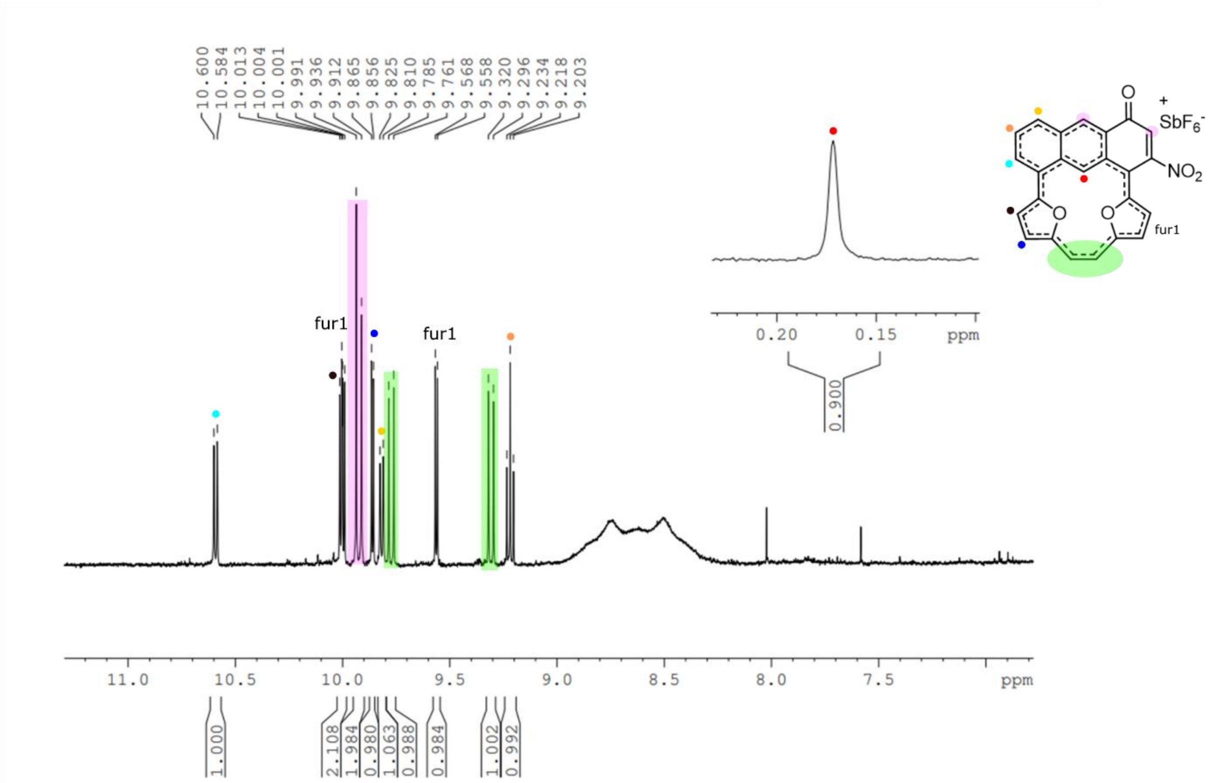

Fig. S32 ^{1}H NMR (zoom aromatic region) (CD_3CN, 600 MHz, 298 K) spectrum of **5** together with partial assignment of peaks.

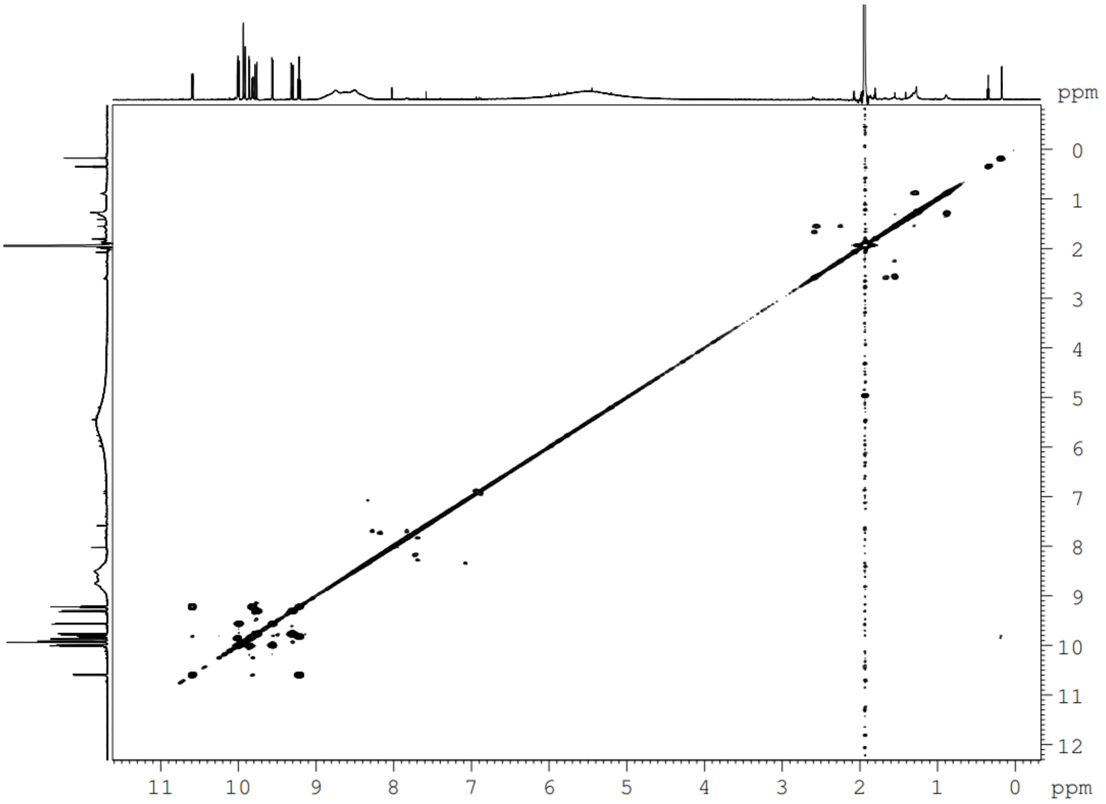

Fig. S33 ^{1}H-^{1}H COSY (CD_3CN, 600 MHz, 298 K) spectrum of **5**.

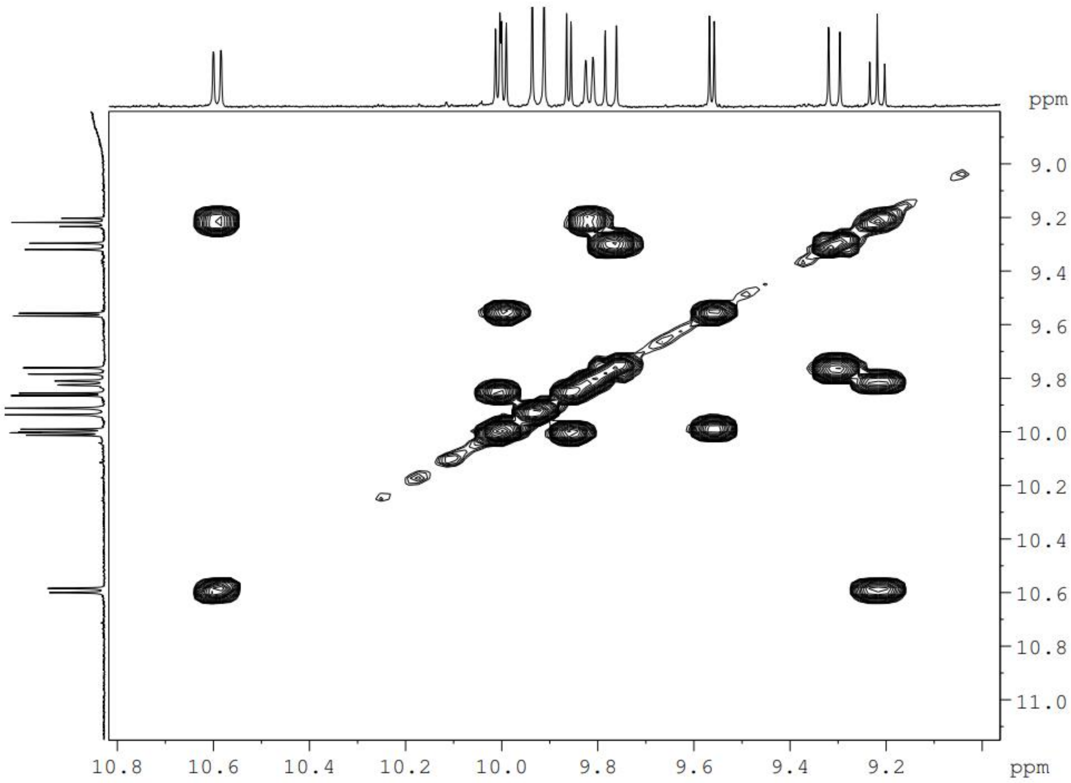

Fig. S34 ^{1}H-^{1}H COSY (zoom aromatic region) (CD_3CN, 600 MHz, 298 K) spectrum of **5**.

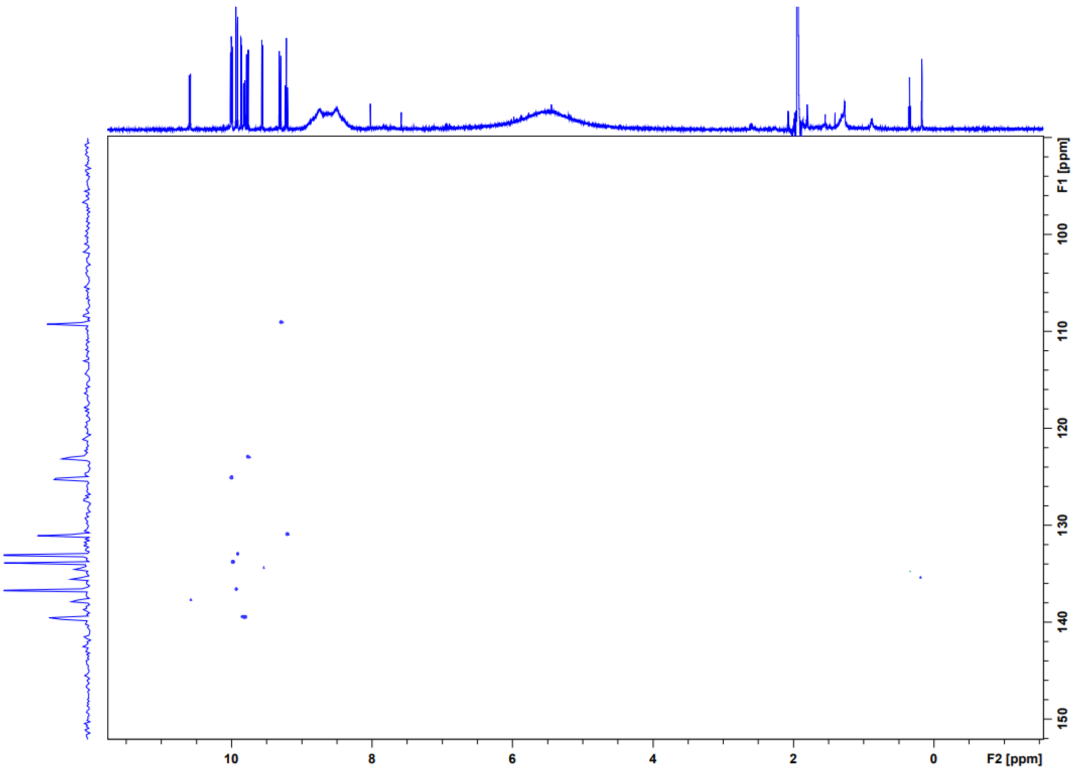

Fig. S35 ^{1}H-^{13}C HSQC (CD_3CN, 600 MHz, 298 K) spectrum of **5**.

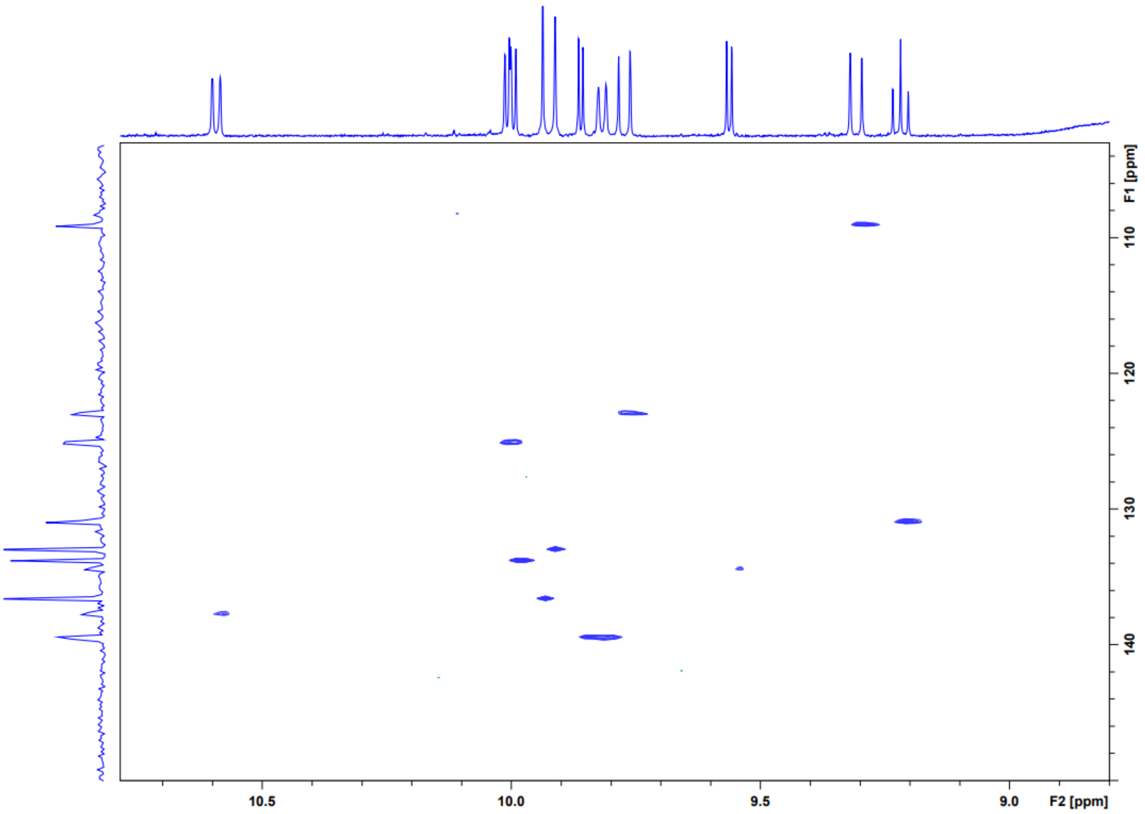

Fig. S36 ^{1}H-^{13}C HSQC (zoom aromatic region) (CD_3CN, 600 MHz, 298 K) spectrum of **5**.

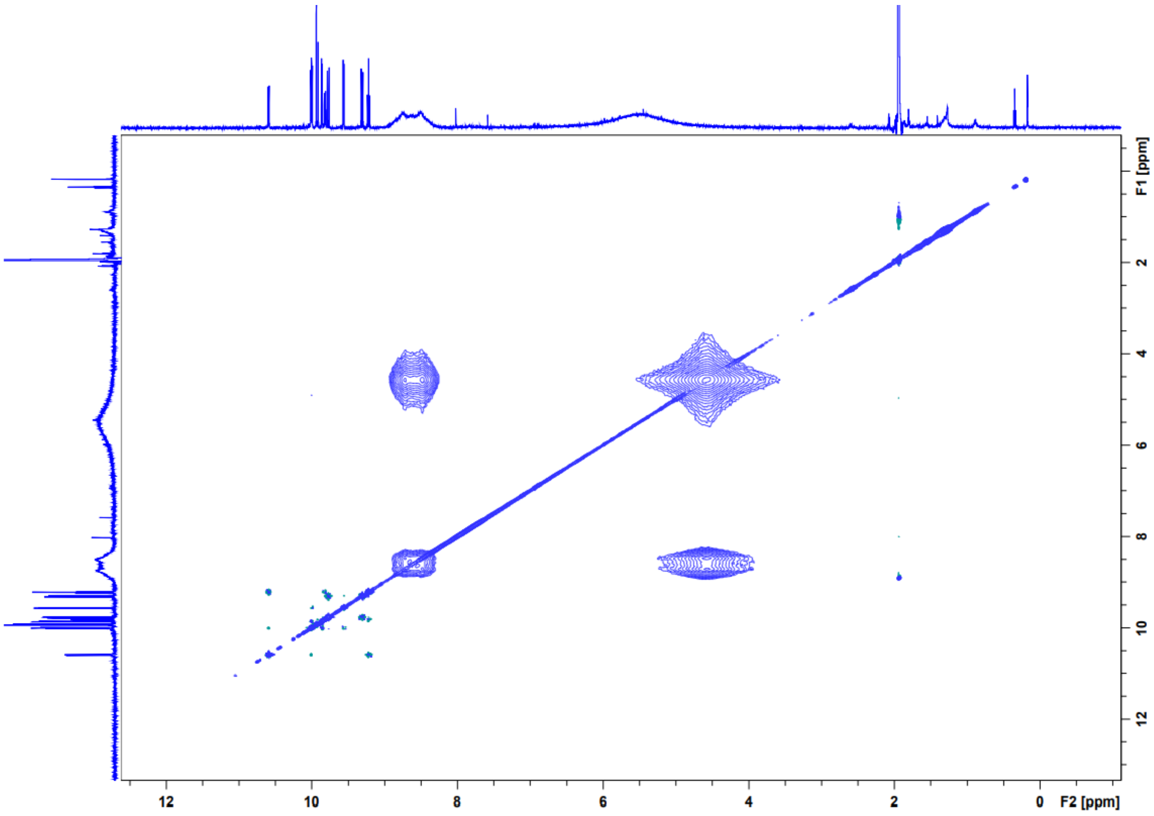

Fig. S37 ^{1}H-^{1}H NOESY (CD_3CN, 600 MHz, 298 K) spectrum of **5**.

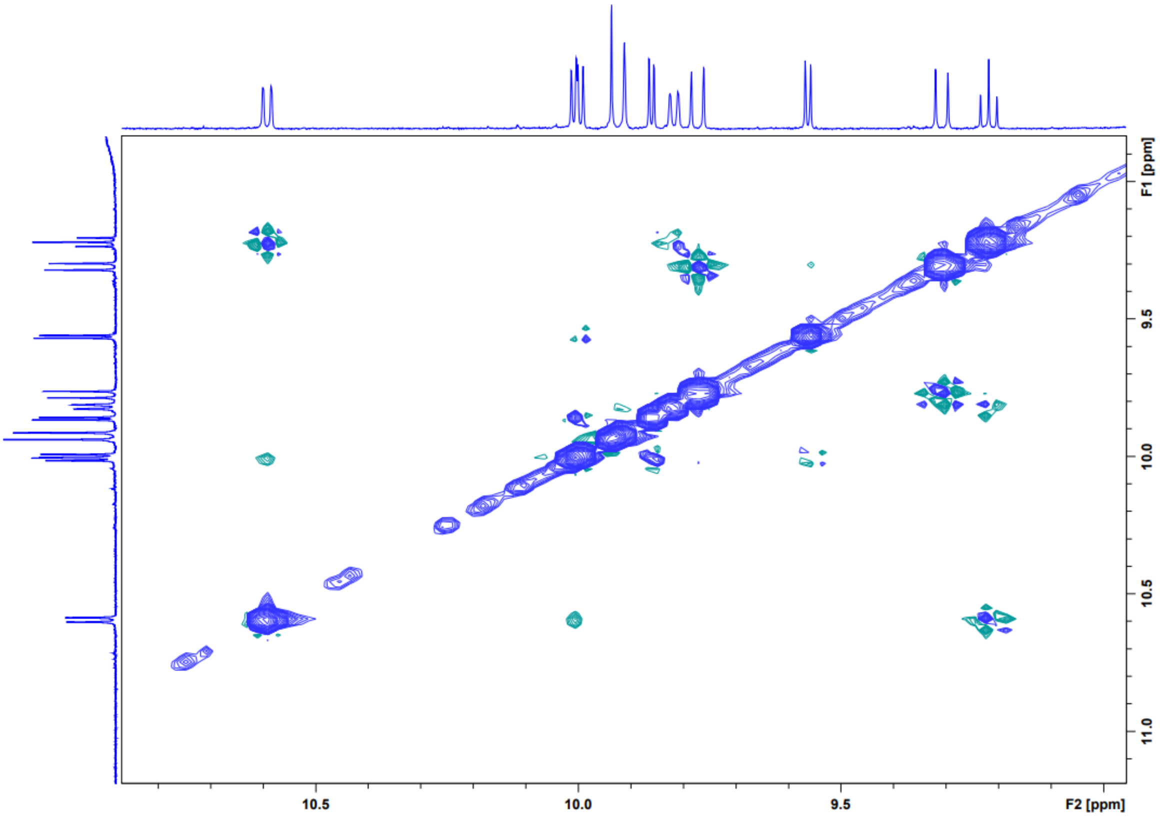

Fig. S38 ^{1}H-^{1}H NOESY (zoom aromatic region) (CD_3CN, 600 MHz, 298 K) spectrum of **5**.

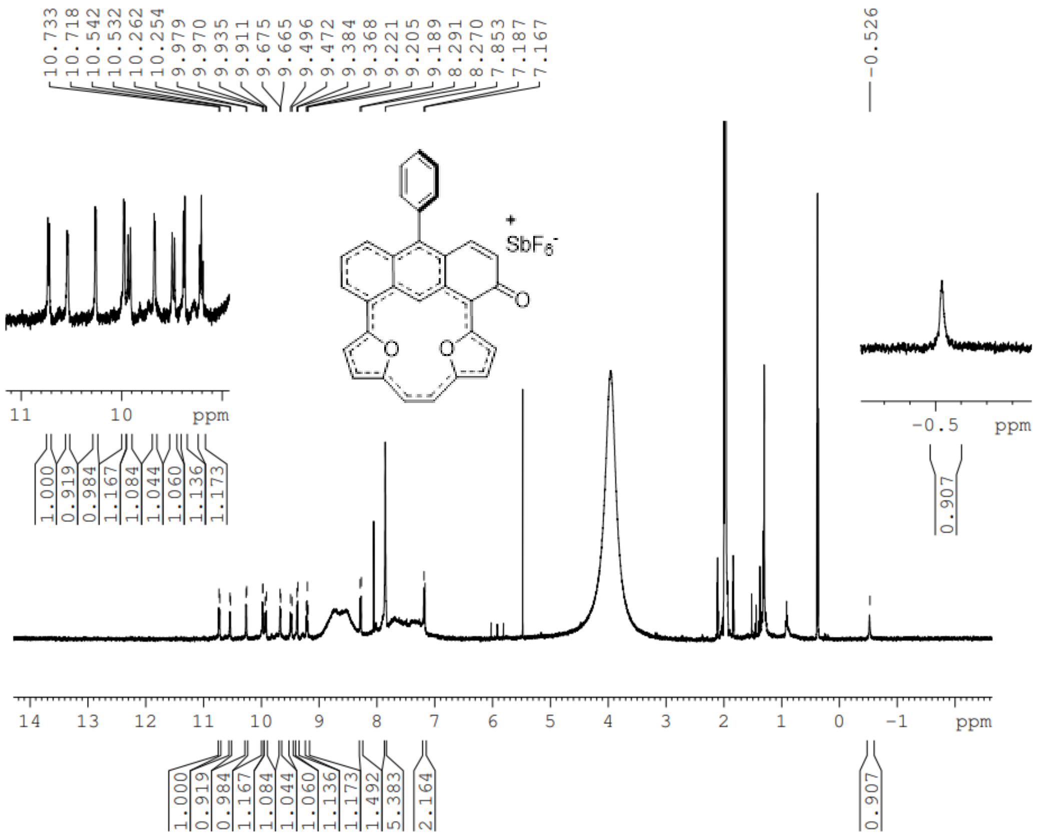

Fig. S39 ^{1}H NMR (CD_3CN, 600 MHz, 298 K) spectrum of **4b**

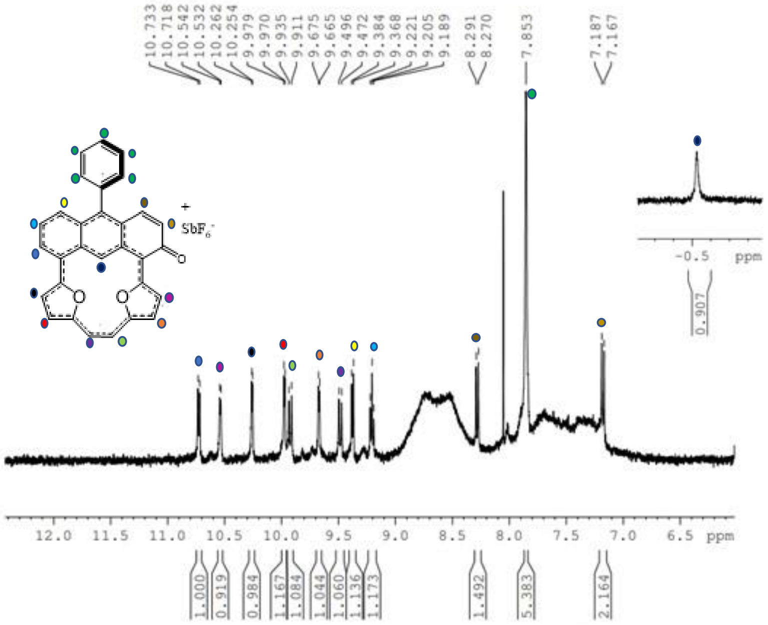

Fig. S40 ^{1}H NMR (zoom aromatic region) (CD_3CN, 600 MHz, 298 K) spectrum of **4b**

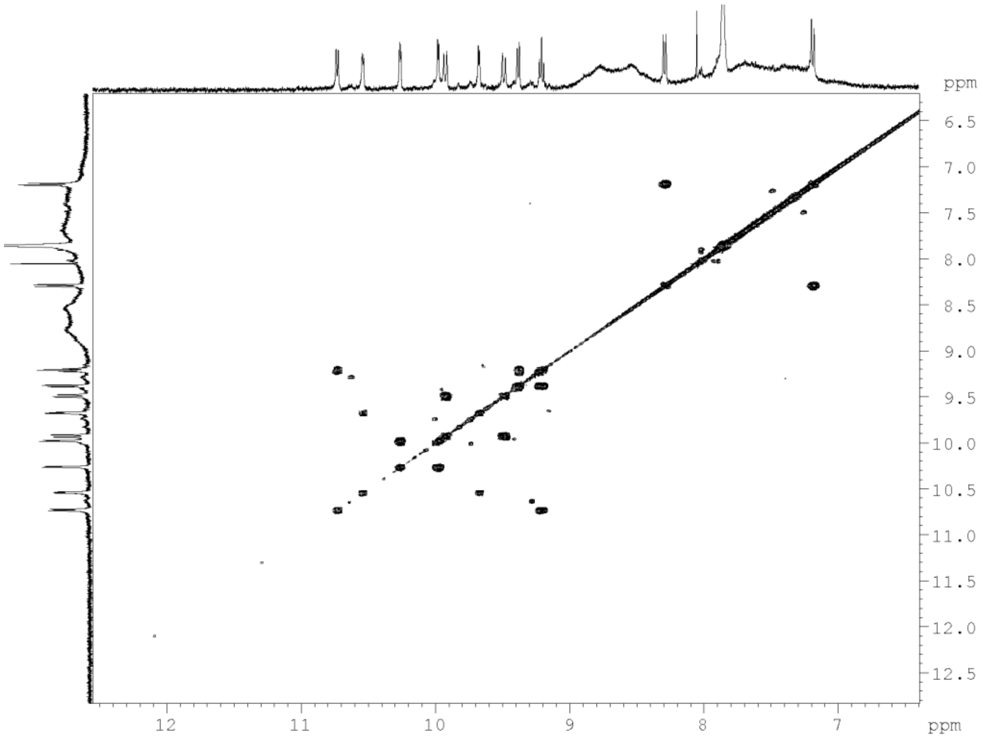

Fig. S41 ^{1}H-^{1}H COSY (CD_3CN, 600 MHz, 298 K) spectrum of **4b**

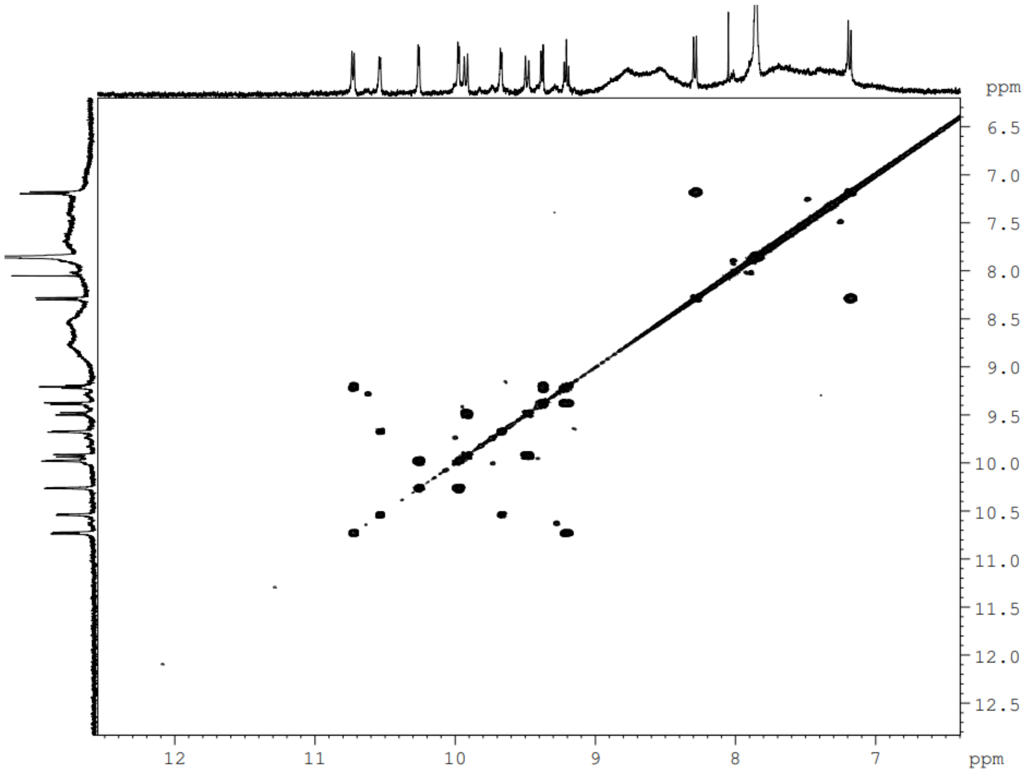

Fig. S42 ^{1}H-^{1}H COSY (zoom aromatic region) (CD_3CN, 600 MHz, 298 K) spectrum of **4b**

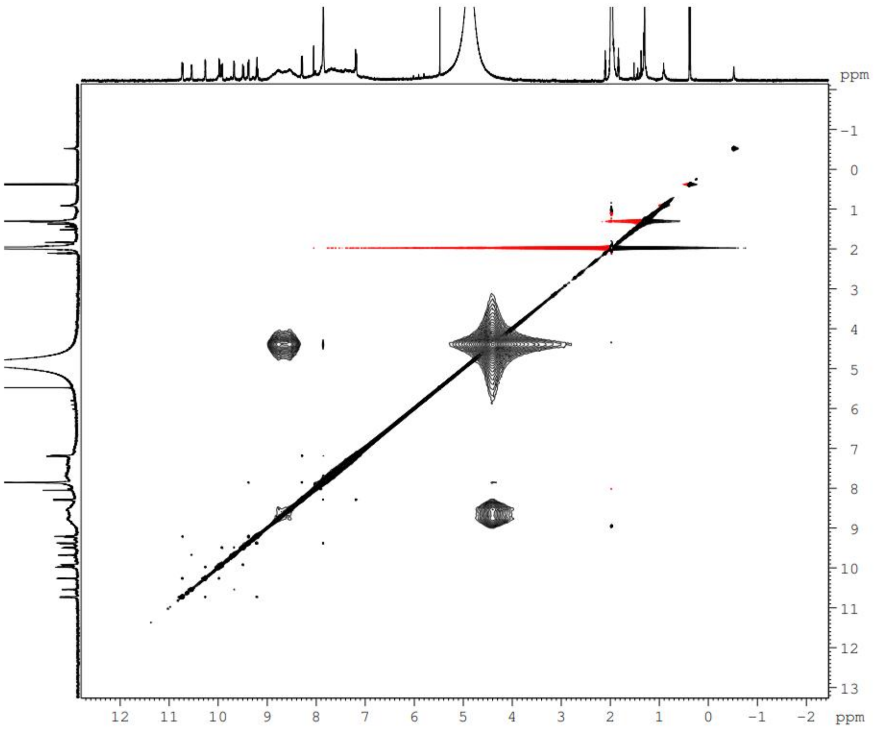

Fig. S43 ^{1}H-^{1}H NOESY (CD_3CN, 600 MHz, 298 K) spectrum of **4b**

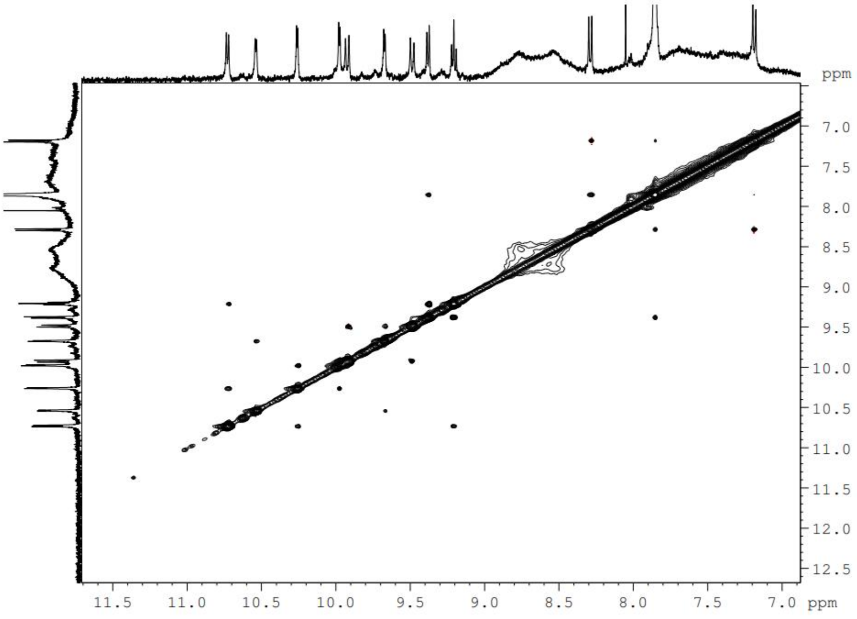

Fig. S44 ^{1}H-^{1}H NOESY (zoom aromatic region) (CD_3CN, 600 MHz, 298 K) spectrum of ***4b***

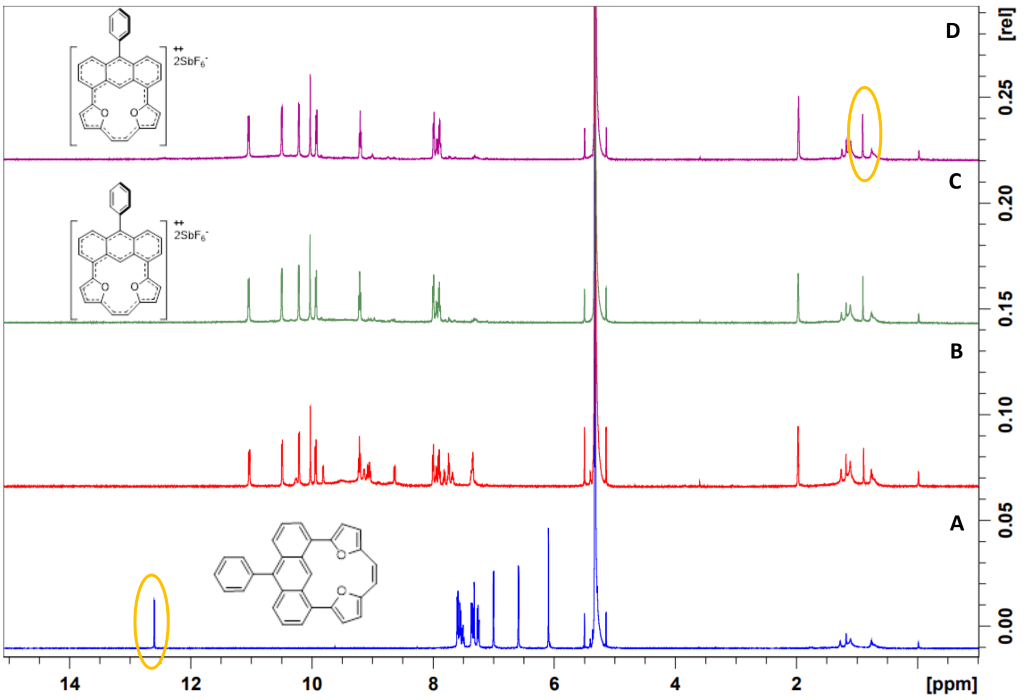

Fig. S45 ^{1}H NMR-monitored titration of **1b** with $NOSbF_6$ (CD_2Cl_2, 600 MHz, 193 K); A – without (blue); B – 2eq (red); C - 3 eq (green); ; D - 4 eq (purple)

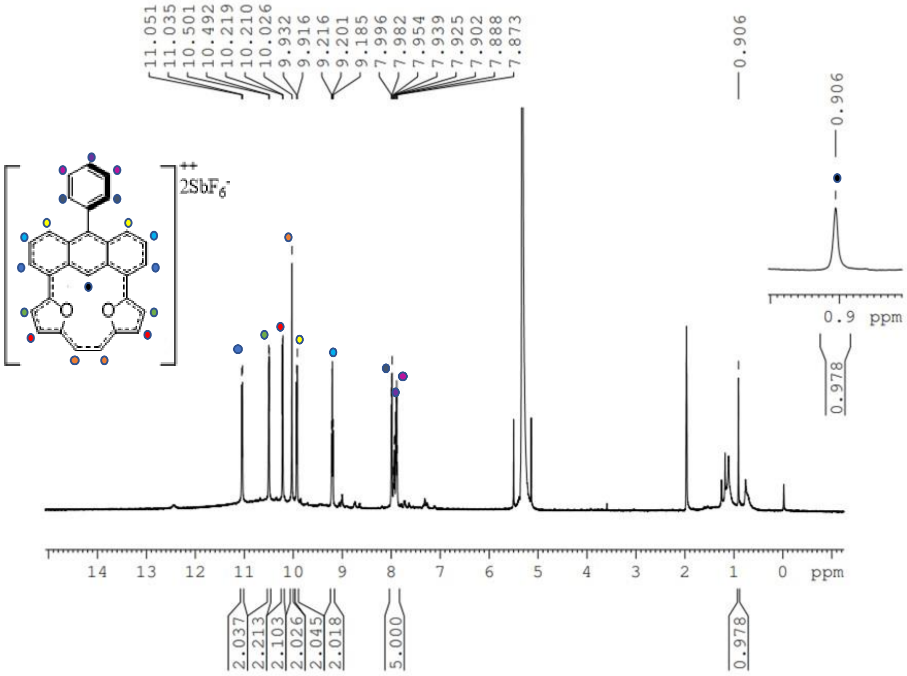

Fig. S46 ^{1}H NMR of $\mathbf{1b}^{2+}$ with $NOSbF_6$ (CD_2Cl_2, 600 MHz, 193 K)

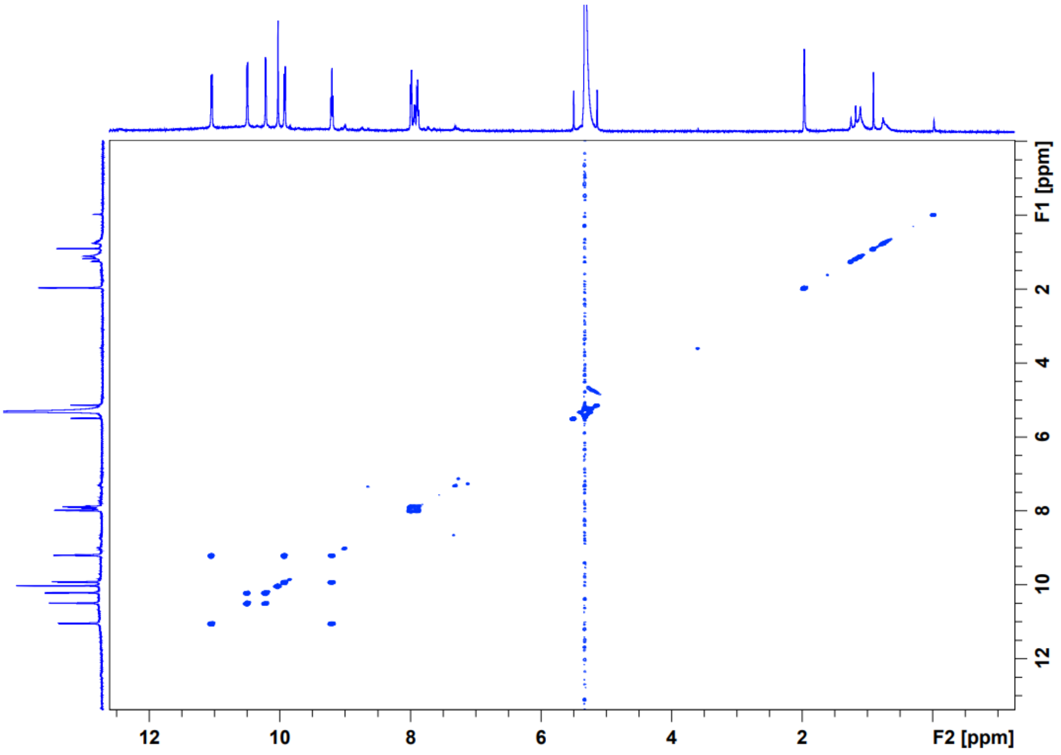

Fig. **S46** ^{1}H-^{1}H COSY (CD_2Cl_2, 600 MHz, 193 K) spectrum of $\mathbf{1b}^{2+}$

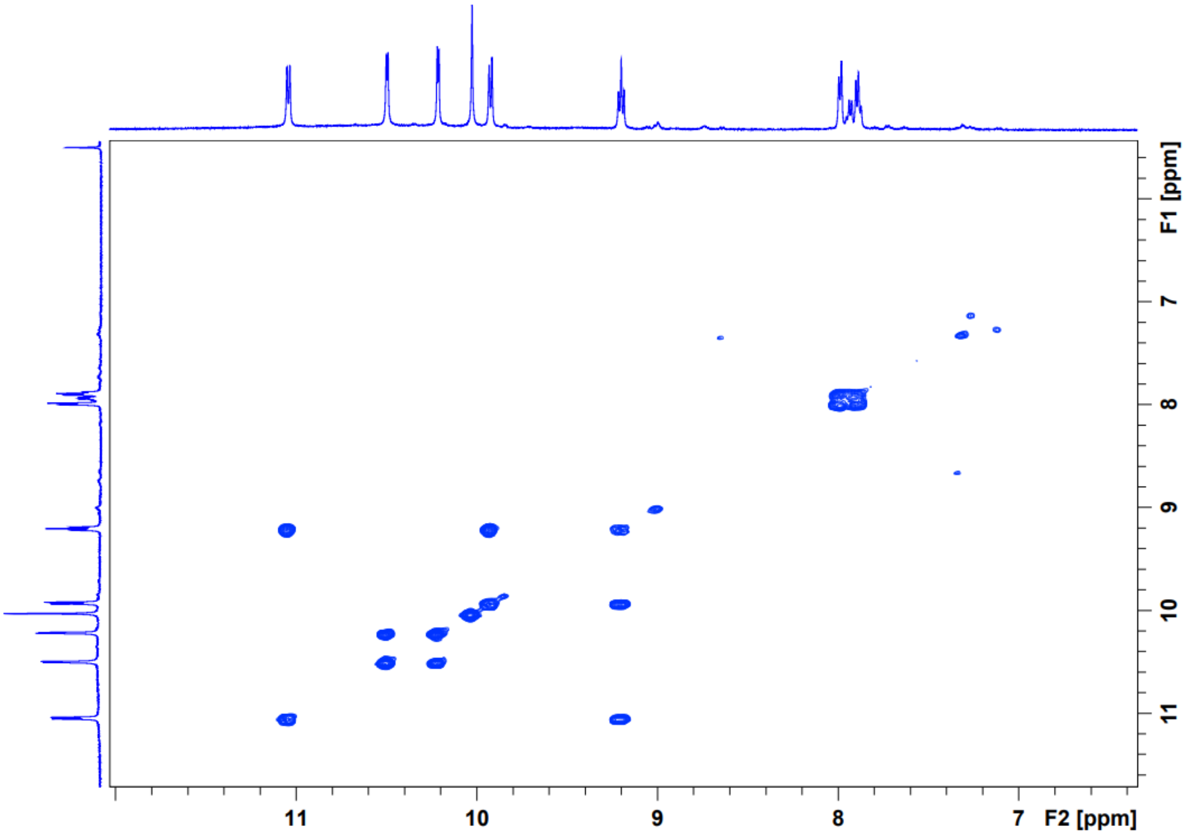

Fig. S47 ^{1}H-^{1}H COSY (zoom aromatic region) (CD_2Cl_2, 600 MHz, 193 K) spectrum of $\mathbf{1b}^{2+}$

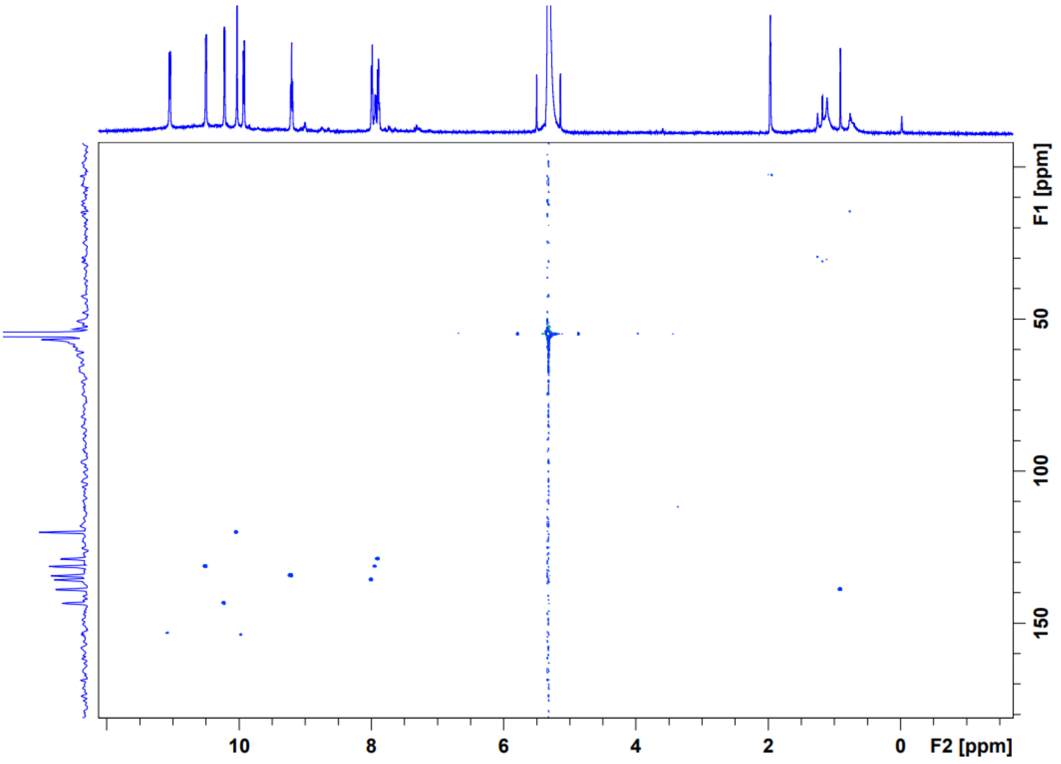

Fig. **S48** ^{1}H-^{13}C HSQC (CD_2Cl_2, 600 MHz, 193 K) spectrum of $\mathbf{1b^{2+}}$

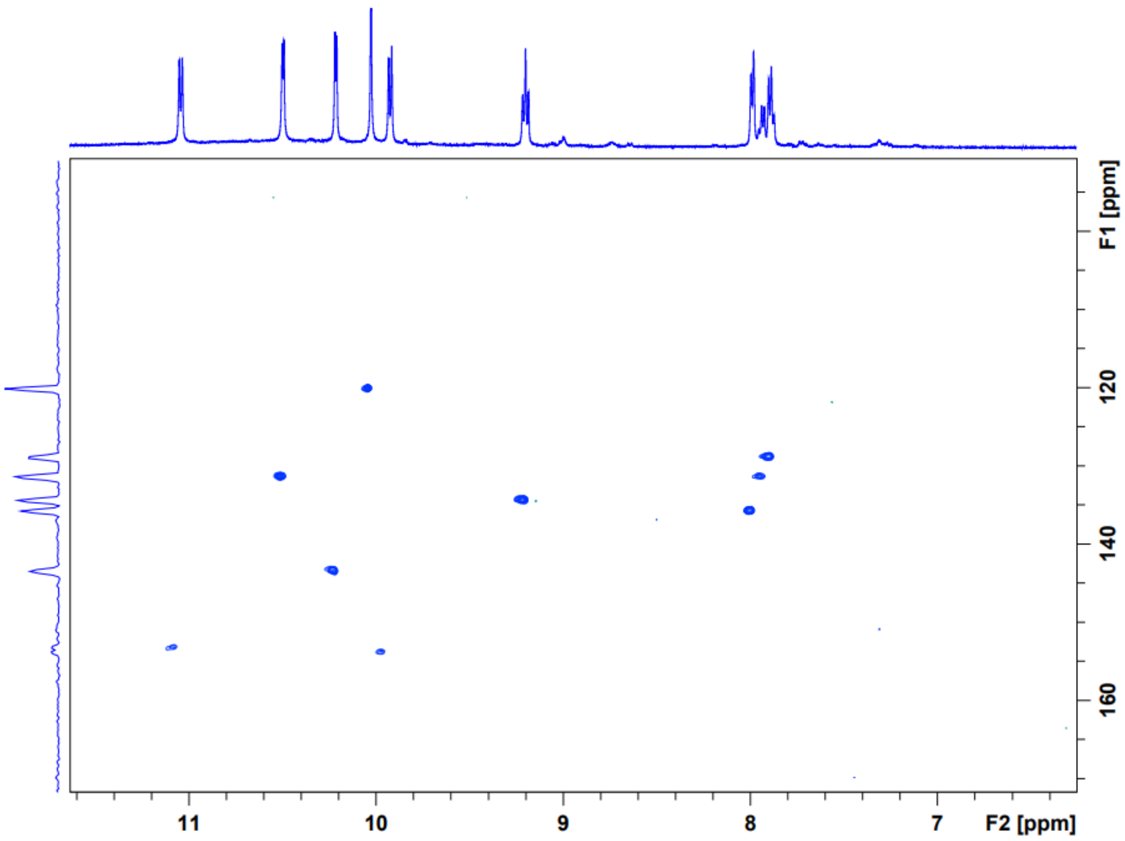

Fig. **S49** ^{1}H-^{13}C HSQC (zoom aromatic region) (CD_2Cl_2, 600 MHz, 193 K) spectrum of **1b^{2+}**

4. UV-VIS spectra

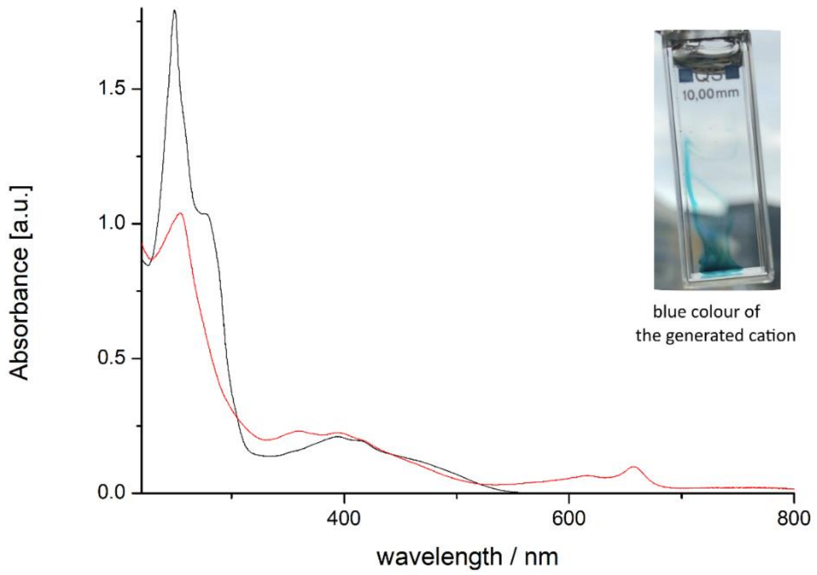

Fig. S50 UV-Vis spectra of the neutral macrocycle **1a** (black line) and the cation **4a** (red line), in acetonitrile at rt.

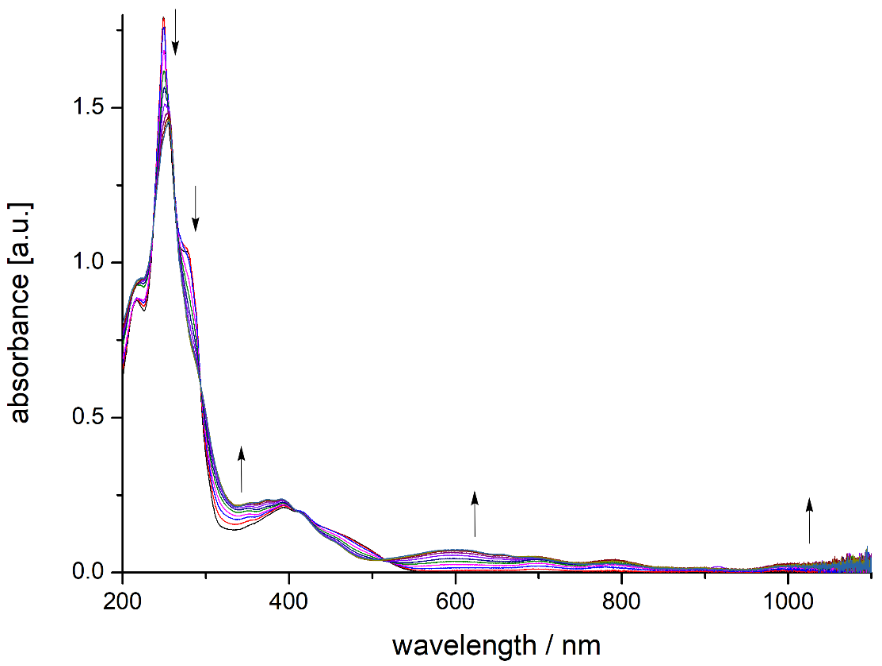

Fig. S51 UV-Vis spectra recorded during titration of **1a** with $NOSbF_6$, from 0 to 2 equiv., in acetonitrile at rt.

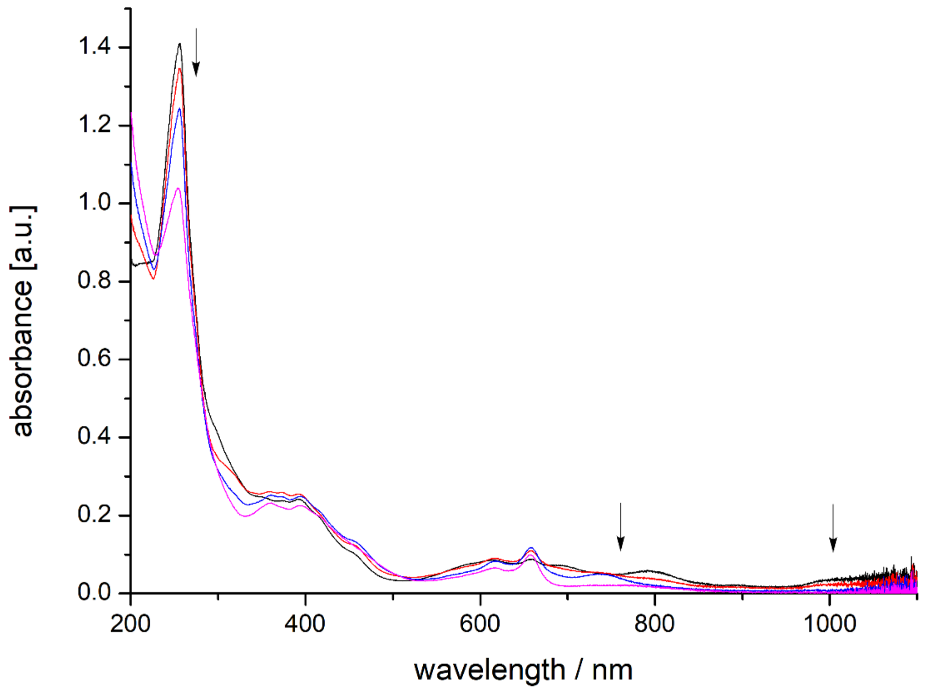

Fig. S52 UV-Vis spectra recorded during titration of **1a** with $NOSbF_6$, from 2 (black) to 5 (magenta) equiv., in acetonitrile at rt.

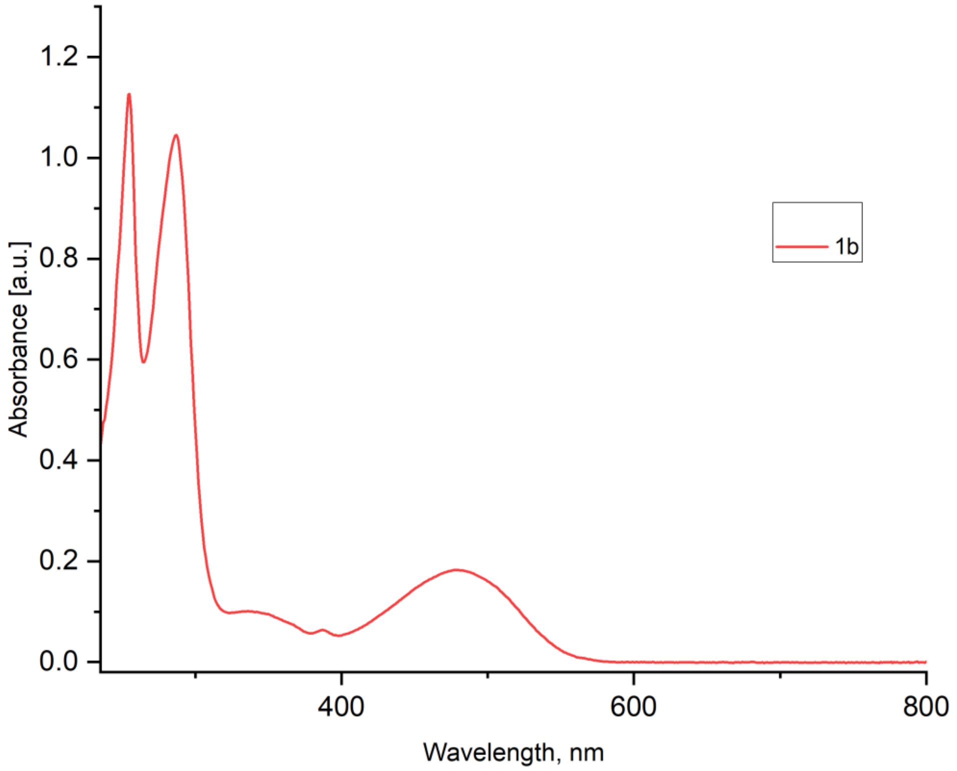

Fig. S54 UV-Vis spectrum of the neutral macrocycle **1b** in CH_2Cl_2 at 298 K.

5. ESI-MS spectra

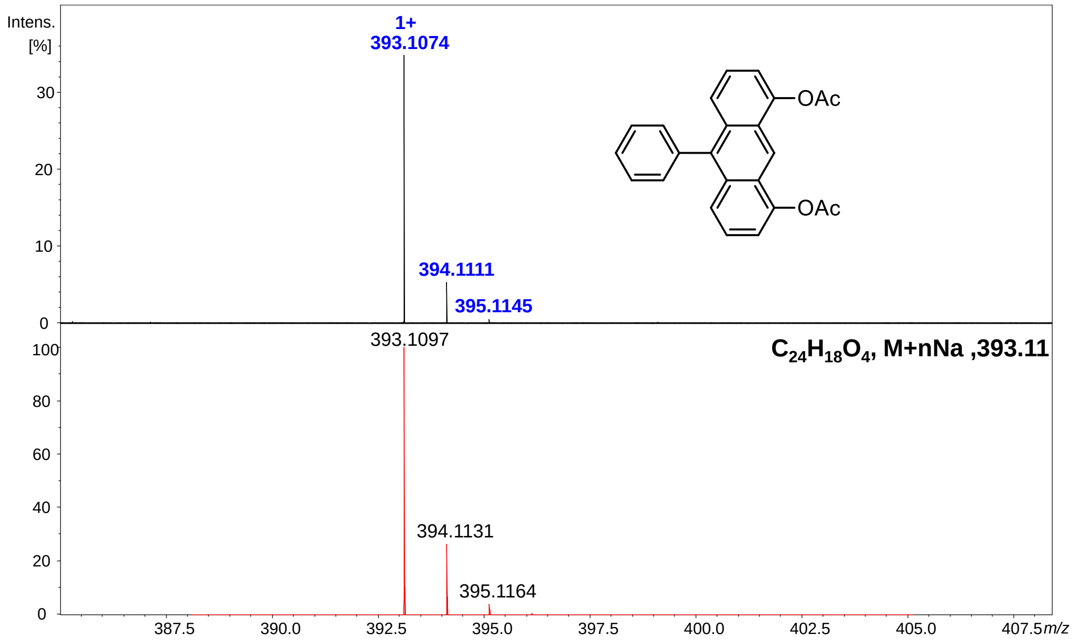

Fig. S55 ESI-MS spectrum of **S2** with simulated spectrum (bottom panel - simulated isotopic patterns for molecular formula of investigated compounds).

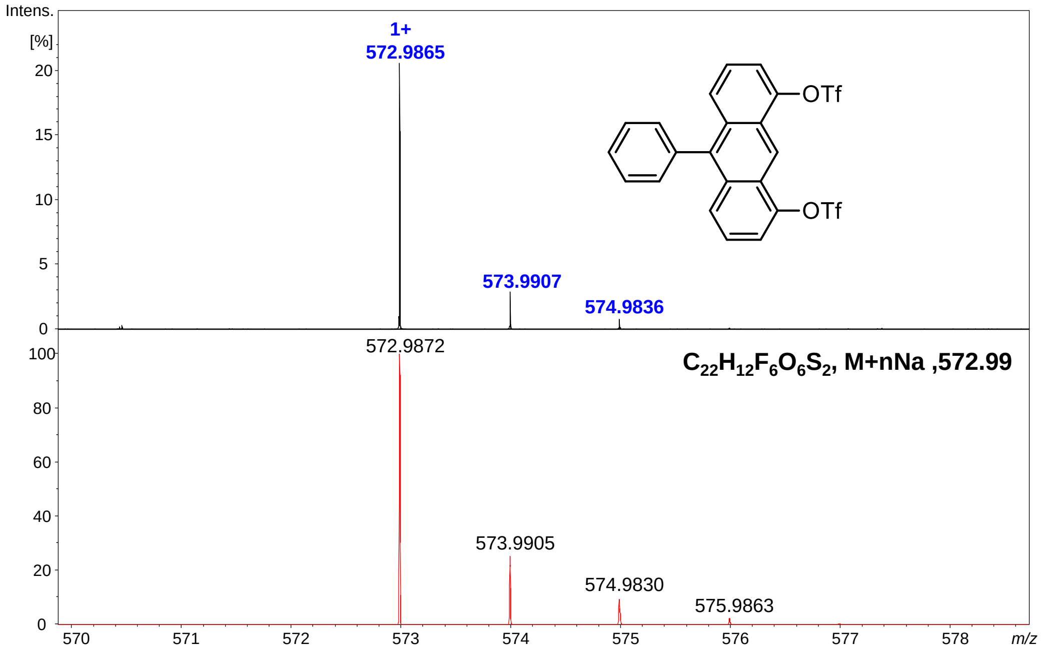

Fig. S56 ESI-MS spectrum of **2b** with simulated spectrum (bottom panel - simulated isotopic patterns for molecular formula of investigated compounds).

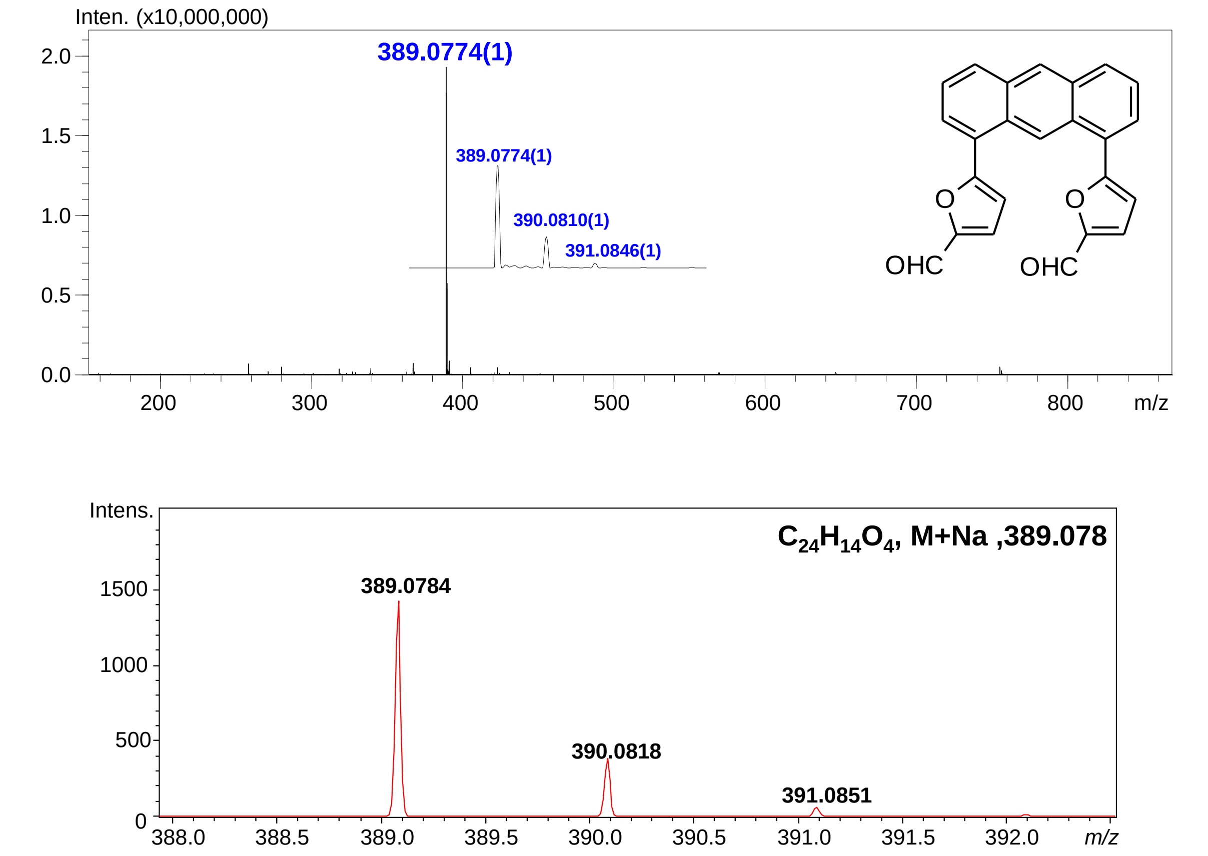

Fig. S57 ESI-MS spectrum of **3a** with simulated spectrum (bottom panel - simulated isotopic patterns for molecular formula of investigated compounds).

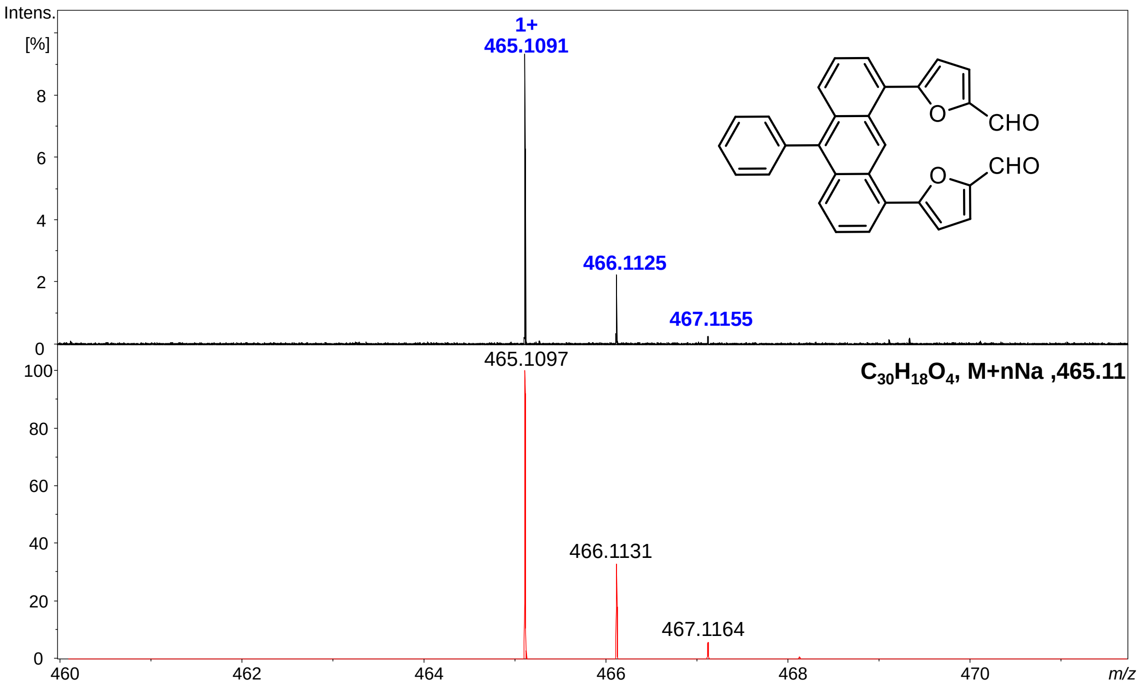

Fig. S58 ESI-MS spectrum of **3b** with simulated spectrum (bottom panel - simulated isotopic patterns for molecular formula of investigated compounds).

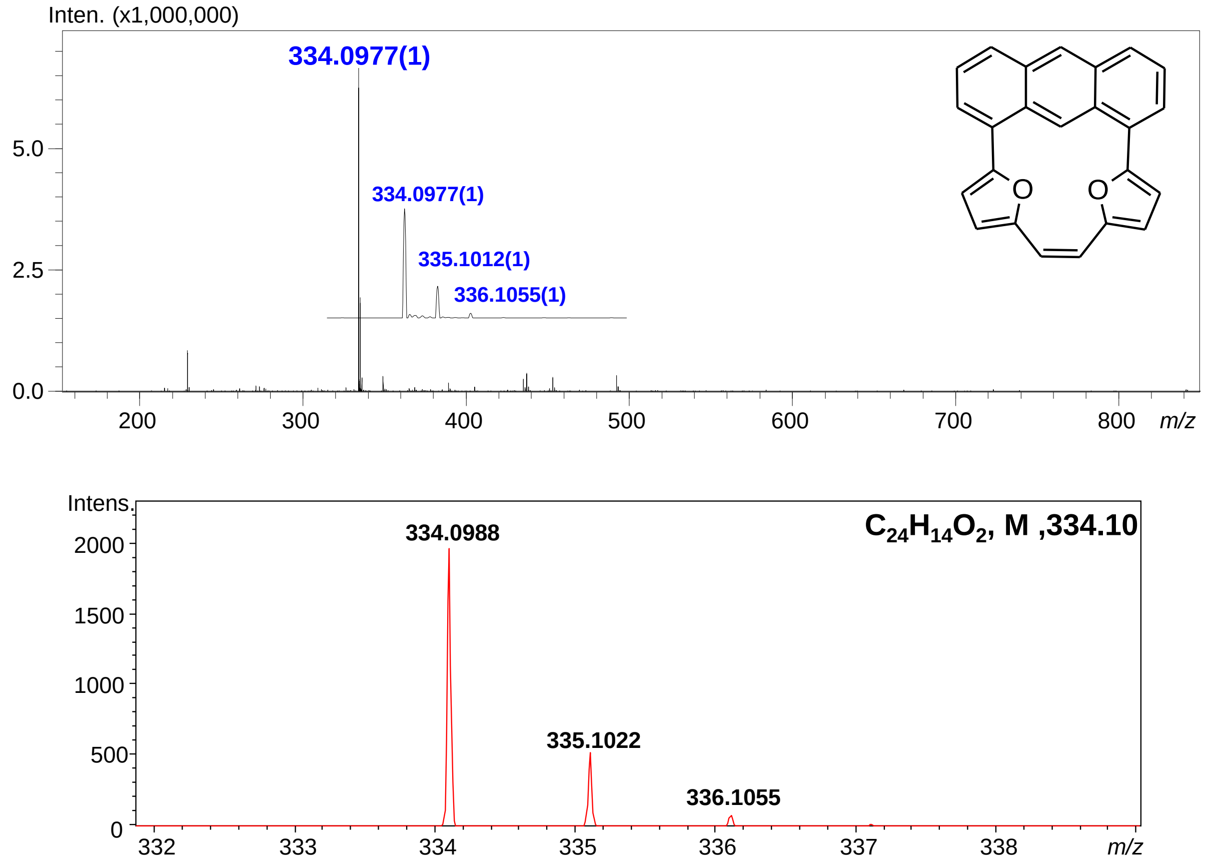

Fig. S59 ESI-MS spectrum of **1a** with simulated spectrum (bottom panel - simulated isotopic patterns for molecular formula of investigated compounds).

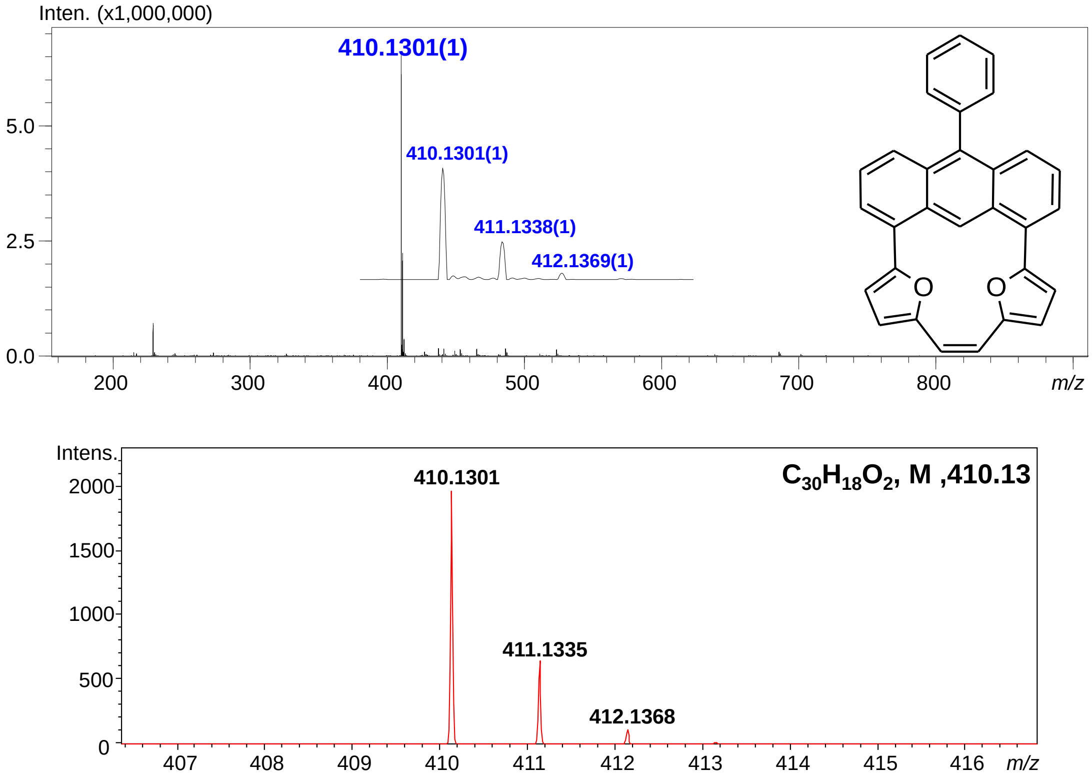

Fig. S60 ESI-MS spectrum of **1b** with simulated spectrum (bottom panel - simulated isotopic patterns for molecular formula of investigated compounds).

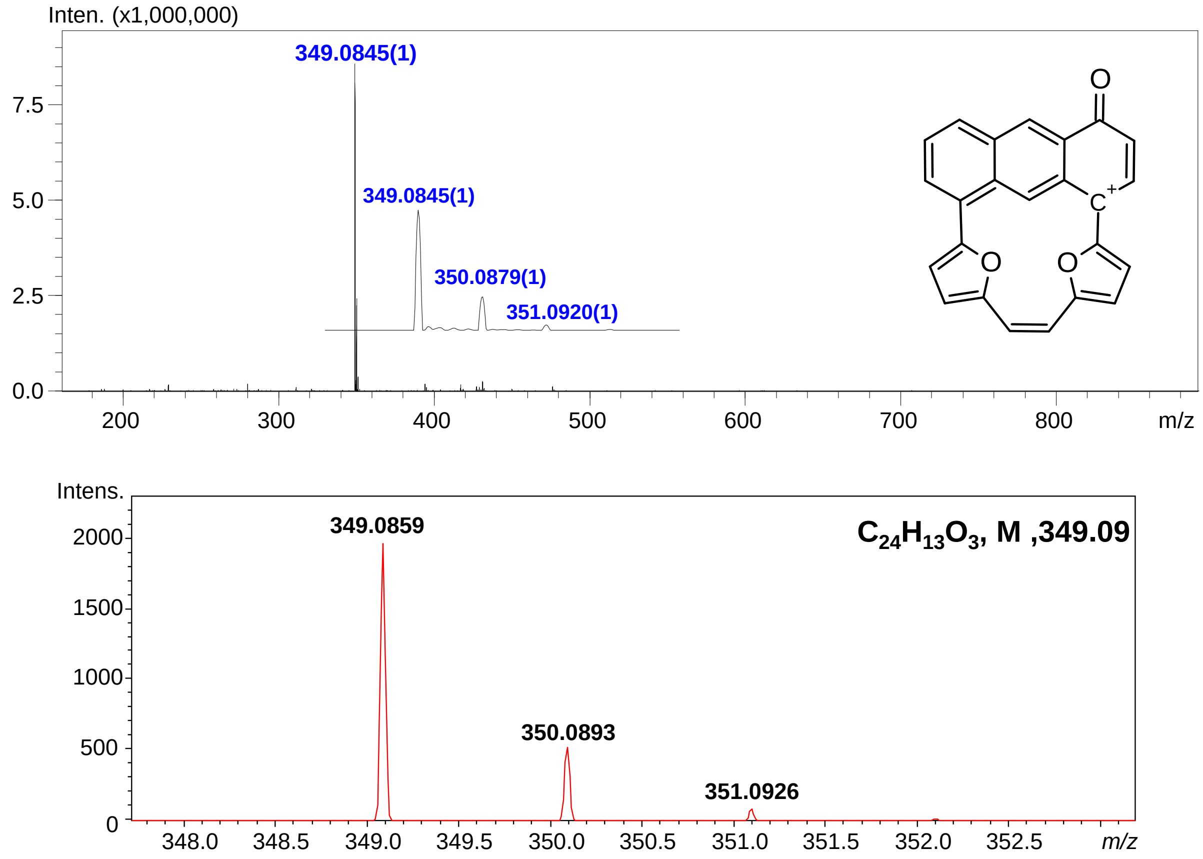

Fig. S61 ESI-MS spectrum of **4a** with simulated spectrum (bottom panel - simulated isotopic patterns for molecular formula of investigated compounds).

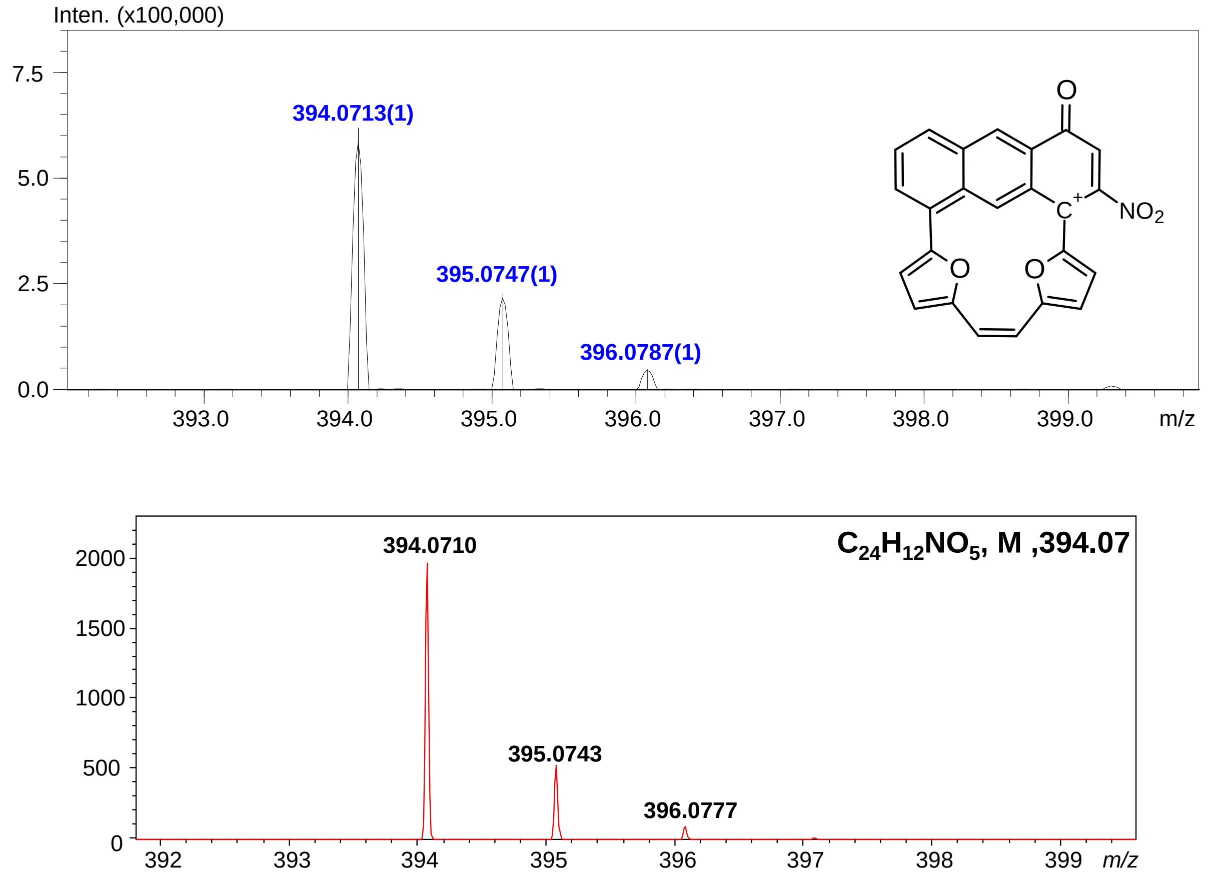

Fig. S62 ESI-MS spectrum of **5** (zoom) with simulated spectrum (bottom panel - simulated isotopic patterns for molecular formula of investigated compounds).

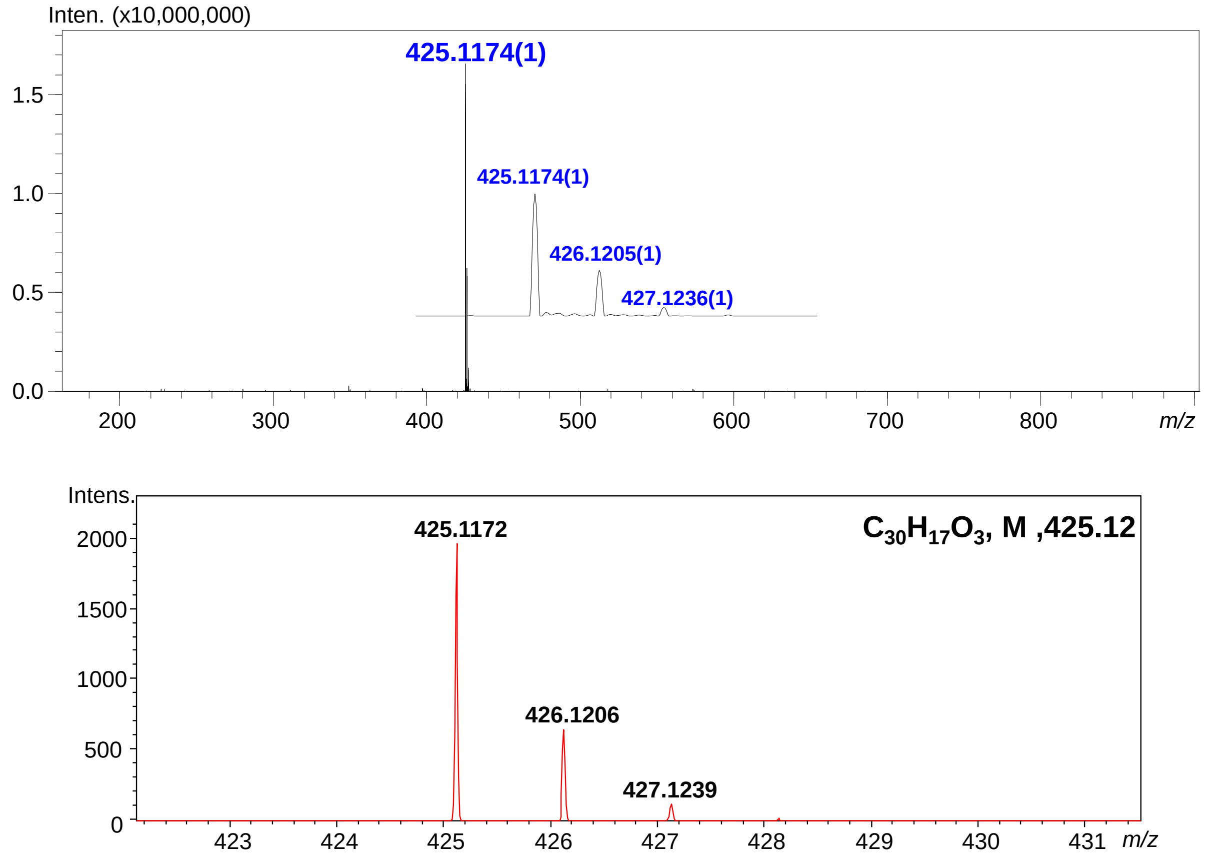

Fig. S63 ESI-MS spectrum of **4b** with simulated spectrum (bottom panel - simulated isotopic patterns for molecular formula of investigated compounds).

6. Theoretical calculation

6.1. NICS analysis

NICS(1) maps were calculated by generating a rectangular grid of dummy atoms (Bq) with 0.25 Å spacing, 1 Å above the mean plane of the molecule and running NMR chemical shift calculations in Gaussian16 software on a B3LYP 6-31G(d,p) level of theory.

Isotropic and *zz* magnetic components of shielding for all Bq atoms were extracted from the output files using authors' own script. Two-dimensional maps were generated using Origin software using a 'colour fill' contour plot option and scaling data to values between -25 and 25 (*isotropic* maps) and -50 and 50 (*zz* component maps) with 0.1 increment in value and using a 'rainbow' colour palette.

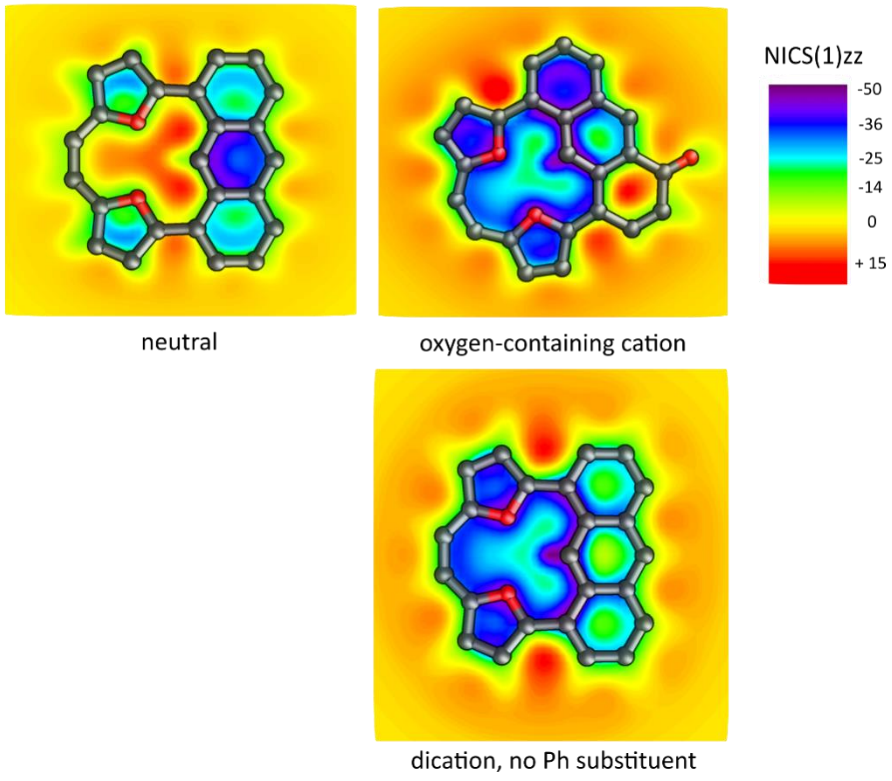

Fig. S64 NICS(1)zz maps for neutral macrocycles and their dications.

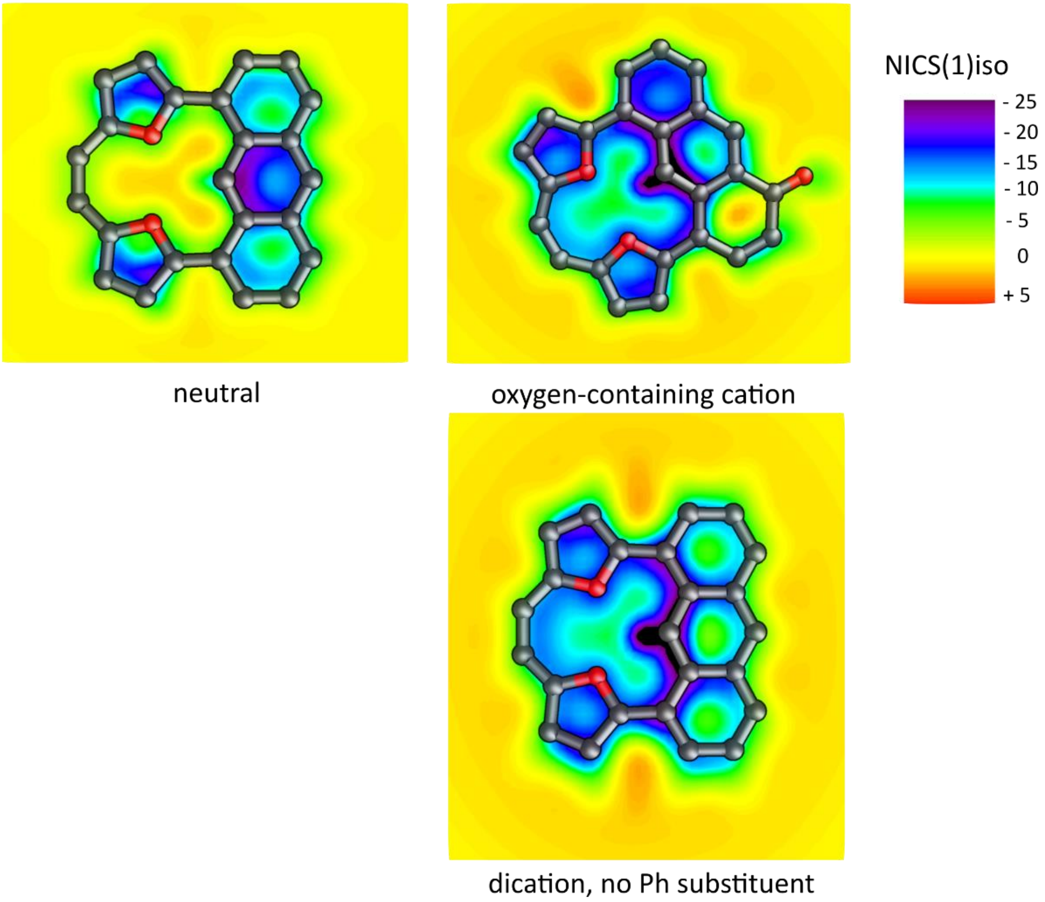

Fig. S65 NICS(1)iso maps for neutral macrocycles and their dications.

Table S1. Comparison of ^{1}H chemical shifts recorded and predicted with GIAO calculations.

	**1a**		**1b**		**$1b^{2+}$**	
	**Obs.**	**Theory**	**Obs.**	**Theory**	**Obs**	**Theory**
3	8.13	7.92	o-7.65,<br>m-7.59,<br>p- 7.55	o – 7.42/7.50<br>m – 7.72/7.74<br>p – 7.67	o – 7.99<br>m – 7.89<br>p – 7.96	o – 8.14/8.47<br>m – 8.30/8.36<br>p – 8.50
2^1,4^1	7.75	7.63	7.42	7.70	11.00	11.00
2^2, 4^2	7.34	7.52	7.28	7.22	9.92	10.35/10.12
6^1,17^1	7.56	7.73	7.43	7.40	9.20	9.51
8,15	6.49	7.16	7.02	7.17	10.50	10.49
9,14	6.90	6.53	6.60	6.57	10.20	10.23
11,12	6.05	5.69	6.14	5.80	9.91	10.21
18	12.61	14.12	12.71	12.83	0.9	-0.65

6.2. AICD plots.

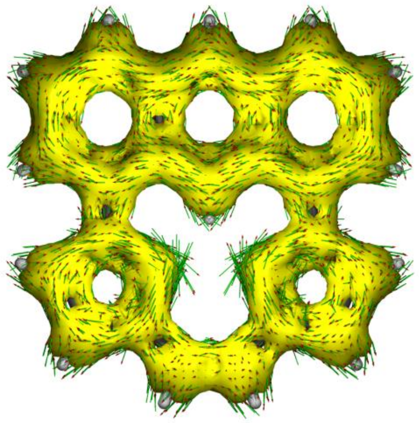

Figure S66. AICD Plot for **1a**

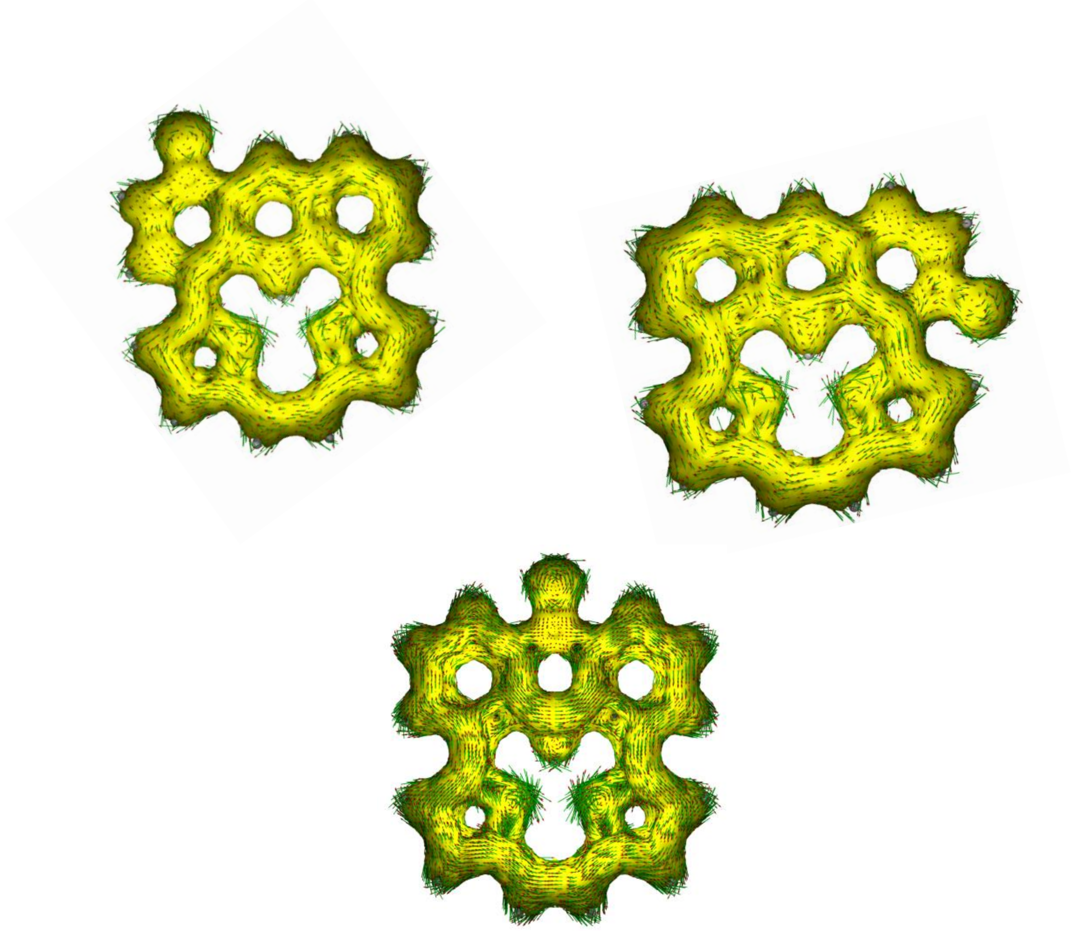

Figure S67. AICD Plot for **4a**, **4a'** and **4a''**

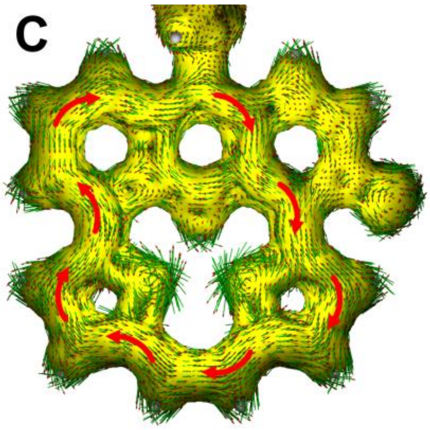

Figure S68. AICD plot of **4b**

6.3. Charge distribution

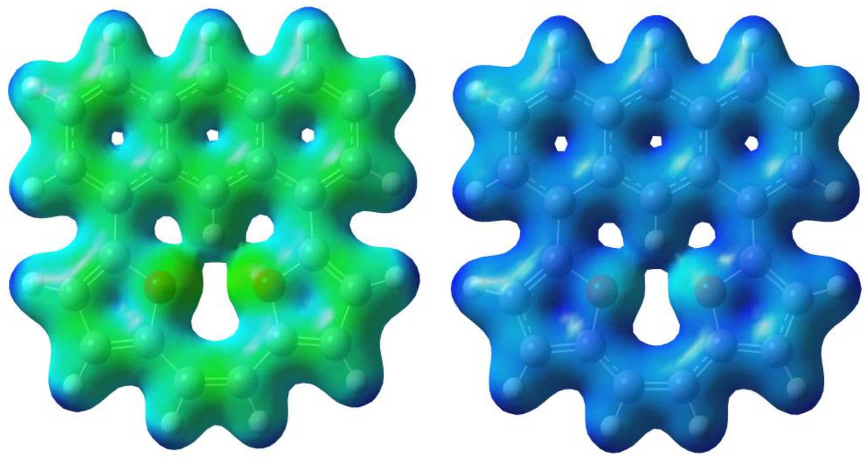

Figure S69. Charge distribution for **1a** and **1a^{2+}**

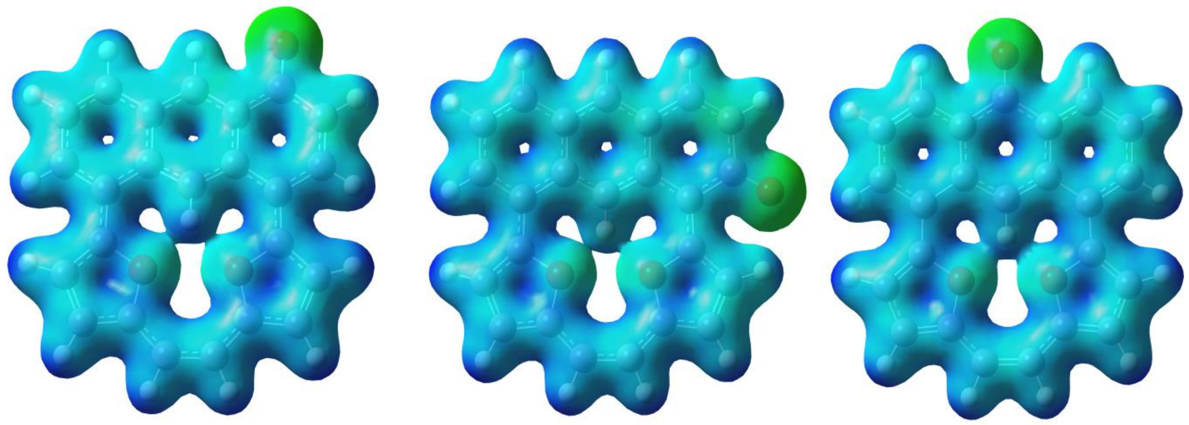

Figure S70. Charge distribution for **4a**, **4a’** and **4a’’**

6.4. AIM analysis

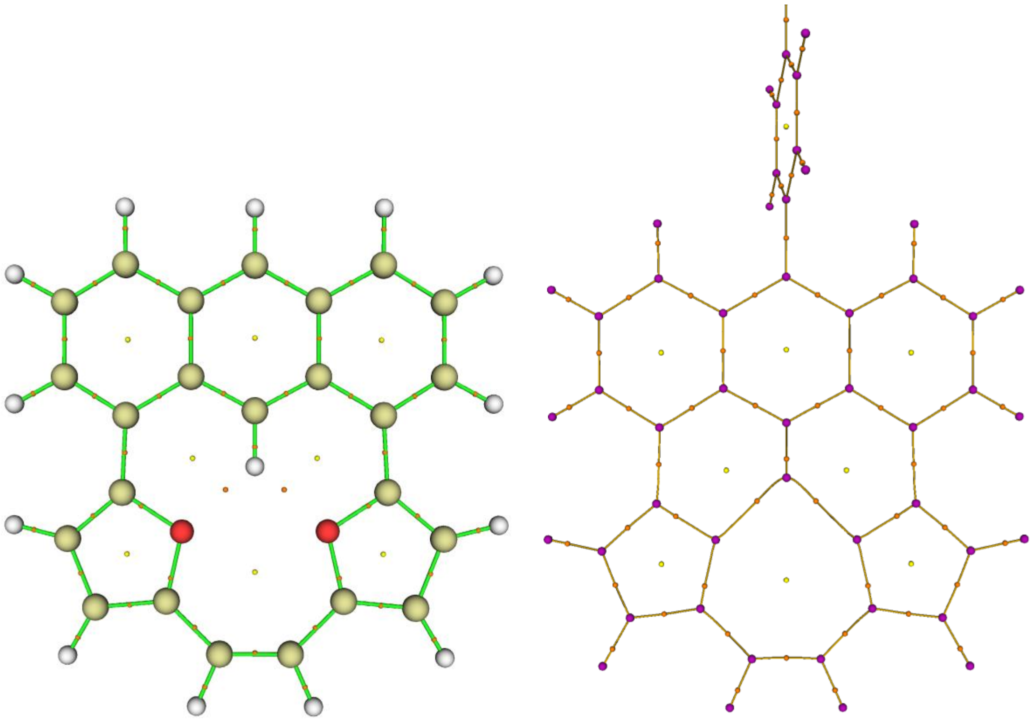

Figure S71. Critical points plot obtained for **1a** (A) and **1b** (B).

6.5. Cartesian coordinates

6.5.1. **1a.**

C	1.353000	-3.662000	-0.053000
C	0.621000	-2.485000	-0.027000
C	1.359000	-1.231000	-0.015000
C	2.802000	-1.234000	-0.019000
C	3.485000	-2.487000	-0.041000
C	2.771000	-3.659000	-0.060000
C	0.710000	-0.000000	0.000000
C	3.475000	-0.000000	0.000000
C	2.802000	1.234000	0.019000
C	1.359000	1.231000	0.015000
C	0.621000	2.485000	0.027000
C	1.353000	3.662000	0.053000
C	2.771000	3.659000	0.060000
C	3.485000	2.487000	0.041000
H	-0.350000	0.000000	-0.000000
H	0.840000	-4.618000	-0.071000
H	4.572000	-2.498000	-0.045000
H	3.292000	-4.612000	-0.080000
H	4.563000	-0.000000	0.000000
H	0.840000	4.618000	0.071000
H	3.292000	4.612000	0.080000
H	4.572000	2.498000	0.045000
C	-0.845000	-2.560000	0.004000
C	-1.727000	-3.623000	0.081000
O	-1.586000	-1.405000	-0.036000
C	-3.037000	-3.085000	0.091000
H	-1.465000	-4.669000	0.133000
C	-2.914000	-1.708000	0.023000
H	-3.965000	-3.636000	0.148000
C	-0.845000	2.560000	-0.004000
C	-1.727000	3.623000	-0.081000
O	-1.586000	1.405000	0.036000
C	-3.037000	3.085000	-0.091000
H	-1.465000	4.669000	-0.133000
C	-2.914000	1.708000	-0.023000
H	-3.965000	3.636000	-0.148000
C	-3.923000	-0.684000	0.012000
C	-3.923000	0.684000	-0.012000
H	-4.916000	-1.128000	0.027000
H	-4.916000	1.128000	-0.027000

Total energy: -1073.43241922,
Imaginary frequencies: 0

6.5.2. **1b**

C	0.116000	-3.658000	-0.005000
C	-0.598000	-2.475000	0.097000
C	0.146000	-1.227000	0.171000
C	1.586000	-1.233000	0.060000
C	2.251000	-2.495000	-0.031000
C	1.531000	-3.663000	-0.045000
C	-0.503000	-0.003000	0.319000
C	2.284000	0.001000	0.047000
C	1.584000	1.234000	0.081000
C	0.143000	1.224000	0.189000
C	-0.601000	2.472000	0.138000
C	0.112000	3.658000	0.069000
C	1.527000	3.667000	0.046000
C	2.249000	2.499000	0.037000
H	-1.554000	-0.005000	0.495000
H	-0.412000	-4.601000	-0.094000
H	3.332000	-2.520000	-0.103000
H	2.048000	-4.615000	-0.124000
H	-0.416000	4.603000	-0.001000
H	2.044000	4.621000	0.004000
H	3.331000	2.527000	-0.014000
C	-2.061000	-2.536000	0.073000
C	-2.949000	-3.586000	0.210000
O	-2.785000	-1.393000	-0.164000
C	-4.252000	-3.057000	0.051000
H	-2.693000	-4.615000	0.416000
C	-4.116000	-1.696000	-0.162000
H	-5.187000	-3.596000	0.109000
C	-2.065000	2.533000	0.104000
C	-2.954000	3.583000	0.235000
O	-2.786000	1.391000	-0.139000
C	-4.256000	3.054000	0.066000
H	-2.701000	4.612000	0.443000
C	-4.118000	1.693000	-0.147000
H	-5.191000	3.593000	0.117000
C	-5.125000	-0.685000	-0.309000
C	-5.125000	0.682000	-0.304000
H	-6.111000	-1.134000	-0.399000
H	-6.112000	1.131000	-0.391000
C	3.781000	0.002000	-0.025000
C	4.555000	-0.198000	1.128000
C	4.439000	0.202000	-1.249000
C	5.949000	-0.198000	1.060000
H	4.058000	-0.352000	2.082000
C	5.833000	0.202000	-1.318000
H	3.851000	0.356000	-2.149000
C	6.593000	0.002000	-0.163000
H	6.532000	-0.353000	1.964000
H	6.325000	0.357000	-2.273000
H	7.677000	0.002000	-0.216000

Total energy: -1304.49039839
Imaginary frequencies: 0

6.5.3. **1b^{2+}**

C	-0.120000	3.669000	-0.099000
C	0.607000	2.457000	-0.189000
C	-0.136000	1.219000	-0.265000
C	-1.567000	1.227000	-0.091000
C	-2.216000	2.473000	0.069000
C	-1.504000	3.671000	0.019000
C	0.506000	-0.000000	-0.457000
C	-2.284000	-0.012000	-0.051000
C	-1.558000	-1.246000	-0.103000
C	-0.125000	-1.224000	-0.255000
C	0.627000	-2.459000	-0.211000
C	-0.090000	-3.677000	-0.156000
C	-1.479000	-3.693000	-0.122000
C	-2.205000	-2.502000	-0.094000
H	1.533000	0.003000	-0.745000
H	0.410000	4.614000	-0.076000
H	-3.284000	2.501000	0.241000
H	-2.031000	4.613000	0.121000
H	0.446000	-4.618000	-0.122000
H	-2.004000	-4.640000	-0.103000
H	-3.287000	-2.546000	-0.073000
C	2.027000	2.524000	-0.060000
C	2.918000	3.619000	0.055000
O	2.746000	1.375000	0.041000
C	4.185000	3.098000	0.220000
H	2.652000	4.665000	0.021000
C	4.067000	1.685000	0.207000
H	5.110000	3.644000	0.343000
C	2.051000	-2.513000	-0.124000
C	2.964000	-3.595000	-0.158000
O	2.755000	-1.363000	0.051000
C	4.229000	-3.064000	-0.009000
H	2.714000	-4.636000	-0.294000
C	4.087000	-1.658000	0.113000
H	5.169000	-3.596000	0.007000
C	5.062000	0.718000	0.313000
C	5.072000	-0.686000	0.256000
H	6.045000	1.161000	0.438000
H	6.064000	-1.122000	0.320000
C	-3.760000	-0.012000	0.055000
C	-4.402000	-0.615000	1.154000
C	-4.542000	0.596000	-0.947000
C	-5.792000	-0.600000	1.250000
H	-3.810000	-1.059000	1.947000
C	-5.932000	0.587000	-0.854000
H	-4.060000	1.042000	-1.811000
C	-6.559000	-0.006000	0.245000
H	-6.275000	-1.049000	2.111000
H	-6.525000	1.040000	-1.641000
H	-7.642000	-0.004000	0.318000

Total energy: -1304.10095298
Imaginary frequencies: 0

6.5.4. **4a**

C	-0.616000	3.886000	-0.120000
C	-0.747000	2.515000	0.038000
C	0.462000	1.714000	0.111000
C	1.748000	2.343000	-0.042000
C	1.811000	3.763000	-0.180000
C	0.657000	4.509000	-0.204000
C	0.422000	0.329000	0.297000
C	2.892000	1.521000	-0.041000
C	2.824000	0.130000	0.037000
C	1.535000	-0.504000	0.168000
C	1.422000	-1.952000	0.147000
C	2.625000	-2.676000	0.093000
C	3.884000	-2.084000	0.044000
C	4.075000	-0.669000	-0.006000
H	-0.524000	-0.124000	0.492000
H	-1.505000	4.502000	-0.213000
H	2.782000	4.240000	-0.279000
H	0.707000	5.588000	-0.319000
H	3.880000	1.967000	-0.122000
H	2.575000	-3.763000	0.066000
H	4.773000	-2.709000	0.005000
C	-2.091000	1.926000	0.095000
C	-3.334000	2.460000	0.363000
O	-2.252000	0.586000	-0.180000
C	-4.279000	1.404000	0.255000
H	-3.542000	3.486000	0.629000
C	-3.574000	0.256000	-0.059000
H	-5.345000	1.464000	0.421000
C	0.158000	-2.660000	0.075000
C	-0.179000	-4.012000	0.068000
O	-1.005000	-1.942000	-0.067000
C	-1.582000	-4.095000	-0.087000
H	0.507000	-4.841000	0.162000
C	-2.074000	-2.803000	-0.159000
H	-2.178000	-4.997000	-0.128000
C	-4.033000	-1.101000	-0.212000
C	-3.421000	-2.327000	-0.252000
H	-5.119000	-1.139000	-0.255000
H	-4.112000	-3.162000	-0.345000
O	5.206000	-0.112000	-0.089000

Total energy: -1147.88812718
Imaginary frequencies: 0

6.5.5. **4a’**

C	3.556000	-1.993000	-0.042000
C	2.514000	-1.052000	-0.157000
C	1.153000	-1.541000	-0.172000
C	0.894000	-2.933000	0.074000
C	1.976000	-3.819000	0.196000
C	3.288000	-3.352000	0.112000
C	0.060000	-0.701000	-0.402000
C	-0.461000	-3.366000	0.166000
C	-1.522000	-2.485000	0.078000
C	-1.262000	-1.088000	-0.173000
C	-2.338000	-0.125000	-0.154000
C	-3.753000	-0.636000	-0.116000
C	-3.931000	-2.072000	0.103000
C	-2.892000	-2.931000	0.206000
H	0.246000	0.291000	-0.751000
H	4.586000	-1.658000	-0.031000
H	1.785000	-4.875000	0.360000
H	4.114000	-4.048000	0.205000
H	-0.657000	-4.421000	0.333000
H	-4.959000	-2.410000	0.176000
C	2.841000	0.345000	-0.127000
C	4.067000	1.034000	-0.175000
O	1.841000	1.255000	0.027000
C	3.779000	2.386000	-0.053000
H	5.043000	0.590000	-0.298000
C	2.380000	2.511000	0.066000
H	4.481000	3.207000	-0.052000
C	-2.136000	1.257000	-0.059000
C	-3.031000	2.365000	-0.009000
O	-0.856000	1.758000	0.057000
C	-2.274000	3.505000	0.122000
H	-4.102000	2.276000	-0.076000
C	-0.910000	3.111000	0.151000
H	-2.625000	4.524000	0.189000
C	1.601000	3.664000	0.188000
C	0.226000	3.919000	0.233000
H	2.215000	4.559000	0.235000
H	-0.029000	4.971000	0.317000
O	-4.731000	0.107000	-0.249000
H	-3.069000	-3.990000	0.374000

Total energy: -1147.88554328
Imaginary frequencies: 0

6.5.6. **4a’’**

C	1.146000	3.690000	0.035000
C	0.414000	2.472000	0.015000
C	1.177000	1.243000	0.010000
C	2.607000	1.282000	0.013000
C	3.266000	2.494000	0.029000
C	2.527000	3.698000	0.042000
C	0.541000	0.000000	0.000000
C	3.381000	0.000000	-0.000000
C	2.607000	-1.282000	-0.013000
C	1.177000	-1.243000	-0.010000
C	0.414000	-2.472000	-0.015000
C	1.146000	-3.690000	-0.035000
C	2.527000	-3.698000	-0.042000
C	3.266000	-2.494000	-0.029000
H	-0.520000	0.000000	0.000000
H	0.614000	4.634000	0.050000
H	4.351000	2.507000	0.032000
H	3.053000	4.646000	0.058000
H	0.614000	-4.634000	-0.050000
H	3.053000	-4.646000	-0.058000
H	4.351000	-2.507000	-0.032000
C	-1.020000	2.539000	-0.004000
C	-1.918000	3.617000	-0.049000
O	-1.756000	1.386000	0.013000
C	-3.206000	3.088000	-0.059000
H	-1.656000	4.664000	-0.079000
C	-3.084000	1.689000	-0.022000
H	-4.137000	3.635000	-0.093000
C	-1.020000	-2.539000	0.004000
C	-1.918000	-3.617000	0.049000
O	-1.756000	-1.386000	-0.013000
C	-3.206000	-3.088000	0.059000
H	-1.656000	-4.664000	0.079000
C	-3.084000	-1.689000	0.022000
H	-4.137000	-3.635000	0.093000
C	-4.084000	0.694000	-0.013000
C	-4.084000	-0.694000	0.013000
H	-5.077000	1.133000	-0.029000
H	-5.077000	-1.133000	0.029000
O	4.609000	0.000000	-0.000000

Total energy: -1147.87532367
Imaginary frequencies: 0

6.5.7. **4b.**

C	0.112000	3.611000	-0.202000
C	-0.630000	2.303000	-0.205000
C	0.134000	1.076000	-0.267000
C	1.567000	1.083000	-0.085000
C	2.230000	2.365000	0.058000
C	1.558000	3.536000	-0.012000
C	-0.498000	-0.149000	-0.477000
C	2.274000	-0.120000	-0.053000
C	1.573000	-1.379000	-0.138000
C	0.144000	-1.375000	-0.299000
C	-0.590000	-2.623000	-0.275000
C	0.140000	-3.826000	-0.242000
C	1.529000	-3.816000	-0.185000
C	2.239000	-2.617000	-0.113000
H	-1.524000	-0.149000	-0.771000
H	3.303000	2.371000	0.209000
H	2.071000	4.488000	0.071000
H	-0.382000	-4.776000	-0.226000
H	2.068000	-4.757000	-0.164000
H	3.319000	-2.642000	-0.038000
C	-2.019000	2.346000	-0.036000
C	-2.944000	3.427000	0.068000
O	-2.735000	1.177000	0.117000
C	-4.192000	2.888000	0.268000
H	-2.667000	4.464000	-0.012000
C	-4.049000	1.475000	0.284000
H	-5.127000	3.416000	0.389000
C	-2.019000	-2.691000	-0.168000
C	-2.917000	-3.775000	-0.192000
O	-2.728000	-1.551000	0.051000
C	-4.187000	-3.257000	0.013000
H	-2.661000	-4.811000	-0.354000
C	-4.055000	-1.861000	0.156000
H	-5.119000	-3.802000	0.053000
C	-5.043000	0.503000	0.416000
C	-5.043000	-0.893000	0.352000
H	-6.025000	0.943000	0.561000
H	-6.029000	-1.340000	0.448000
C	3.762000	-0.125000	0.088000
C	4.579000	0.256000	-0.989000
C	4.365000	-0.514000	1.295000
C	5.969000	0.247000	-0.860000
H	4.123000	0.552000	-1.928000
C	5.754000	-0.516000	1.423000
H	3.742000	-0.805000	2.135000
C	6.559000	-0.137000	0.346000
H	6.588000	0.541000	-1.701000
H	6.206000	-0.813000	2.364000
H	7.640000	-0.142000	0.446000
O	-0.455000	4.701000	-0.329000

Total energy: -1378.94309703
Imaginary frequencies: 0

7. X-Ray Analysis

Identification code	MJP_Anth (**1a**)	MJP_AD (**1b**)
CCDC Number	**2114822**	**2114821**
Empirical formula	C24H14O2	C31.5H21.5O2
Formula weight	334.35	431.99
Temperature/K	100(2)	100(2)
Crystal system	Monoclinic	Triclinic
Space group	P21/c	P-1
a/Å	30.5698(9)	11.9695(11)
b/Å	12.3953(3)	12.5935(11)
c/Å	12.3088(3)	14.471(2)
α/°		97.174(9)
β/°	97.343(2)	102.702(10)
γ/°		90.760(7)
Volume/Å^3	4625.8(2)	2109.5(4)
Z	12	4
ρ_{calc} g/cm^3	1.440	1.360
μ/mm^{-1}	0.091	0.084
F(000)	2088.0	906.0
Radiation	MoK$_\alpha$ (λ = 0.71073)	MoK$_\alpha$ (λ = 0.71073)
2Θ range for data collection/°	6.638 to 60.168	6.606 to 58.754
Index ranges	40 ≤ h ≤ 40, -16 ≤ k ≤ 17, -16 ≤ l ≤ 15	-16 ≤ h ≤ 15, -15 ≤ k ≤ 17, -18 ≤ l ≤ 19
Reflections collected	23998	18132
Independent reflections	11922 [R_{int} = 0.0233, R_{sigma} = 0.0398]	9804 [R_{int} = 0.1024, R_{sigma} = 0.3010]
Data/restraints/parameters	11922/0/715	9804/0/605
Goodness-of-fit on F2	1.018	0.912
Final R indexes [I>=2σ (I)]	R1 = 0.0555, wR2 = 0.1288	R1 = 0.0863, wR2 = 0.1254
Final R indexes [all data]	R1 = 0.0791, wR2 = 0.1421	R1 = 0.2964, wR2 = 0.2003
Largest diff. peak/hole / e Å^{-3}	0.67/-0.21	0.33/-0.22

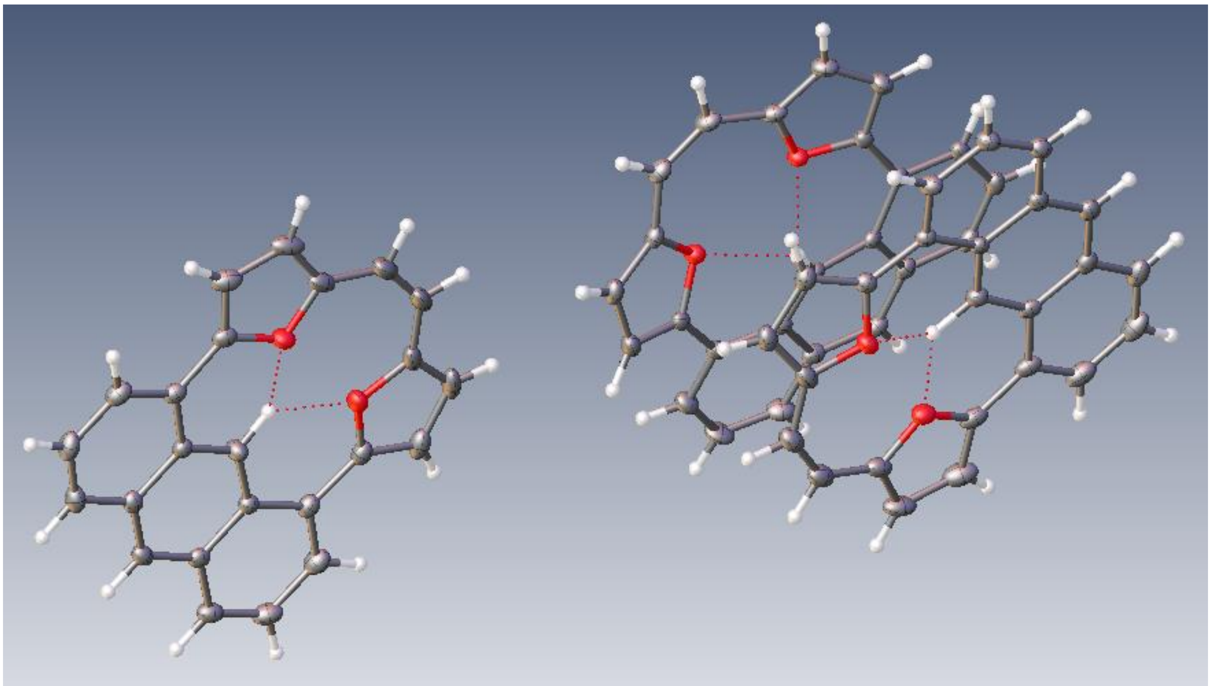

Figure S72. Crystal structure of **1a**. Thermal ellipsoids present 50% probability.

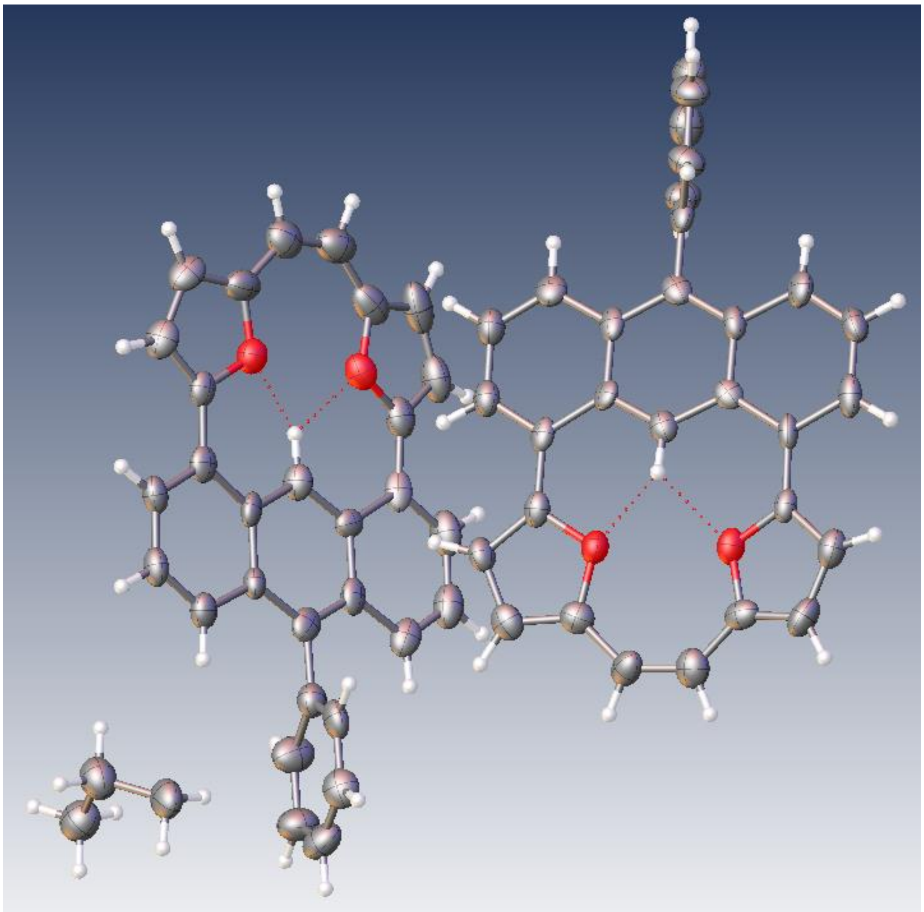

Figure S73. Crystal structure of **1a**. Thermal ellipsoids present 50% probability.
